# The Plasma Environment of Comet 67P/Churyumov-Gerasimenko

**DOI:** 10.1007/s11214-022-00931-1

**Published:** 2022-11-10

**Authors:** Charlotte Goetz, Etienne Behar, Arnaud Beth, Dennis Bodewits, Steve Bromley, Jim Burch, Jan Deca, Andrey Divin, Anders I. Eriksson, Paul D. Feldman, Marina Galand, Herbert Gunell, Pierre Henri, Kevin Heritier, Geraint H. Jones, Kathleen E. Mandt, Hans Nilsson, John W. Noonan, Elias Odelstad, Joel W. Parker, Martin Rubin, Cyril Simon Wedlund, Peter Stephenson, Matthew G. G. T. Taylor, Erik Vigren, Sarah K. Vines, Martin Volwerk

**Affiliations:** 1 ESTEC, European Space Agency, Keplerlaan 1, 2201 AZ Noordwijk, The Netherlands; 2 Department of Mathematics, Physics and Electrical Engineering, Northumbria University, Newcastle-upon-Tyne, UK; 3 Swedish Institute of Space Physics, Box 812, 981 28 Kiruna, Sweden; 4 Lagrange, OCA, UCA, CNRS, Nice, France; 5 Department of Physics, Umeå University, 901 87 Umeå, Sweden; 6 Physics Department, Leach Science Center, Auburn University, Auburn, AL 36832 USA; 7 Southwest Research Institute, P.O. Drawer 28510, San Antonio, TX 78228-0510 USA; 8 Laboratory for Atmospheric and Space Physics, University of Colorado Boulder, 3665 Discovery Drive, Boulder, CO 80303 USA; 9 Earth Physics Department, St. Petersburg State University, Ulianovskaya, 1, St Petersburg, 198504 Russia; 10 Swedish Institute of Space Physics, Box 537, SE-751 21 Uppsala, Sweden; 11 Department of Physics and Astronomy, Johns Hopkins University, Baltimore, MD 21218 USA; 12 Department of Physics, Imperial College London, Prince Consort Road, London, SW7 2AZ UK; 13 LPC2E, CNRS, Orléans, France; 14 UCL Mullard Space Science Laboratory, Holmbury St. Mary, Dorking, RH5 6NT UK; 15 The Centre for Planetary Sciences at UCL/Birkbeck, Gower Street, London, WC1E 6BT UK; 16 Johns Hopkins Applied Physics Laboratory, Laurel, MD 20728 USA; 17 Lunar and Planetary Laboratory, University of Arizona, Tucson, AZ 85719 USA; 18 Southwest Research Institute, Boulder, CO 80302 USA; 19 Physikalisches Institut, University of Bern, Sidlerstrasse 5, 3012 Bern, Switzerland; 20 Space Research Institute, Austrian Academy of Sciences, Schmiedlstr. 6, 8042 Graz, Austria; 21 Johns Hopkins Applied Physics Laboratory, Laurel, MD 20723 USA

## Abstract

The environment of a comet is a fascinating and unique laboratory to study plasma processes and the formation of structures such as shocks and discontinuities from electron scales to ion scales and above. The European Space Agency’s Rosetta mission collected data for more than two years, from the rendezvous with comet 67P/Churyumov-Gerasimenko in August 2014 until the final touch-down of the spacecraft end of September 2016. This escort phase spanned a large arc of the comet’s orbit around the Sun, including its perihelion and corresponding to heliocentric distances between 3.8 AU and 1.24 AU. The length of the active mission together with this span in heliocentric and cometocentric distances make the Rosetta data set unique and much richer than sets obtained with previous cometary probes. Here, we review the results from the Rosetta mission that pertain to the plasma environment. We detail all known sources and losses of the plasma and typical processes within it. The findings from in-situ plasma measurements are complemented by remote observations of emissions from the plasma. Overviews of the methods and instruments used in the study are given as well as a short review of the Rosetta mission. The long duration of the Rosetta mission provides the opportunity to better understand how the importance of these processes changes depending on parameters like the outgassing rate and the solar wind conditions. We discuss how the shape and existence of large scale structures depend on these parameters and how the plasma within different regions of the plasma environment can be characterised. We end with a non-exhaustive list of still open questions, as well as suggestions on how to answer them in the future.

## Introduction

The past several years have brought incredible advancements in our understanding of the plasma around comets, largely thanks to the European Space Agency’s Rosetta spacecraft. Its two year mission orbiting comet 67P/Churyumov-Gerasimenko (67P) has provided a large amount of observations of the nucleus and its environment over approximately half of the comet’s orbit. It has allowed, for the first time, to study the evolution of the neutral and plasma environment from a relatively inactive stage to a full-fledged cometary coma. With the touchdown of the lander Philae on the surface and the eventual descent of Rosetta to the nucleus, this is also the closest a spacecraft has ever come to a comet while observing the plasma.

**Fig. 1:**
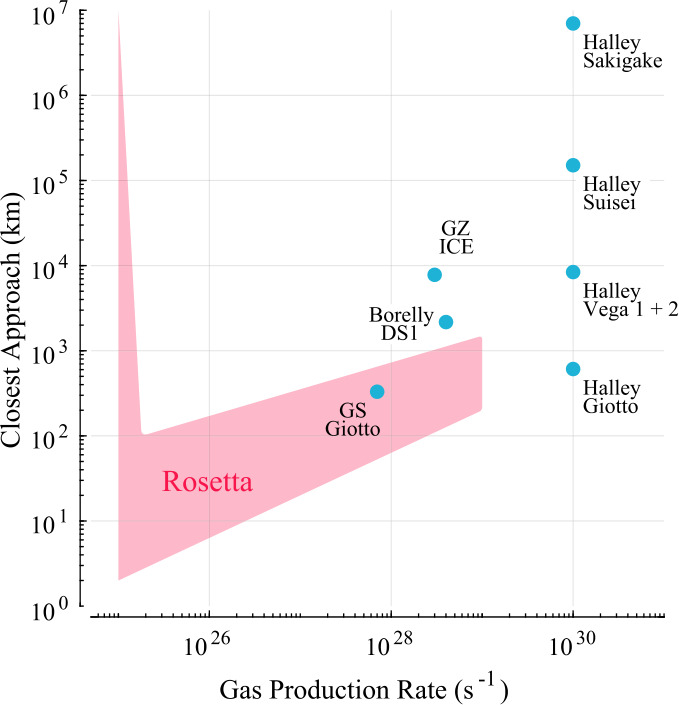
Closest approach distance vs gas production rate for the spacecraft that have visited comets. The gas production rate was derived from in-situ observations. The shaded, red area indicates the approximate range of distances and gas production rates covered by Rosetta during its two year mission. Abbreviations: GZ: 21P/Giacobini-Zinner, ICE: International Cometary Explorer, DS1: Deep Space 1, GS: 26P/Grigg-Skjellerup. Values are from Gringauz et al. (1986b), Johnstone et al. (1993), Richter et al. (2011), Goetz (2019), Cowley (1987)

**Fig. 2:**
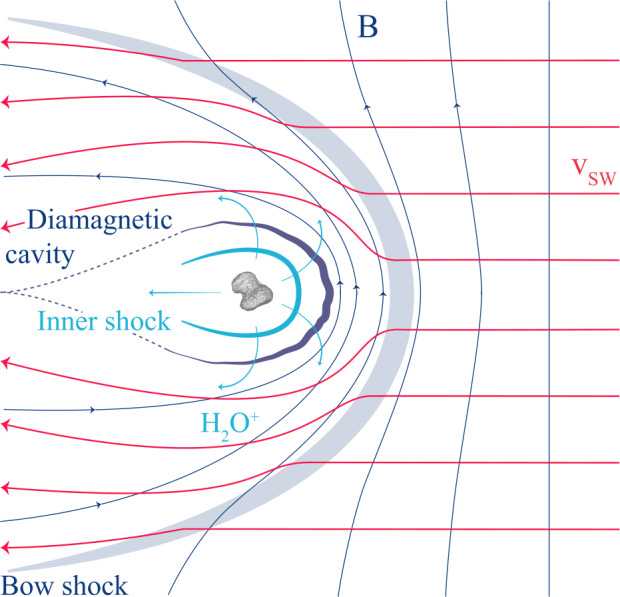
View of the interaction pre-*Rosetta*, for a highly active comet such as 1P/Halley. The Sun is on the right, $v_{\text{sw}}$ and $B$ denote the solar wind flow velocity and the interplanetary magnetic field. The comet is outgassing water that is quickly turned into $\text{H}_{2}\text{O}^{+}$. The water ions expand from the comet and create an inner shock where the expansion speed exceeds the acoustic speed. A diamagnetic cavity is formed and the interplanetary magnetic field drapes around the inner coma. The solar wind flow is deflected. For more details see Coates and Jones (2009)

Other comets have been visited before, most famously, an armada of spacecraft flew by comet 1P/Halley in 1986. Figure 1 shows an approximate overview of the gas production rates of the visited comets and the closest approach distance of the spacecraft. It becomes clear that Rosetta is unique in the sense that it is covering not only a large range of distances and gas production rates, but it also covers a previously largely unexplored region of the parameter space. Thus, this paper shall focus on the new results obtained by Rosetta and the reader is referred to earlier works such as Festou et al. (2004) and Grewing et al. (1988) for results from other missions. Figure 2 shows an overview of what the plasma environment of comets was thought to look like before Rosetta arrived at comet 67P. We will detail below when this previous picture is most applicable to the situation at 67P and when it needs to be revised so as to reflect the uniqueness of the new measurements.

The comet underwent large variations in gas production rate and heliocentric distance, as it travelled through the solar system, approached the Sun with perihelion on 13 August 2015 and then moved towards the outer solar system again. Therefore, it is advantageous to define certain stages in the comet’s development. This aids in defining common parameters that are comparable between pre- and post-perihelion times, it should be noted though, that the demarcation between these stages is fluid and they can often be observed within hours of each other. Three stages are used: the weakly, intermediately and strongly active stage.

The **strongly active stage** for gas production rates $Q>5\times10^{27}~\text{s}^{-1}$ is perhaps the most recognizable, as this is a stage that is most comparable to the plasma around previously visited comets like 1P/Halley. Here, traditional fluid-like plasma models can describe the interaction quite well on large scales, however many differences remain between this stage and the extremely active comets represented by 1P/Halley. Comet 67P was in this stage from approximately June 2016 to November 2016. The **intermediately active stage** for $10^{26}< Q<5\times 10^{27}~\text{s}^{-1}$ (approx. pre-perihelion January 2015 to May 2015 and post-perihelion December 2015 to April 2016) is characterized by an interplay of fluid effects and finite ion gyroradius effects and gives rise to a host of new phenomena never explored before. The **weakly active stage** with $Q<10^{26}~\text{s}^{-1}$ is marked by large ion gyroradius effects and very limited fluid-like behaviour. This stage is observable from August 2014 to end of 2014 and from May 2016 to September 2016.

The old view on cometary plasma environments shown in Fig. 2 is most applicable at the high activity stage, although, as will be explored in this review, even then remarkable differences can be found. For a general overview of high activity comets see for example Cravens and Gombosi (2004), Gombosi (2015) or Ip (2004).

While these three activity stages provide a rough classification for the important spatial scales at a comet, there are also effects on smaller scales that play a role. For example, space charge effects can create significant large scale electric fields, and there are waves on both electron and ion time scales that affect e.g. plasma heating and energy transport. Therefore, a comet offers a unique opportunity to study plasma physics at scales both great and small, as well as cross-scale effects.

To observe the plasma environment and its sources and dynamics, Rosetta carries a bespoke suite of plasma instruments as well as a neutral gas monitor and an EUV spectrometer. All data and user guides describing data treatment and caveats may be found on the Planetary Science Archive (PSA^1^). These provide the basis for most of the new findings detailed later on.

The aim of this review is to provide a comprehensive overview of the recent advances in cometary plasma physics, with the focus on the plasma around comet 67P. First, we will address the physical processes that lead to the formation of a neutral and ionized coma (Sect. 2). The processes that distribute energy and momentum throughout the regions will be discussed, as well as the impact on emissions, chemistry and dust. Then, in Sect. 3, follows a short introduction into observations, specifically those made by Rosetta (Sect. 3.1.1), and the methods commonly used to interpret them. Lastly, we focus on the macroscopic structures and large regions with similar plasma parameters that could be observed (Sect. 4). We close with an overview of remaining open questions and an outlook to future investigations in Sect. 5.

## Plasma

### Plasma Sources

Fundamentally, there are two particle populations that shape the behaviour of the plasma at a comet: the neutral gas background and the incoming solar wind plasma. Any variation in parameters of either will have consequences on plasma processes, structures and boundaries. Thus, the better the understanding of the underlying parameters, the more we can learn about the cometary plasma.

#### Neutral Background

Upon arrival at comet 67P/Churyumov-Gerasimenko, its neutral gas coma was revealed by the various instruments on board Rosetta. A host of volatiles has been detected by the payload instruments on Rosetta and the lander Philae, namely by ROSINA (Rosetta Orbiter Spectrometer for Ion and Neutral Analysis, Balsiger et al. 2007), VIRTIS (Visible and InfraRed Thermal Imaging Spectrometer, Coradini et al. 2007), MIRO (Microwave Instrument for the Rosetta Orbiter, Gulkis et al. 2007), Alice (Stern et al. 2007), Ptolemy (Wright et al. 2007), and COSAC (Cometary Sampling and Composition experiment, Goesmann et al. 2007).

Outgassing of H_2_O, the major volatile, and a suite of lesser abundant species were measured by several instruments. VIRTIS detected H_2_O, CO_2_, CH_4_, and OCS (Bockelée-Morvan et al. 2016). MIRO observed H_2_O, CO, CH_3_OH, and NH_3_ (Biver et al. 2019). Measurements from Alice inferred the presence of H_2_O, CO_2_, and O_2_ (Keeney et al. 2017; Feldman et al. 2015). ROSINA detected the most abundant species H_2_O, CO_2_, CO, and O_2_ (Hässig et al. 2015; Bieler et al. 2015a). Also a host of other volatiles have been detected; however, their density abundances were generally low at the few percent level or even below (Le Roy et al. 2015). A complete list can be found in Altwegg et al. (2019) and Rubin et al. (2019). A subset of these species were also observed by the lander payload instruments Ptolemy (Wright et al. 2015) and COSAC (Goesmann et al. 2015) near the surface of the comet on 12 November 2014.

The obliquity of the comet’s rotation axis of 52 degrees combined with its complex bi-lobed shape (Sierks et al. 2015) led to a strong seasonal outgassing pattern (Hässig et al. 2015) and therefore a highly heterogeneous gas coma. Early on in the mission, beyond 3 AU from the Sun, the subsolar point was above the northern hemisphere. Inbound equinox occurred in May 2015 and started a short but intense summer in the south for the few months around perihelion in August 2015 at 1.24 AU until the outbound equinox in March 2016. H_2_O seemed to follow the seasonal illumination on the comet’s nucleus (Bieler et al. 2015b; Fougere et al. 2016a; Marschall et al. 2016, 2017). Species of higher volatility, on the other hand, were released more uniformly in the case of CO whereas CO_2_ release was enhanced above the southern hemisphere (Fougere et al. 2016b; Biver et al. 2019). O_2_ on the other hand (Bieler et al. 2015a) exhibited a strong correlation with water, despite the vastly different sublimation temperature of the pure ice equivalents. Corresponding trapping and release experiments in the lab showed the same outcome (Laufer et al. 2018).

Averaged over the whole Rosetta mission the southern hemisphere was the origin for the major part of the total outgassing (Läuter et al. 2019) and was thus subject to larger degrees of erosion (Keller et al. 2017). The northern hemisphere, on the other hand, shows smooth terrain, most likely the result of back-fall of wet grains (Rubin et al. 2014a; Thomas et al. 2015) that occurred over the previous perihelion passages. It is still debated to which degree this material, as opposed to the fresh material in the south, exhibited lower abundances of highly volatile species, possibly due to outgassing occurring during the transport from the south to the north. Nevertheless, there are indications that wet grains did act as distributed sources (De Keyser et al. 2017), even though their importance in terms of the total outgassing remained limited (Biver et al. 2019).

**Fig. 3:**
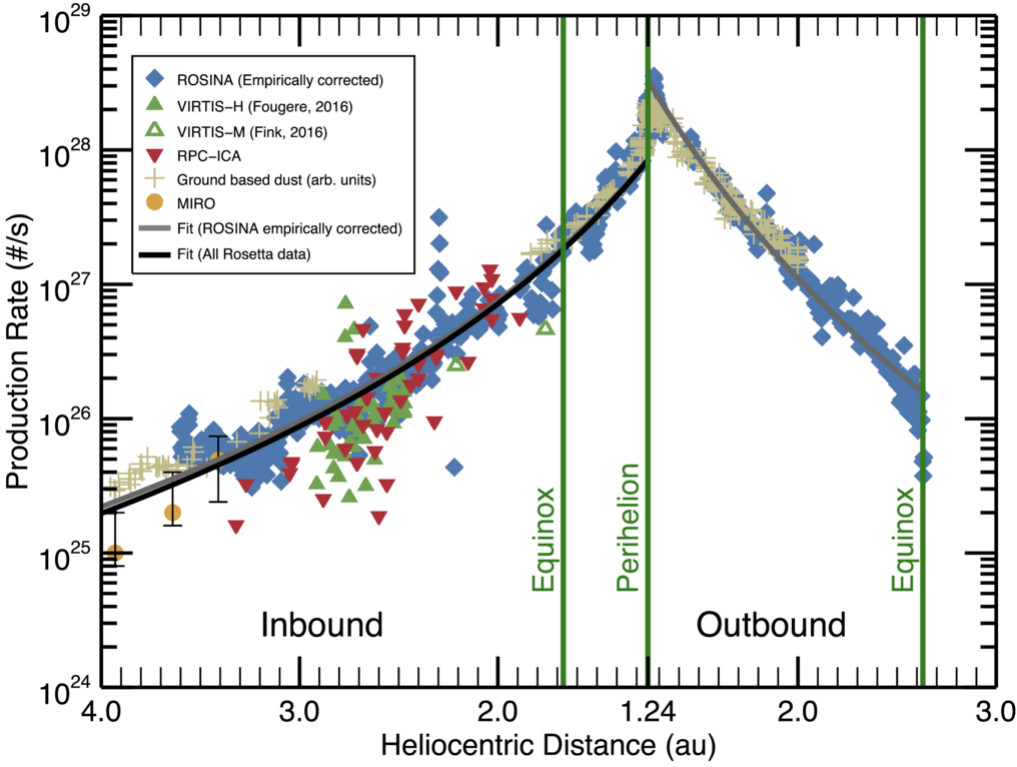
Water production rate as a function of heliocentric distance reproduced from Hansen et al. (2016), Fig. 9. The results from several Rosetta instruments have been added and include ROSINA (blue diamonds; Hansen et al. 2016), VIRTIS (green triangles; Bockelée-Morvan et al. 2015; Fink et al. 2016; Fougere et al. 2016a), RPC/ICA (red triangles; Simon Wedlund et al. 2016), MIRO (yellow circles; Biver et al. 2015; Lee et al. 2015), and the scaled dust brightness from ground-based observations for comparison (tan crosses; Snodgrass et al. 2016)

Rosetta monitored the total activity of the comet throughout the perihelion passage. Hansen et al. (2016) and Combi et al. (2020) presented the evolution of the water production rate based on the Direct Simulation Monte Carlo model by Fougere et al. (2016a), starting from Rosetta’s arrival at the comet in August 2014 all the way to the outbound equinox in March 2016. Figure 3 shows the water production rate reproduced from Hansen et al. (2016). Within this time frame the water outgassing varied by approximately 3 orders of magnitude, with a peak water production on the order of $10^{28}~\text{s}^{-1}$ some 2 to 3 weeks after the 13 August 2015 perihelion passage based on Rosetta measurements of MIRO (Marshall et al. 2017), VIRTIS (Bockelée-Morvan et al. 2015, 2016), ROSINA (Hansen et al. 2016; Läuter et al. 2019; Combi et al. 2020), RPC-ICA (Simon Wedlund et al. 2016), and observations of the Lyman Alpha Imaging Camera (LAICA) on the PROCYON (Proximate Object Close Flyby with Optical Navigation) spacecraft (Shinnaka et al. 2017). Hansen et al. (2016) furthermore showed that ground-based measurements of 67P’s dust brightness (Snodgrass et al. 2016) revealed a dust activity pattern very similar to the neutral gas.

As shown in Fig. 3, Simon Wedlund et al. (2016), and later Simon Wedlund et al. (2019a), have shown that it is possible to use the ion spectrometer RPC-ICA on board Rosetta as a remote sensing tool to infer the neutral outgassing rate^2^ from the knowledge of local solar wind charge-exchange processes (see also Sect. 3.2). Fluxes of solar wind $\text{He}^{2+}$ ions were first compared to those of $\text{He}^{+}$ ions, their charge-exchanged counterpart. This ratio was used in combination with up-to-date H_2_O charge-transfer cross sections (Simon Wedlund et al. 2019) and an evaluation of the column density along the Sun-comet line based on the classic model of Haser (1957). The so-called Haser model simply assumes a spherically-symmetric homogeneous outgassing of species $s$ at a constant neutral radial velocity $U_{n,s}$ ($\text{m}\,\text{s}^{-1}$):

1 $$ n_{s}(r) = \frac{Q_{0,s}}{4\pi \,U_{n,s}\,r^{2}}\ e^{-\left (r-r_{c} \right )\,\frac{k_{p,s}}{U_{n}}}, $$

where $n_{s}$ is the neutral density of species $s$ ($\text{m}^{-3}$), $Q_{0,s}$ the total outgassing rate ($\text{s}^{-1}$), $r$ and $r_{c}$ the cometocentric distance and nucleus radius, assumed spherical symmetric (in m), and $k_{p,s}$ is the total photo-destruction rate (photo-ionisation and photo-dissociation, in $\text{s}^{-1}$) of species $s$. Marshall et al. (2019) pointed out that outgassing retrievals based on this simple Haser model can only accidentally be predictive since comet 67P’s outgassing is highly asymmetric and depends on nucleus shape,^3^ spin axis, and activity distribution at the surface (as it is the case for many other cometary objects). However, because charge exchange is a cumulative process and depends on the integrated column of atmosphere along the line of sight:

2 $$ n_{\text{col},s}(r,\chi ) = \frac{Q_{0,s}}{4\pi \,U_{n,s}}\,\frac{\chi}{r\,\sin{\chi}}, $$

where $n_{\text{col},s}$ is the column density (in $\text{m}^{-2}$) and $\chi $ (in radian) is the solar zenith angle (Beth et al. 2016), the approach may still be valid outside of times when Rosetta went much deeper into the atmosphere of the comet, and outgassing asymmetries became dominant. The local outgassing retrievals based on solar wind ion charge exchange were shown to be within a factor $2\text{--}3$ on average of the MIRO and ROSINA-COPS observations, especially if the values were averaged over more than a full comet’s rotation (Simon Wedlund et al. 2019b) as shown in Fig. 3.

A similar analysis as that of Hansen et al. (2016), based on an analytic outgassing model, has been performed by Läuter et al. (2019) for the four major species H_2_O, CO_2_, CO, and O_2_. The different models show consistent results and reveal asymmetries in the outgassing behaviour inbound and outbound as a function of heliocentric distance. This is in line with the results presented by Gasc et al. (2017): a steeper decrease of water compared to more volatile species such as CO and CO_2_ has been observed. Independent of whether the individual species’ production rates are integrated over the whole Rosetta mission or studied close to their peak production near perihelion, water is the dominant volatile followed by carbon dioxide (Bockelée-Morvan et al. 2016; Läuter et al. 2019; Combi et al. 2020).

#### Solar Wind

An important input parameter for the plasma at 67P is the solar wind. Changes in its key parameters affect e.g. boundaries, plasma production and loss, magnetic field direction and magnitude (see also Sect. 2.4). Thus it is opportune to introduce key parameters for the solar wind that comet 67P encountered while accompanied by the Rosetta spacecraft.

**Fig. 4:**
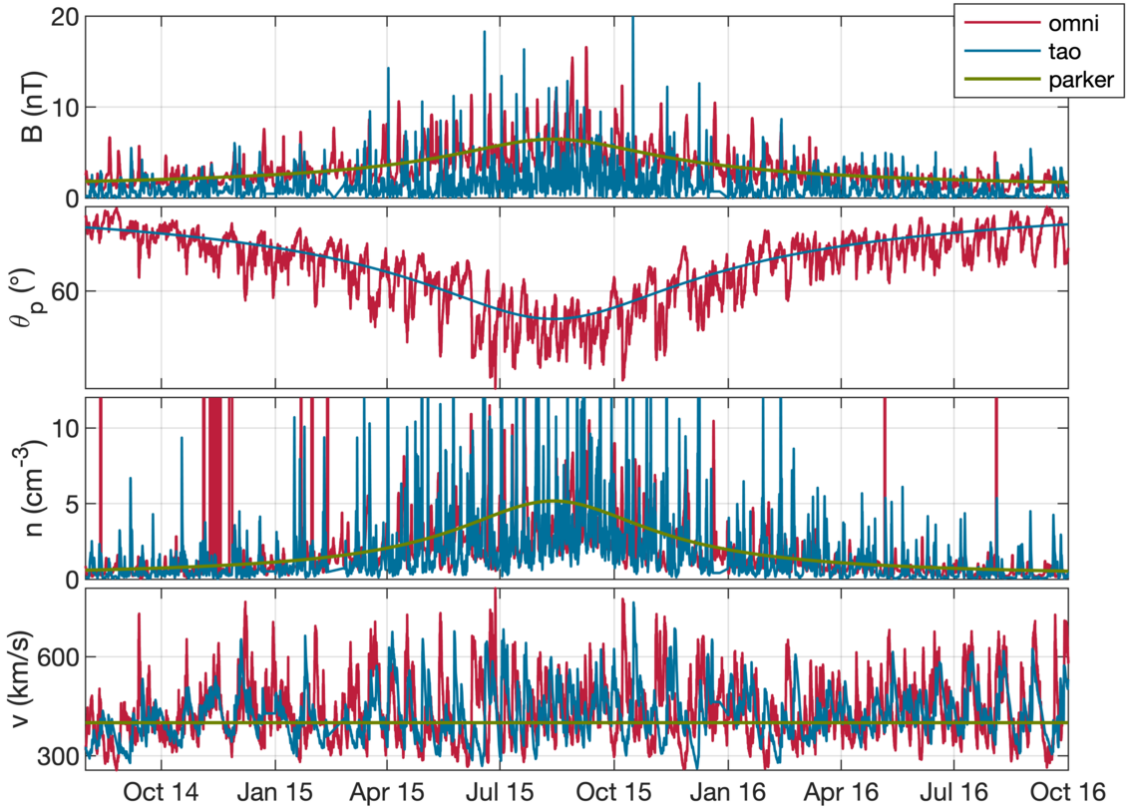
Estimate of the solar wind parameters at 67P during the Rosetta mission. From top to bottom: magnetic field magnitude, Parker angle, density, speed. We show the results of three different propagation models, description see text. From Goetz (2019)

Figure 4 shows estimations of key parameters from different models of the solar wind at the location of the comet. Unfortunately, the Rosetta mission was not equipped with a dedicated upstream solar wind monitor, and after its arrival never left the sphere of influence of the comet, thus no measurements of the undisturbed solar wind are available.

The three models shown here are:

1. a simple Parker model (Parker 1958), based on a fixed set of conditions at Earth: $B=6~\text{nT}$, $v=400~\text{km/s}$ $\theta=45^{\circ}$ and $n=8~\text{cm}^{-3}$. These are then simply extrapolated to the comet’s position,
2. the Tao model (Tao et al. 2005), a 1D MHD model that propagates solar wind measurements made at Earth to the comet. For the magnetic field only the tangential component is computed, so a calculation of the Parker angle, which is the angle between the solar wind velocity and the magnetic field direction, is not possible, and
3. a simple model also based on Earth-based observations of the solar wind (OMNI dataset), that uses the Parker (1958) approach to compute the values at the location of the comet.

**Fig. 5:**
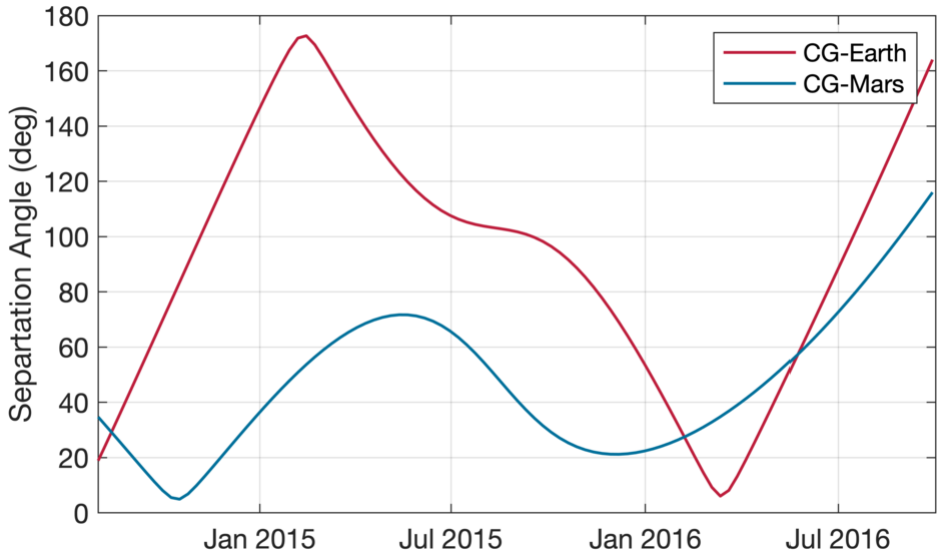
Separation angle between Earth and comet 67P and Mars and 67P for the duration of the comet phase of the Rosetta mission

Other sources are also available, e.g., the ENLIL (Odstrcil 2003) and mSWiM (Zieger and Hansen 2008) models, with similar results as the OMNI and Tao models. An important limitation of the propagation is the angular separation between the source data set and the point at which the solar wind is to be predicted. Here, the separation angle of the two points in the solar system can give an estimate of the goodness of the predicted values. Figure 5 shows this angle for the case Earth-67P and Mars-67P. It becomes clear that for most of the Rosetta mission, Mars is closer to 67P than Earth and therefore e.g. MAVEN and Mars Express data are more suitable as a source for solar wind models. Two exceptions are the periods in August 2014 and in early 2016, where Earth and 67P were almost aligned.

From the propagation models we can derive typical characteristics of the solar wind at 67P. The first panel of Fig. 4 shows the magnetic field. It is clear that the closer the comet comes to the Sun, the higher the field magnitude. In the observation based models, the variability is of the order of $5~\text{nT}$. A clear dependence on the solar rotation period ($\sim 25$ days) is visible as periodic increases in the field. The second panel shows the Parker angle $\theta $, which decreases as the comet approaches the Sun. The lowest values are around $50^{\circ}$, slightly higher than typical Earth values. The third panel shows the density, which increases towards perihelion, as expected. Typical values range between $2\text{--}8~\text{cm}^{-3}$, with increases due to Corotating Interaction Regions (CIRs) or Interplanetary Coronal Mass Ejections (ICMEs) reaching above that range. The velocity of the solar wind is shown in the fourth panel. It does not change depending on the comet’s heliocentric distance. The variation in velocity consists mostly of periodical spikes that are caused by CIRs. The values range between $300~\text{km/s}$ to $800~\text{km/s}$. It becomes clear that transients such as CIRs and ICMEs change the solar wind parameters on short time scales.

CIRs are thought to be emanating from Coronal hole regions on the Sun. They are regions where the fast plasma from the coronal hole interacts with the slow plasma from the surrounding surface. Since the coronal hole rotates with the Sun and thus the origin of the CIR rotates, they appear to be spiralling outward into the solar system. The leading edge of a CIR is often associated with a forward shock, while a reverse shock forms the trailing edge (Pizzo 1985).

Sunspots are not only the source of flares, they also eject a large amount of plasma into the solar system, which is called a coronal mass ejection. These regions of high density, high temperature plasma propagate with a velocity that is usually larger than the average solar wind velocity. If this is higher than the magnetosonic speed in the solar wind, a shock will form in front of the ICME. The structure of the ICME is usually composed of a fast forward shock, compressed and heated plasma from the slow solar wind, ejecta, and a magnetic cloud (e.g. Tsurutani et al. 1988). When moving from upstream to downstream, the shock is recognisable as a steep increase in magnetic field strength, plasma density and temperature as well as a decrease in velocity. The magnetic cloud is often characterised by very stable magnetic fields.

Other, smaller scale phenomena of the solar wind, like e.g. magnetic holes can also impact the plasma around the comet, albeit with smaller scale reactions (Plaschke et al. 2018).

### Cometary Plasma

Cometary ions primarily stem from the ionization (mainly photo-ionization and electron-impact ionization) of neutral species, although, to a lesser extent, they are also produced through charge exchange between solar wind ions and cometary molecules (see e.g. Gombosi 2015). This translates into the continuity equation:

3 $$ \dfrac{\partial n_{j}}{\partial t} + \nabla \cdot ( n_{j}\,{\mathbf{{c}}_{\mathbf{j}}} )= S_{j} - L^{tot}_{j} $$

where $n_{j}$ stands for the ion number density of the species $j$, ${\mathbf{{c}}_{\mathbf{j}}}$ its mean velocity, $S_{j}$ is the sum of the sources for the species $j$, and $L^{tot}_{j}$ the sum of the chemical losses (ion-neutral reactions and dissociative electron-ion recombination). Under steady-state conditions which are usually met, Eq. (3) is reduced to a balance between production and loss processes:

4 $$ S_{j} = \nabla \cdot ( n_{j}\,{\mathbf{c}_{\mathbf{j}}} ) + L^{tot}_{j} $$

Cometary ions primarily stem from the ionization of the neutral species, mainly molecules which compose the coma, although they are also produced through charge exchange between solar wind ions and cometary molecules (Sect. 2.2.1). A given ion species $j$ can also be produced through ion-neutral chemistry (Sect. 2.2.3). The ions are lost through transport as well as ion chemistry, including both ion-neutral reactions and dissociative electron-ion recombination (Sects. 2.2.2 and 2.2.3). The ion composition resulting from the balance between ion sources and ion losses is discussed in Sect. 2.2.3, while the electron density profile (or total ion density profile) is presented in Sect. 2.2.4.

#### Cometary Plasma Sources

Four main mechanisms lead to the formation of cometary ions, counted as a source term in the continuity equation (Eq. (3)):

**Photo-ionisation (PI)**: neutral molecules and atoms absorb sunlight. Below a specific wavelength $\lambda _{\text{th}}$, Extreme Ultra-Violet (EUV) solar photons are sufficiently energetic to strip one electron (or more) from the neutral species $X$ such that:

$$ X+h\nu \overset{\lambda < \lambda _{\text{th}}}{\longrightarrow} Y^{+} + e^{-} $$

Note that in the general case a molecular species $X$ may dissociate into molecular and atomic fragments, resulting in the production of an ion species $Y^{+}$ different from its source neutral (i.e. either $X=Y$ or $X\neq Y$ with $Y$ made of a fraction of the atoms composing $X$). Wavelength thresholds (energy thresholds) for cometary neutral species are typically in the range $85\text{--}105~\text{nm}$ as shown in Table 1.

**Table 1:** Ionisation wavelengths and energies (IE) for a few molecules and atoms of cometary relevance. These thresholds can be found for example in (Avakyan 1998) and at the National Institute of Standards and Technology (NIST, Linstrom and Mallard 2021)

Species	Wavelength [nm]	IE [eV]
H_2_O	98.4	12.6
CO_2_	90.0	13.77
CO	88.5	14.01
O_2_	102.7	12.07
CH_4_	98.4	12.6
NH_3_	123.7	10.02

The major neutral species are not ionised by the strong EUV solar Lyman-$\alpha $ line (121.6 nm), but minor species can be (e.g., sodium). In addition, there are weaker EUV lines which ionise to a lesser extent, such as HeII (30.4 nm).

Newborn photoelectrons contribute to the warm ($\sim 10~\text{eV}$) electron population (see also Sect. 2.3.4 and Broiles et al. 2016a). As the comet comes closer to the Sun and the photo-ionisation rate scales with the inverse square of heliocentric distance, photo-ionisation becomes increasingly more efficient.

However, as the outgassing activity increases, the coma becomes more and more opaque to EUV radiation which prevents photo-ionisation from being effective close to the nucleus: only very energetic photons with short wavelengths ($<40~\text{nm}$) can penetrate. As EUV photons pass through the coma, the neutral column density increases and the EUV flux decreases. EUV solar photons are ‘lost’ and absorbed as a fraction of them have already ionised molecules upstream. This fraction depends on the outgassing rate but also on the wavelength. Bhardwaj (2003) and Beth et al. (2019) showed that photo-absorption (PA) has to be considered for outgassing rates higher than $10^{27}\text{--}10^{28}~\text{s}^{-1}$ for a pure-water coma (which corresponds to conditions a few months around perihelion, see Heritier et al. 2018).

Photoelectron impact ionisation dominates over photo-ionisation in the optically thick part of the coma for large outgassing rates (Bhardwaj 2003). For a planetary atmosphere under hydrostatic equilibrium, the maximum photoelectron production rate occurs at the altitude where the optical depth reaches 1. In contrast, at comets with an expanding coma (*not* under hydrostatic equilibrium), the maximum production of photoelectrons occurs at an optical depth $\tau =2$ (Beth et al. 2019) as the neutral density, Eq. (1), falls off as $1/r^{2}$. For outgassing rates below $\sim 10^{27}~\text{s}^{-1}$, the cometocentric distance at which $\tau =2$ is below the surface nucleus (at least on the dayside) and therefore photo-absorption is negligible. Note that photo-absorption depends on the solar zenith angle and that Rosetta was orbiting most of the time in the terminator plane.

**Electron-impact (EI).** Like photons, energetic free electrons can impact and strip one or more electrons from a molecule. This process takes place for electrons with energies $E>E_{\text{th}}$ such that:

$$ X+e^{-}\overset{E>E_{\text{th}}}{\longrightarrow} Y^{+} + 2e^{-} $$

Most cometary species have electron ionisation thresholds around $12\text{--}14~\text{eV}$ (Itikawa and Mason 2005). Electrons of such energies are less likely to be produced by photo-ionisation, at least at large heliocentric distances ($>2\text{--}3~\text{au}$). Instead, they are likely solar wind electrons ($\sim 10~\text{eV}$) diving towards the cometary nucleus, accelerated up to $50\text{--}60~\text{eV}$ by the ambient ambipolar electric field (see Deca et al. 2017; Divin et al. 2020). EI is a dominant process at large heliocentric distances, whereas PI dominates near perihelion (Bodewits et al. 2016; Heritier et al. 2018).

**Solar wind charge-exchange (SWCX).** At very large cometocentric distances, the plasma is dominated by the solar wind plasma, mainly composed of energetic protons, alpha particles, and electrons, with a small addition of multiply-charged heavy ions. Through charge exchange with the neutral coma, fast, light solar wind $\text{H}^{+}$ and $\text{He}^{2+}$ ions may capture electrons from slow, heavy neutral species (Fuselier et al. 1991; Bodewits et al. 2004; Simon Wedlund et al. 2019):

$$\begin{aligned} &X+ \text{H}^{+\phantom{2}}\phantom{e} \longrightarrow Y^{+} + \text{H}\phantom{e}\phantom{^{+}}\longleftrightarrow Z^{+} + \text{H}^{-}\\ &X+ \text{He}^{2+} \longrightarrow Y^{+} + \text{He}^{+}\longrightarrow Z^{+} + \text{He} \end{aligned}$$

On average, the impacting ion species becomes neutralised. The net result is the creation of slow heavy ions. A cumulative process in nature, this mechanism is at play over tens of thousands to millions of kilometres in the expanding neutral coma; it dominates over photo- and electron-impact ionisation at large cometocentric distances where the mean free path of the solar wind plasma decreases, i.e., the solar wind cannot pass through the coma without colliding with the neutrals at least once (Bodewits et al. 2016). This is of importance when the coma is dense and extended as is the case when, for instance, the comet reaches perihelion. SWCX, although not a producer of net ionisation in the cometary plasma environment, is de facto partaking in the momentum transfer between fast solar wind ions and the slow neutral coma, becoming the main process modulating the formation and extent of the bow shock-like structure upstream of the cometary nucleus for high outgassing activity (Simon Wedlund et al. 2017). During the Rosetta mission, SWCX was not a significant ion source process, except very episodically (Simon Wedlund et al. 2019b, 2020).

**Solar wind impact ionisation (SWI).** Ionisation of the cometary neutrals by direct impact of the fast solar wind ions contributes to the total net production of ions in the coma. For example, for solar wind protons:

$$ X+ \text{H}^{+\phantom{2} } \phantom{e}\longrightarrow Y^{+} + \text{H}^{+} + e^{-} $$

Combi et al. (2004) following Budzien et al. (1994) compared solar wind ionisation frequencies for typical solar wind fluxes at $1~\text{au}$ of $3\times10^{8}~\text{cm}^{-2}\,\text{s}^{-1}$. They emphasised that SWCX should dominate over SWI by at least a factor 7 in efficiency in these nominal conditions. For 67P, Simon Wedlund et al. (2019b) reported SWI frequencies about $1\text{--}7\%$ ($2\%$ average) of those of SWCX, except at times when faster solar wind streams were recorded, possibly of CME/CIR origin, where SWI cross sections start to become dominant over SWCX cross sections. The produced secondary electrons, called proto-electrons, can be energetic enough to trigger more ionisation in the neutral coma. However, because SWI is a minor contributor to the total ionisation, ionisation by energetic proto-electrons is expected to be small on average and may only play a role when SWI itself becomes non-negligible (e.g. Simon Wedlund et al. 2019, 2020, for a high-speed solar wind for which SWI cross sections are favoured over SWCX).

**Fig. 6:**
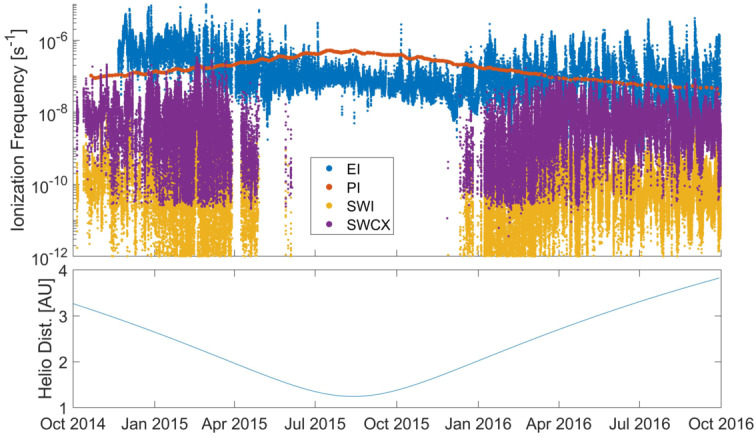
(Top) Ionisation frequencies of water from electron impact (EI, blue), photo-ionisation (PI, red), Solar wind ionisation (SWI, yellow) and solar wind charge exchange (SWCX, purple) at the Rosetta spacecraft (see also Simon Wedlund et al. 2019b, 2020). The ionisation frequency of PI and EI may not represent the ionisation rate throughout the coma, especially near perihelion when electron degradation is significant and the optical depth becomes large. SWCX and SWI are calculated only when Rosetta was outside of the solar wind ion cavity as defined by Behar et al. (2017). (Bottom) Heliocentric distance of 67P throughout the Rosetta mission

Depending on the heliocentric distance, the cometocentric distance and the solar wind conditions, the relative efficiency of these mechanisms varies (Heritier et al. 2018; Simon Wedlund et al. 2019b, 2020), and, unlike the ion-neutral chemistry discussed in Sect. 2.2.3, these mechanisms are net sources of plasma. Figure 6 displays the 15-min averaged ionisation frequency of all the separate ionisation channels (PI, EI, SWCX and SWI) as calculated by Simon Wedlund et al. (2019b, 2020) and based on the latest EI data. The EI frequencies are derived from the electron flux (outlined by Stephenson et al. 2021), with a 2- to 15-min temporal resolution, and ionisation cross sections from Itikawa and Mason (2005). Roughly speaking, when still outside of the solar wind ion cavity (from October 2014–April 2015 and December 2015 to end of mission, see Fig. 6), EI dominated most of the time, except between January and April 2016 when photo-ionization was likely dominating (although as shown by Heritier et al. 2018, only a few EI frequencies could be derived then). In periods, most notably at the end of the mission, SWCX frequencies may have become almost as large as EI and PI frequencies.

It is worth mentioning that the solar activity varied during the Rosetta escort phase. It started in the middle of the Solar cycle 24, a few months after the number of sunspots peaked. From then on, the solar activity monotonically decreased, causing an asymmetry in photo-ionisation between inbound and outbound periods around perihelion (Heritier et al. 2018).

#### Cometary Ion Losses

The main process causing a net local loss of plasma is electron-ion dissociative recombination (DR). If the plasma number density is sufficiently high and electrons are cold, ions and electrons merge back together. However, the excess energy during this process induces a fragmentation of the molecule, hence the term “dissociative” (e.g. $\text{H}_{2}\text{O}^{+} + e^{-} \rightarrow \text{HO} + \text{H}$). The associated loss term $L_{\text{R},j}$ for ion $j$ of density $n_{j}$, expressed in $\text{s}^{-1}$ is given by:

5 $$ L_{\text{R},j}=\alpha _{j}(T_{e})\ n_{j}\ n_{e}=\alpha _{j}(T_{e})\ n_{j} n_{e} $$

where $n_{e}$ is the electron number density (in $\text{m}^{-3}$), $\alpha_{j}(T_{e})$ is the DR coefficient, expressed in $\text{m}^{6}\,\text{s}^{-1}$, depending on the species $j$ and on the electron temperature $T_{e}$. As $T_{e}$ decreases, $\alpha _{j}(T_{e})$ increases. The DR rate constant is thus simply $k_{ei} = \alpha _{j}(T_{e})\,n_{e}$ (in $\text{m}^{3}\,\text{s}^{-1}$) (Heritier et al. 2017a).

A competing process is the divergence of the flux. The transport term $\nabla \cdot ( n_{j} {{\mathbf{c}}_{j}} )$ represents the rate at which ions $j$ are coming and leaving a specific location in space. In the case of dense planetary atmospheres and ionospheres, this transport is mainly “vertical” and due to the diffusion of the ions upward or downward, and their chaotic motion through the neutrals or other ions of different masses (so-called *eddy* and *molecular diffusions*). At comets, the plane-parallel approximation (i.e., assuming that the curvature of the atmosphere can be neglected) is not applicable and ions are born from neutrals with a non-negligible speed (from $\sim 300~\text{m}{\cdot }\text{s}^{-1}$ at the surface to $\sim 900~\text{m}{\cdot }\text{s}^{-1}$ from a few tens of kilometres because of the acceleration and adiabatic expansion of the neutral gas, see Heritier et al. 2017b). In addition, a substantial effect which is negligible at large Solar System bodies is the symmetry of the outgassing flow (spherical, propagating from a point source). Indeed, assuming spherical symmetry for the neutral coma and ionosphere in the first few hundreds of kilometres from the nucleus, the transport term becomes:

6 $$ \nabla \cdot ( n_{j}\ {\vec{c}_{j}} )\approx \dfrac{1}{r^{2}} \dfrac{\mathrm{d}}{\mathrm{d}r}\left [n_{j}(r)\ c_{j}(r)r^{2}\right ]= \dfrac{\mathrm{d}}{\mathrm{d}r}\left [n_{j}(r)\ c_{j}(r)\right ]+ \dfrac{2n_{j}(r)\ c_{j}(r) }{r}>0 $$

where $c_{j}(r)$ stands for the purely radial ion velocity. The first term on the right-hand side is equivalent to an ion flow in the plane-parallel approximation (substituting $r$ with $z$, the altitude). The second term is due to the local curvature: as we move farther away, its effect decreases, although our assumption (spherical symmetry) may not hold anymore.

#### Ion Chemistry

Following the previous sections on ion sources and losses, the steady-state continuity equation, Eq. (4), for an ion species $j$ of density $n_{j}$ reduces in a spherically symmetric coma to (e.g., see Heritier et al. 2017a):

7 $$\begin{aligned} \underbrace{\dfrac{1}{r^{2}}\dfrac{\mathrm{d}}{\mathrm{d}r}\left (n_{j}(r)\ c_{j}(r)\ r^{2}\right )}_{ \text{transport}} = \underbrace{\vphantom{\dfrac{1}{1}}I_{j}(r)}_{ \text{ionisation}}\ +\ \underbrace{\vphantom{\dfrac{1}{1}} P_{j}(r)\ - L_{j}(r)}_{ \text{ion-neutral chemistry}} -\ \underbrace{\vphantom{\dfrac{1}{1}} L_{R,j}(r)}_{\text{DR}} \end{aligned}$$

where:

- $I_{j}=\sum _{p} \nu _{p}(r)\ n_{p}(r)$ is the production of an ion $j$ through ionisation (PI, EI, SWI) of, or charge exchange (SWCX) with, a parent neutral species $p$ of density $n_{p}$ (in $\text{m}^{-3}$). $\nu _{p}$ (in $\text{s}^{-1}$) is the ionisation rate of process $p$ and depends on the cometocentric distance $r$ as discussed in Sect. 2.2.1,
- $P_{j}(r)=\sum _{s,i} k_{s+i\rightarrow j }\ n_{s}(r)\ n_{i}(r)$ stands for the ion-neutral production reaction between the neutral species $s$ and the ion species $i$, yielding the ion species $j$ and neutral products. For instance, in the case of $j=\text{NH}_{4}^{+}$, produced through proton-transfer from $\text{H}_{3}\text{O}^{+}$ (protonated H_2_O),
  $$ \text{NH}_{3} + \text{H}_{3}\text{O}^{+}\longrightarrow \text{NH}_{4}^{+}+ \text{H}_{2}\text{O} $$
- $L_{j}(r) = n_{j}(r) \sum _{s^{\prime},i^{\prime}} k_{s^{\prime}+j \rightarrow i^{\prime}}\ n_{s^{\prime}}(r)$ represents the ion-neutral loss reaction between the neutral species $s^{\prime}$ and the ion species $j$, yielding the ion species $i^{\prime}$ and neutral products. For instance, $\text{H}_{2}\text{O}^{+}$ (protonated OH) is lost through proton-transfer with H_2_O (protonated OH),
  $$ \text{H}_{2}\text{O} + \text{H}_{2}\text{O}^{+}\longrightarrow \text{H}_{3} \text{O}^{+}+ \text{HO} $$
  This term also includes charge exchange processes between ions and neutrals except symmetric reactions such as $\text{H}_{2}\text{O} + \text{H}_{2}\text{O}^{+}\longrightarrow \text{H}_{2} \text{O}^{+} + \text{H}_{2}\text{O}$. This does not affect the plasma density, but the momentum equation and higher moments. For instance, this reaction is more efficient than proton transfer at energies above 1 eV (Lishawa et al. 1990) (see also Sect. 4.5).
- $L_{R,j}$ corresponds to the loss of the ion species $j$ through electron-ion dissociative recombination (DR, see Sect. 2.2.2).

The ion-neutral kinetic reaction rate constants $k_{s+i\rightarrow j}$ and $k_{s^{\prime}+i\rightarrow j}$, expressed in $\text{m}\,\text{s}^{-1}$ for bimolecular collisions, are a function of the gas (neutral) temperature or a combination of ion and neutral temperatures (Banks and Kockarts 1973; Rees 1989; Anicich 1993), which are difficult to constrain through observations (Biver et al. 2019; Myllys et al. 2019; Wattieaux et al. 2020, and Sect. 3.1.3). The reaction temperature is sometimes assumed constant for lack of better constraints.

**Composition.** In theory, due to the complexity and diversity of the neutral coma, the same degree of complexity is expected for the ion environment. Historically, the first identified ion species at a comet was $\text{CO}^{+}$ as part of the bright blue “Comet-Tail emission” (Fowler 1910; Larsson et al. 2012). However, only in-situ detections may help unravel the greater extent of the ion composition. The first mission to perform in-situ analysis of the ion composition was ESA’s Giotto mission to comet 1P/Halley in 1985 (Reinhard 1986). Most spectrometers on board had a mass resolution of about $\Delta m \approx 1~\text{u}\,\text{q}^{-1}$ which allowed separation of molecules with a different number of nucleons (e.g., 18 for $\text{H}_{2}\text{O}^{+}$ and 19 for $\text{H}_{3}\text{O}^{+}$) (Balsiger et al. 1986). However, like neutrals, different ion species may have the same number of nucleons (e.g., $\text{H}_{2}\text{O}^{+}$ and $\text{NH}_{4}^{+}$), which means that these ions are indistinguishable. Aided by photochemical models, constraints were provided for the ion composition and the relative contribution of ions at a given $\text{u}\,\text{q}^{-1}$ could be estimated (Altwegg et al. 1993; Rubin et al. 2009).

As with many aspects of cometary physics, the Rosetta mission to comet 67P was the first to systematically survey with very high mass and/or temporal resolutions the ion composition of the cometary coma. This was achieved by a combination of three ion analysers and spectrometers on board Rosetta, namely the Rosetta Plasma Consortium Ion Composition Analyser (RPC-ICA, Nilsson et al. 2007), the RPC Ion and Electron Sensor (RPC-IES, Burch et al. 2007), and the double focusing magnetic mass spectrometer (ROSINA/DFMS) (Balsiger et al. 2007).

In practice, the plasma environment surrounding 67P was found to consist in general of two distinct populations of ions, ions of cometary origin and ions of solar-wind origin. Their presence and distribution depended on the interplay between sources and losses:

**Cometary ions** (**above** $12~\text{u}\,\text{q}^{-1}$). Sources and losses of ions due to the physico-chemistry between EUV photons, energetic electrons, solar wind ions and neutrals within the coma result in the presence of stable cometary ions. Water-group ions (masses around $18~\text{u}\,\text{q}^{-1}$) were first detected by RPC-ICA during the approach phase (Nilsson et al. 2015a). However, RPC-ICA’s inherent mass resolution was insufficient to separate ions with close mass-per-charge ratios (e.g., $\text{H}_{2}\text{O}^{+}$ from $\text{H}_{3}\text{O}^{+}$). Using ROSINA/DFMS in high-resolution (HR) mode, several ion species were unambiguously identified near perihelion, such as $\text{H}_{2}\text{O}^{+}$, $\text{H}_{3}\text{O}^{+}$, and $\text{NH}_{4}^{+}$ (Fuselier et al. 2016; Beth et al. 2016). However, this trio of species only formed the tip of the iceberg. They were identified at the most favourable periods, when the outgassing rate was the highest. Fuselier et al. (2015) showed that in December 2014 (low activity), many other ions were also present although their study relied on the low resolution (LR) mode of the instrument, more sensitive than the HR mode at the expense of a lower mass resolution (e.g., no separation of $\text{H}_{2}\text{O}^{+}$ from $\text{NH}_{4}^{+}$).

**Fig. 7:**
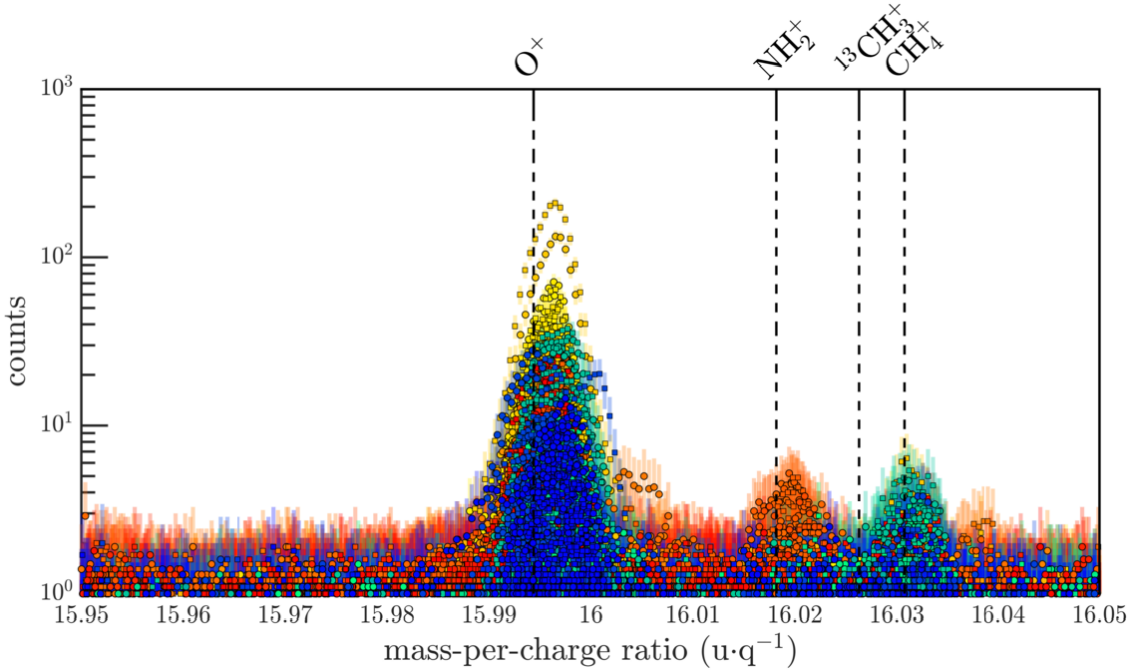
Overview of spectra at $16~\text{u}\,\text{q}^{-1}$ over the whole mission in high resolution mode. Colours depend on the period during the escort phase. The detected ion species are $\text{O}^{+}$, $\text{NH}_{2}^{+}$, and $\text{CH}_{4}^{+}$. From Beth et al. (2020), reproduced with permission ©ESO

Recently, Beth et al. (2020) analysed and reviewed the full dataset acquired by ROSINA-DFMS in ion mode with both resolutions between Oct 2014 and April 2016. Besides $\text{H}_{2}\text{O}^{+}$, $\text{H}_{3}\text{O}^{+}$, and $\text{NH}_{4}^{+}$, more than 20 ions were identified in situ in 67P’s ionosphere (see Fig. 7 for an example at mass 16 u). Minor cometary ion species that had been either previously predicted (such as $\text{CH}_{3}\text{OH}_{2}^{+}$) or observed remotely (such as $\text{CO}^{+}$) were unambiguously identified from 13 to $39~\text{u}\,\text{q}^{-1}$. However, the in-situ detection of their presence in cometary ionospheres such as that of 1P/Halley previously relied on the combination of both observations in LR and photo-chemicals models. In contrast, ROSINA-DFMS made it possible to rely on HR observations alone to exactly identify ions with a precision of $<0.01~\text{u}\,\text{q}^{-1}$ (see Fig. 7).

Above $40~\text{u}\,\text{q}^{-1}$, ROSINA-DFMS sensitivity falls drastically and no ions were identified in HR. In LR ($m/\Delta m\sim 500$), peaks were observed at each integer mass-to-charge ratio up to $142~\text{u}\,\text{q}^{-1}$. In particular, the large peak at mass $44~\text{u}\,\text{q}^{-1}$ contains $\text{CO}_{2}^{+}$; however, this mass can be populated by other ions (e.g., $\text{C}_{3}\text{H}_{8}^{+}$), and therefore it is difficult, without the use of photo-chemical models, to gauge its contribution throughout the escort phase. ROSINA/DFMS operated either in neutral *or* in ion mode, preventing simultaneous neutral and ion measurements.

**Fig. 8:**
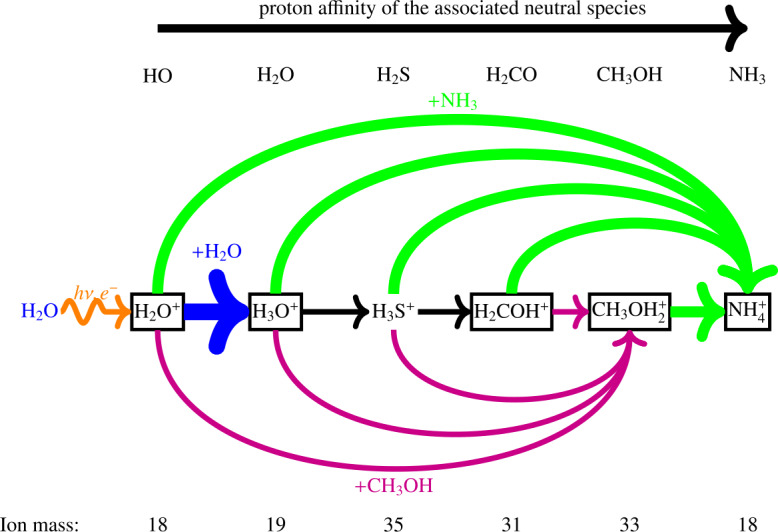
Schematic of the role of species with high proton affinity molecules on the ion composition in the coma. Arrows represent ion-neutral reactions, from the reactant to the product. Framed ions have been detected by ROSINA/DFMS (Beth et al. 2020). Only H_2_O does not result from protonation (of HO)

Cometary ions can be separated approximately into three main families (Beth et al. 2020). They can be produced by ionisation of a parent molecule and lost through transport, lost through ion-neutral chemistry with H_2_O, or only produced by chemistry (such as protonated molecules, see Fig. 8) and lost either through transport or chemistry.

Finally, the detection of a *dication* (doubly-charged ion), $\text{CO}_{2}^{2+}$, was reported for the first time at a comet (Beth et al. 2020). A large peak was observed at $22~\text{u}\,\text{q}^{-1}$ in low resolution above the Southern Hemisphere in the early phase of the mission, at large heliocentric distances, where $\text{CO}_{2}$ dominated the neutral composition. $\text{CO}_{2}^{2+}$ had long been observed in laboratory experiments (Mathur et al. 1995) and predicted with kinetic transport models in the CO_2_-rich atmospheres of Mars and Venus (Witasse et al. 2002; Gronoff et al. 2007; Lilensten et al. 2013).

**Solar wind-derived ions** (**mainly at** 1, 2, **and** $4~\text{u}\,\text{q}^{-1}$). Light solar wind ions ($\text{H}^{+}$, $\text{He}^{2+}$, and $\text{He}^{+}$ through SWCX) constitute the bulk and were routinely detected by RPC-ICA throughout the mission (Nilsson et al. 2015a,b; Simon Wedlund et al. 2016, 2019b) until the solar wind ion cavity formed and solar wind ions were prevented from penetrating deeper into the coma at the location of Rosetta (Behar et al. 2016b). Hydrogen anions $\text{H}^{-}$ were reported in the RPC-IES data at the beginning of the mission up to January 2015 (Burch et al. 2015a). Heavy multiply-charged ions of solar wind origin (e.g., $\text{O}^{n+}$, $\text{C}^{n+}$, $\text{Ne}^{n+}$, $\text{Mg}^{n+}$, $\text{Fe}^{n+}$, $n\geq 3$, and their charge-exchanged products) are also expected, although they have not been evidenced at 67P. Their presence can be inferred indirectly from the detection of X-ray emissions, as in the case of comet C/1996 B2 (Hyakutake) whose X-ray coma, first observed in 1996, heralded a new era in astrophysical and cometary X-ray astronomy (e.g., Lisse et al. 1996; Cravens 1997; Lisse et al. 2004; Bodewits et al. 2007, see also Sect. 2.5). For a longer description of solar wind plasma at 67P, as well as pick-up (heavy) cometary ions, see Sect. 2.3.1.

**Cometary ion variations during the Rosetta mission.** In addition to listing in-situ cometary ions, Beth et al. (2020) also investigated their variability. Some ions were detected at large heliocentric distances but not near perihelion and, conversely (see Fig. 7 and different colours for different periods of the Rosetta mission). This was ascribed to the collisionality and reactivity of ions with water. On the one hand, at large heliocentric distances, the detected ions are those produced from direct ionisation of a parent molecule. Even if they react with water, they will not be chemically lost at the location of Rosetta through ion-neutral reactions, mainly with H_2_O, CO_2_, and H_2_CO. On the other hand, near perihelion, mainly two kinds of ions were detected: those not reacting with water (e.g., $\text{CH}_{3}^{+}$), and ‘protonated molecules’ (e.g., $\text{CH}_{3}\text{OH}_{2}^{+}$, $\text{NH}_{4}^{+}$) yielded by ion-neutral reactions between water ions (either $\text{H}_{2}\text{O}^{+}$ or $\text{H}_{3}\text{O}^{+}$) and cometary molecules with a high proton affinity (e.g., CH_3_OH and NH_3_, see Fig. 8). Only a few neutral cometary molecules may react with $\text{H}_{2}\text{O}^{+}$ or $\text{H}_{3}\text{O}^{+}$ by ‘stealing’ a proton from them and become *protonated* molecules. Figure 8 shows how species with high proton affinity affect the coma composition. Photochemical models dedicated to 67P’s ionosphere and driven by DFMS observations include for example that of Heritier et al. (2017a) in the mass range 28–37 u around perihelion.

In contrast to previous fly-by missions (e.g., Curdt et al. 1988, for Giotto), which mainly explored the outer coma and the inner coma to a lesser extent, Rosetta was orbiting 67P in the inner coma. For instance, Balsiger et al. (1986) showed that the plasma number density exhibit a $1/r$ trend close to the comet, whereas farther away, the density followed a $1/r^{2}$ dependence. As far as is known, only the $1/r$ trend was observed and reported at Rosetta (Edberg et al. 2015, for example). In addition, the protonated molecules tend to be present in (and dominate) the ion composition of the inner coma (Heritier et al. 2017a) where ion-neutral reactions are favoured. As one moves away from the nucleus, the protonated molecules fade away whereas $\text{H}_{2}\text{O}^{+}$ is still replenished by ionisation of the coma and dominates the ion composition (see for example Beth et al. 2019, and their simple model for $\text{H}_{2}\text{O}^{+}$ and $\text{H}_{3}\text{O}^{+}$).

A list of possible ion-neutral and dissociative electron-ion recombination reactions for cometary ions is given in Appendix B of Heritier et al. (2017a) for comet 67P, and in Häberli et al. (1995) and Rubin et al. (2009) for 1P/Halley; the reader is also directed towards dedicated databases, as described in Sect. 3.1.3.

#### Plasma Balance

In this section, we explore the consequences of the equation of continuity, assuming both spherical symmetry, a negligible magnetic field, and the absence of all electric fields. For a description of the electric fields see Sect. 4.1, and for a plasma balance model taking the ambipolar field into account see Sect. 2.3.2.

Because of the quasi-neutrality of a plasma, the number density of electrons $n_{e}$ can be described by summing the number density of ions over all ion species (total ion density, $n_{i}$). If we sum the continuity equation, Eq. (7), over each ion species $j$, we get:

8 $$\begin{aligned} \dfrac{1}{r^{2}}\dfrac{\mathrm{d}}{\mathrm{d}r}\left (n_{i}(r)\ \overline{u_{\text{ion}}}(r)\ r^{2}\right ) = I(r)\ - L_{R}(r) \end{aligned}$$

where:

- $I(r)=\sum _{p} \nu (r)\ n_{p}(r) = \nu (r) n(r) $ stands for the production of ions through ionisation (primarily, PI and EI) and through charge exchange (SWCX) summed over all parent neutral species $p$ (see Sect. 2.2.1).
- $\overline{u_{\text{ion}}}(r)= \dfrac{ \sum _{j}n_{j}(r)c_{j}(r)}{\sum _{j}n_{j}(r)}$ is the weighted average ion velocity in terms of ion number density of each species $j$.
- $L_{R} = \sum _{j} L_{R,j}$ corresponds to the net charge loss through electron-ion dissociative recombination (DR) (see Sect. 2.2.2).

Note that the ion-neutral chemical source and loss terms ($P_{j}$ and $L_{j}$, see Eq. (7)) cancel out as we sum over all ion species as each (singly-charged) ion created corresponds to one (singly-charged) ion lost.

**At large heliocentric distances** ($>2~\text{AU}$). Under low outgassing conditions, the chemical loss of cometary plasma through dissociative recombination is negligible. For assessing the ion density at cometocentric distances between 10 km and 50–80 km, the ion acceleration is roughly constant and is reduced to an ion average velocity ($\overline{u_{\text{ion}}}(r) = u_{ion}$). Under these conditions, Eq. (8) is reduced to (Galand et al. 2016):

9 $$ n_{i}(r) = \frac{\nu _{0} n(r) (r - r_{c})}{\overline{u_{ion}}}, $$

where $r$ represents cometocentric distance, $n(r)$ is the density of the neutrals, $\nu _{0}$ the total ionisation rate, and $r_{c}$ is the radius of the nucleus.

Equation (9) has been used to organise multi-instrument datasets from Rosetta and to confirm the main cometary plasma sources and plasma balance in the coma of comet 67P at large heliocentric distances (see Sect. 3.2.1). By inspecting Eq. (9) as a function of $r$, one can notice that the ion density is proportional to $n(r)(r-r_{c})$. Assuming a Haser model, Eq. (1), where we neglect the depletion of neutral species through photo-destruction and ionisation, which is a reasonable assumption at low cometocentric distances (a few 1000 km) as the loss processes do not affect the neutral density significantly (Heritier 2018).

The total ion number density (or electron density) can therefore be further approximated to:

10 $$ n_{i}(r) = \frac{\nu _{0} (r - r_{c}) Q_{0}}{\overline{u_{ion}} \overline{u_{n}} r^{2}} $$

where $Q_{0}$ is the total outgassing rate ($\text{s}^{-1}$) and $\overline{u_{n}}$, the neutral radial velocity, both being averaged over the different neutral species for simplicity.

Assuming that the neutral and ion velocities are independent of $r$ at close cometocentric distances ($r<100~\text{km}$), the total ion density becomes solely proportional to $(r-r_{c})/r^{2}$. It is zero at the nucleus surface $r=r_{c}$, and for large $r$, tends towards zero with a $r^{-1}$ slope. While being a much sparser population, the ion density decreases less sharply than the neutral density (proportional to $r^{-2}$) as ions are produced constantly during the coma expansion, while major neutral species only originate from the cometary nucleus. Furthermore, unlike the ion production rates which increase down to the surface with decreasing cometocentric distances (at these low outgassing rates at the heliocentric distances considered here), the electron density exhibits a peak. The $(r-r_{c})/r^{2}$ dependency implies that the peak in total ion density (or electron density) occurs for $r=2r_{c}$ (Heritier et al. 2017b).

**Fig. 9:**
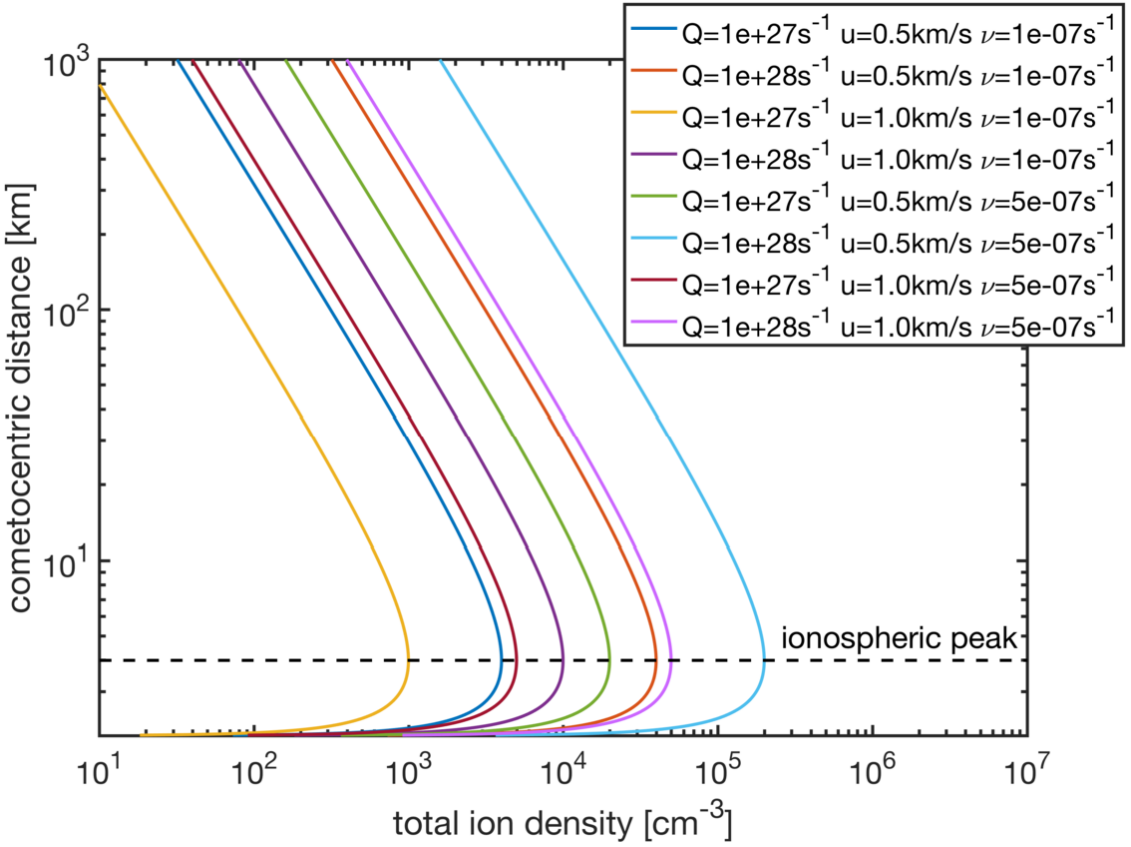
Total ion density as a function of the cometocentric distance computed with Eq. 10 for different neutral outgassing rates, outflow bulk velocities (ion and neutral) and (total) ionization frequencies (from Heritier 2018)

This global maximum can be illustrated in Fig. 9, where we have plotted the ion density as a function of $r$, using Eq. (10) and a set of different outgassing rates, ion radial velocities (in this example also equal to neutral radial velocities), and ionisation frequencies. We can see that for every case, the peak of total ion density occurs at the same $r=2r_{c}=4~\text{km}$. Under real conditions however, the nucleus is not spherical, and we therefore do not expect the ionospheric peak to be always observed at a sharp $r=2r_{c}$. However, this simplified analysis gives a satisfying theoretical result which solely depends on the nucleus theoretical radius and falls very close to what has been observed with in-situ measurements in the case of comet 67P, during the final descent of the Rosetta spacecraft towards the nucleus surface (Heritier et al. 2017b). Indeed, we can observe an ionospheric density peak at $r =5~\text{km}$ (see Fig. 2 in Heritier et al. 2017b). Which is close to the theoretical value of 4 km. The $r^{-1}$ trend as $r$ increases could also be clearly identify in this dataset, even though one must bear in mind that the trajectory of the spacecraft was not radial, and the outgassing rate, ionisation frequencies, and outflow velocities were varying along the trajectory (Heritier et al. 2017b). We therefore do not expect an ion profile close to what is illustrated in the theoretical Fig. 9.

**Near perihelion.** DR and PA should be considered. Indeed, on the one hand, DR depends on the electron density which increases as a function of photoionisation. On the other hand, PA depends on the neutral column density which increases with decreasing heliocentric distances. These two processes compete and decrease the ion number density. Under some assumptions, the ion number density can be analytically solved for each case either ionisation damped by PA or loss though DR but not together. For instance, in the case of no PA, assuming the DR coefficient $\alpha $ as constant and that the ion and neutral velocities are equal, $c_{j} = U_{n}$, although that condition rarely holds at comet 67P due to the presence of electric fields (see Sects. 2.3.2 and 4.1), the continuity equation, Eq. (7), with DR, ionisation and transport only becomes:

11 $$ \dfrac{1}{r^{2}}\dfrac{\mathrm{d}n_{i}(r)U_{n} r^{2}}{\mathrm{d} r}= \dfrac{\nu _{0} Q}{4\pi U_{n} r^{2}} - \alpha n_{i}(r)^{2} $$

and the cumbersome solution is given by Beth et al. (2019):

12 $$ n_{i}(r)=(\gamma -1)\dfrac{U_{n}}{2\alpha r} \underbrace{\dfrac{R^{\gamma}-1}{R^{\gamma}+\dfrac{\gamma -1}{\gamma +1}}}_{> 1-1/R \text{ and } < 1} $$

where $\gamma =\sqrt{1+\tfrac{Q}{Q_{0}}}$, $Q_{0}=\tfrac{\pi U_{n}^{3}}{\nu _{0} \alpha}$, and $R=r/r_{c}$, $r$ being the cometocentric distance and $r_{c}$ the cometary radius. The last term of Eq. (12) is a correction for finite distances: 0 at the surface and increases continuously towards 1 for increasing $R$ such that, for $R\gg 1$, the formula is Eq. (12) from Gombosi (2015). In addition, if $Q\gg Q_{0}$ or $\gamma \approx \sqrt{Q/Q_{0}}\gg 1$ (which is achieved at 1P/Halley while probably not at 67P), the ion number density is that given by Cravens (1987) under photo-chemical equilibrium, i.e. ions are produced and readily destroyed through DR without the time to be radially transported.

This simple mathematical approach reveals that the ion number density profile, whether transport or DR dominates, asymptotically follows $1/r$. As the comet gets closer to the Sun, $\gamma $ drastically increases with the combined increases of $Q$ and $\nu _{0}$. However, this shows that, depending on the equilibrium at play in the inner coma, the dependence of the ion number density with respect to the outgassing rate is different. For $Q\ll Q_{0}$, $n_{i} \propto Q$ while for $Q\gg Q_{0}$, $n_{i}\propto \sqrt{Q}$. Moreover, as $Q_{0}$ is increasing with the electron temperature, e.g. the condition $Q\gg Q_{0}$ is fulfilled for very cold electrons even at perihelion at least for 67P. For instance:

$$ Q_{0}\approx 7.39\,U_{n}[\text{km}{\cdot}\text{s}^{-1}]\,r^{2}_{ \text{67P}}[\text{AU}^{2}]\,\sqrt{k_{B}T_{e}[K]}\times 10^{28}~\text{s}^{-1} $$

such that at 67P’s perihelion, $Q\gtrsim Q_{0}$ only if $k_{B} T_{e}\lesssim 0.1~\text{eV}$. In fact, if one assumes the electron temperature to be that of neutrals (i.e. roughly 100 K), one finds that $Q\approx 4Q_{0}$. Therefore, at 67P, the condition $Q\gg Q_{0}$ does not hold at anytime and the applicability of the Cravens (1987) model at 67P remains arguable.

### Plasma Processes

#### Kinetic Ion Effects

**Fig. 10:**
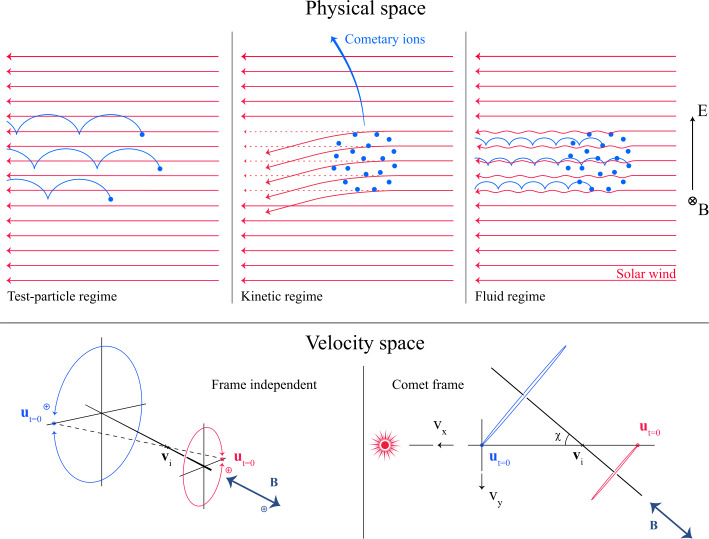
Upper row: illustration of three different regimes for the ion dynamics in physical space (from Behar 2018). Lower row: 3-dimensional evolution of two ion beams given by the generalised gyromotion in velocity space (from Behar et al. 2018b, Fig. 1)

Before the Rosetta era, it was mostly the slow, new-born cometary ions, or pick-up ions, that were considered to showcase kinetic behaviours not captured by a fluid description, while such a fluid description was deemed sufficient and less computationally expensive to describe the solar wind flow within the cometary atmosphere. The foremost kinetic phenomenon at comets is the gyration (Coates 2004): the cometary ions, considered for simplicity as test-particles, are created in a reference frame in which the acceleration resulting from the electric field in combination with the magnetic field results in their movement along cycloidal trajectories, with a characteristic length given by their gyroradius:

13 $$ r_{gi}= \left |\frac{m_{i} v_{i}}{q B}\right |, $$

where $m_{i}$ is the mass of the ion, $v_{i}$ and $q$ its velocity, and charge. $B$ is the magnetic field magnitude. This gyroradius for active comets fairly close to the Sun (such as the one visited by all previous cometary missions) is typically much smaller than the interaction region between the solar wind and the ionised cometary atmosphere. The cometary ions gyrate several times within the interaction region, as illustrated in the fluid regime panel of Fig. 10.

The general picture of the ion dynamics in a cometary environment was widely broadened by Rosetta and the great coverage in parameter space (mostly heliocentric distance and production rate) it allowed. In particular, further away from the Sun, the ordering of these characteristic lengths is reversed and we have to consider the situation where the cometary ion gyroradius is significantly larger than the small, dense ionised cometary atmosphere, as illustrated for this kinetic regime in Fig. 10, upper row. The main difference from the fluid regime to the kinetic regime in this figure is the breaking of the symmetry with regard to the upstream flow direction. While in the fluid description the cometary ions – though gyrating – are on average flowing along the incident solar wind flow direction because of their small gyroradius, they are now seen to escape the system sideways in the kinetic description, along a direction indicated by the upstream electric field, perpendicular to the incident flow. In order to balance the total momentum of the system, the solar wind flow is illustrated as being deflected towards the opposite direction.

**Fig. 11:**
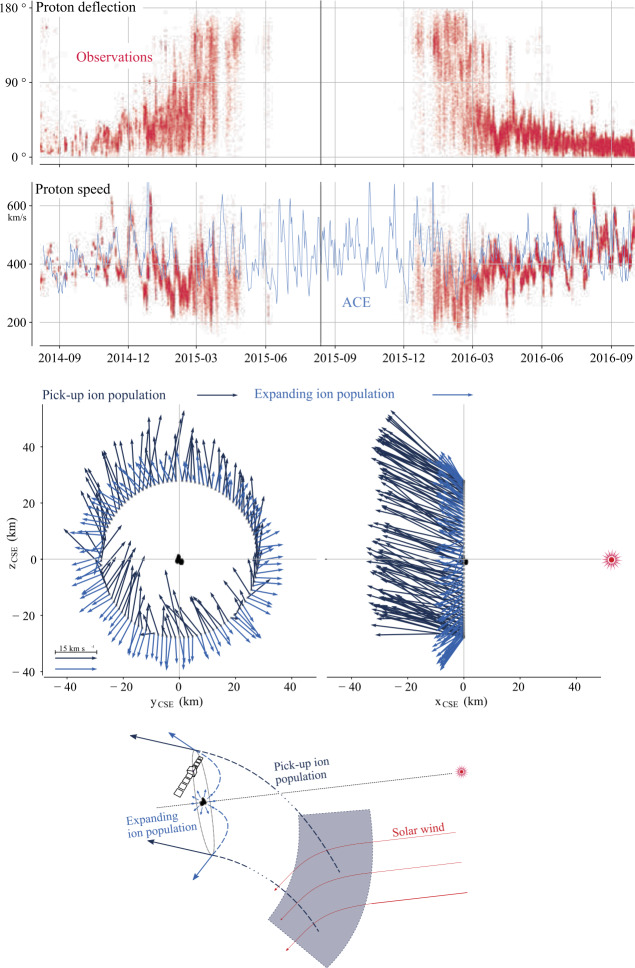
First and second rows: Observations of the deflection angle and bulk speed of the solar wind protons (adapted from Behar 2018; Behar et al. 2017, Fig. 1). Third row: Observations of bulk velocities of two cometary ion populations. Last row, schematics of the acceleration of cometary ions by an already deflected solar wind ion flow. Third and last taken form Berčič et al. (2018), reproduced with permission ©ESO

One can give a more thorough, analytical description of these dynamics. Under most conditions, the electric field is dominated by its motional or convective term (see Sect. 2.3.2). Both ion populations, simplified to cold beams of different velocities, are actually gyrating around their common centre of mass in velocity space, as shown in the second row of Fig. 10: both populations gyrate, and the ratio of their gyroradii is directly linked to the ratio of their masses, as described by Behar et al. (2018a,b). For low-to-medium outgassing activity and in the comet frame, far enough from the dense inner coma, the cometary ions are initially seen accelerated “upward” (along the convective electric field), and the solar wind is seen deflected “downward” (against the convective electric field). An additional, somewhat counter-intuitive characteristic of the dynamics is the limited loss of kinetic energy by the solar wind ions. This early solar wind deflection has been studied extensively, both numerically and experimentally (Bagdonat and Motschmann 2002; Nilsson et al. 2015a; Broiles et al. 2015; Behar et al. 2016a,b), and one main result is reported in Fig. 11. From a limited deflection far away from the Sun, solar wind ions were seen flowing at angles up to 180 degrees from their upstream direction when closer to the Sun, while little loss of kinetic energy was measured (their upstream velocity can be estimated using solar wind measurement propagated from Earth or Mars). Around perihelion, solar wind ions were not observed anymore for a duration of about three months.

We have seen that in the region dominated by the solar wind, cometary ions are expected to be overall accelerated in the direction opposite to the solar wind deflection. On the other hand, in the inner coma where the electric field is governed by the largely dominating cold new-born ions, the ion dynamics are expected to be tied to the radially expanding neutral molecules. In the observations, cometary ions of lowest energies were indeed found to expand with a main radial component away from the nucleus, whereas ions with higher energies are found to be precisely ordered by the upstream electric field direction (Nicolaou et al. 2017; Berčič et al. 2018; Nilsson et al. 2020), as reported and sketched in Fig. 11, two lower rows. The two cometary ion populations are referred to as respectively the expanding and the pick-up populations. Closer to the Sun, more complex flow patterns were observed around the diamagnetic cavity (Masunaga et al. 2019), for which the simple kinetic effects discussed above do not apply anymore, as the global regime transitioned from kinetic-like to fluid-like (cf. Fig. 10).

The 3-dimensional generalised gyromotion displayed in the lower row of Fig. 10 also presents “sideways”, asymmetrical dynamics, introduced when considering a magnetic field not perfectly perpendicular to the upstream flow direction, such as given by the classical Parker spiral. In the comet frame, lower-right panel of Fig. 10, the cometary ions are seen gyrating with only negative $v_{y}$ velocity components, while the solar wind protons have a positive $v_{y}$ component, no matter the sense of the magnetic field. This configuration is illustrated in a Sun-centred reference frame Fig. 3 of Behar et al. (2018b), and ultimately results in an observable dawn-dusk asymmetry, reported in the same publication.

Until now, the above-mentioned ion kinetic effects involved ion populations described as simple cold beams. A more comprehensive view of the dynamics can be given by the particle velocity distribution functions (VDFs). Observations of solar wind proton VDFs are reported in Fig. 2 of Behar et al. (2017). They find initially an anti-Sunward beam, which then gets increasingly deflected (as previously discussed). Around a deflection angle of 90 degrees, the beam transforms into a partial ring distribution, confirming that the solar wind indeed gyrates within the coma. At 2.1 AU, the proton VDF displays a partial ring which is not centred on zero, in the comet-centred frame. Accordingly, with the generalised gyromotion described previously, the central velocity is that of the electron fluid, which, based on this observation, reaches a few tens of kilometres per second, with negative $v_{x}$ and $v_{z}$, and no $v_{y}$.

The departure of cometary ion VDFs from a cold beam have been studied by Nicolaou et al. (2017), reporting occasional observations of energy-angle dispersion. While a first type of such a dispersion is consistent with ions gyrating in the local magnetic field (partial ring distribution), other VDFs show a second type of dispersion, which indicates either a local electric field significantly smaller than the upstream electric field, or the effect of a spatially heterogeneous plasma environment.

#### Electron Sources and Kinetic Electron Effects

The electrons observed in the inner coma of 67P essentially derive from two possible sources: 1) “cometary” electrons, produced in the ionisation of neutral molecules of cometary origin, and 2) solar wind electrons.

1. The cometary electrons are predominantly coma photoelectrons and secondary electrons, released when neutral molecules in the coma are ionised by solar EUV radiation or by other electrons with energy above the ionisation energy of the molecular species (cf. Sect. 2.2.1). Since the electron is so much lighter than the parent molecule and its daughter ion, it gains virtually all of the excess energy of this reaction. Substantial photoelectron production occurs up to energies of about 50 eV, with a quite sharp drop thereafter (Vigren and Galand 2013). Thus, coma photoelectrons initially have energies of up to several tens of eV. The energetic free electrons that are responsible for secondary electron generation may themselves originate either in the solar wind or from ionisation in the coma (either by solar EUV or previous generations of energetic free electrons). The basic energetics of the reaction is the same irrespective of the nature of the ionising particle (photon or electron) and the term “cometary electrons” is typically taken to include electrons resulting from either of these processes in the coma.
2. The electrons in the undisturbed solar wind are typically made up of a thermal core (electron energies $\lesssim50~\text{eV}$) and a suprathermal tail ($\sim70\text{--}1000~\text{eV}$), the latter in turn consisting of two distinct sub-populations: the nearly isotropic solar wind *halo electrons* and a magnetic-field-aligned beam of electrons known as the *strahl*. Typical temperatures of the core and halo electrons are about 5–10 eV (core) and 60–80 eV (halo) respectively in the range 1.4 AU to 3 AU (Pierrard et al. 2016).

There are a number of possible processes in the cometary plasma environment that can affect the source electron populations and contribute to sculpting the electron environment around the comet.

**The ambipolar electric field** is described in Sect. 2.3.3. Invoked by e.g. Madanian et al. (2016) and Myllys et al. (2019) as a possible acceleration mechanism for high energy electrons, as also seen in simulations by e.g. Deca et al. (2019), Divin et al. (2020).

**The electron convective electric field** can be interpreted as the sum of the ion convective electric field e.g. that of the solar wind and the Hall electric field. It is the primary mechanism that accelerates electrons away from the nucleus when they are no longer under the influence of the ambipolar electric field (Deca et al. 2019). This is usually in the solar wind dominated region of the coma.

**Adiabatic compression** Conservation of the first adiabatic invariant (magnetic moment) results in (perpendicular) electron heating of electrons moving along magnetic field lines into regions of enhanced magnetic field nearer to the nucleus (mentioned by e.g. Madanian et al. 2016; Nemeth et al. 2016; Broiles et al. 2016b). The density is also enhanced by this flow retardation, also leading to increased flux at all energies.

**Adiabatic expansion** (Perpendicular) cooling of electrons (and decrease of pitch angles) created in the region of enhanced magnetic field as they move outward along magnetic field lines into regions of weaker magnetic field. It is mainly proposed to occur in the decreasing magnetic field just before diamagnetic cavity observations by Madanian et al. (2020). The hypothesis of a double adiabatic evolution of either cometary or solar wind electrons in the cometary induced magnetosphere was tested in full kinetic modelling of solar wind-comet interactions (Deca et al. 2019).

**Collisional cooling** of electrons on neutral molecules in the cometary coma have predominantly been discussed for electrons of energies below about 20 eV (Madanian et al. 2016; Engelhardt et al. 2018a), where excitation of rotational and vibrational energy levels in the neutral molecules (predominantly H_2_O) dominate. This is further discussed in Sect. 2.3.5 below. At higher energies, other collisional processes become more important, such as electronic excitation and electron impact ionisation. Electron impact ionisation has been extensively discussed as a source of plasma (cf. Sects. 2.2.1 and 3.2.1) and FUV emissions (cf. Sects. 2.5 and 3.2.2). On the other hand, the possible impact of such processes on the energetics of the hot electron population so far lacks substantive discussion in the literature.

**Wave-particle interactions** e.g. acceleration of electrons by lower-hybrid waves has been mentioned by many authors, particularly by Goldstein et al. (2019) to explain very high energy (keV) electrons (discussed further below). The efficiency of such a heating mechanism is still being investigated (Lavorenti et al. 2021).

**Electrostatic shock potentials** Cross-shock potentials have been observed to accelerate electrons to energies of 100s of eV, e.g. near the Earth’s bow shock. This mechanism was discussed by Clark et al. (2015), but in the end it was discarded due to absence of a bow shock at the time of the observation. However, later when the infant bow shock was detected, it was often found to be accompanied by an electron population of enhanced energies (see Sect. 4.2).

From an observational point of view, further discussed below, three distinct electron populations were observed by Rosetta: a cold population (0.01–1 eV), a so-called warm electron population (4–50 eV), and a hot population (above 50 eV). The cold (resp. hot) population is believed to result from cooling (resp. heating) processes taking place in the induced magnetosphere of comet 67P. A similar result was found by Zwickl et al. (1986) from the brief ICE flyby of comet 21P/Giacobini-Zinner.

#### Electron Pressure and the Ambipolar Electric Field

Ionisation of the cometary gas by solar EUV radiation and electron impact liberates electrons with energies typically in the tens of eV range (see Fig. 12 in Vigren and Galand 2013), which corresponds to thermal speeds on the order of $1{,}000~\text{km/s}$. By contrast, the much heavier ions essentially retain the velocity they had as neutral molecules (typically $\lesssim 1~\text{km/s}$). As a result, the electron pressure is relatively high and has a strong inward gradient. The electrons set up an ambipolar electric field (Madanian et al. 2016; Deca et al. 2019) (see also Sect. 4.1). For the Giotto flyby of comet 1P/Halley, Gan and Cravens (1990) concluded that the ambipolar field could be neglected, because of the efficient electron cooling taking place at the very active comet. The activity of 67P varied between one and four orders of magnitude below that of 1P/Halley at the Giotto encounter (Sect. 2.1.1). In consequence, the ambipolar electric field and its effects on the local environment has been much more strongly emphasised in the Rosetta investigations.

**Fig. 12:**
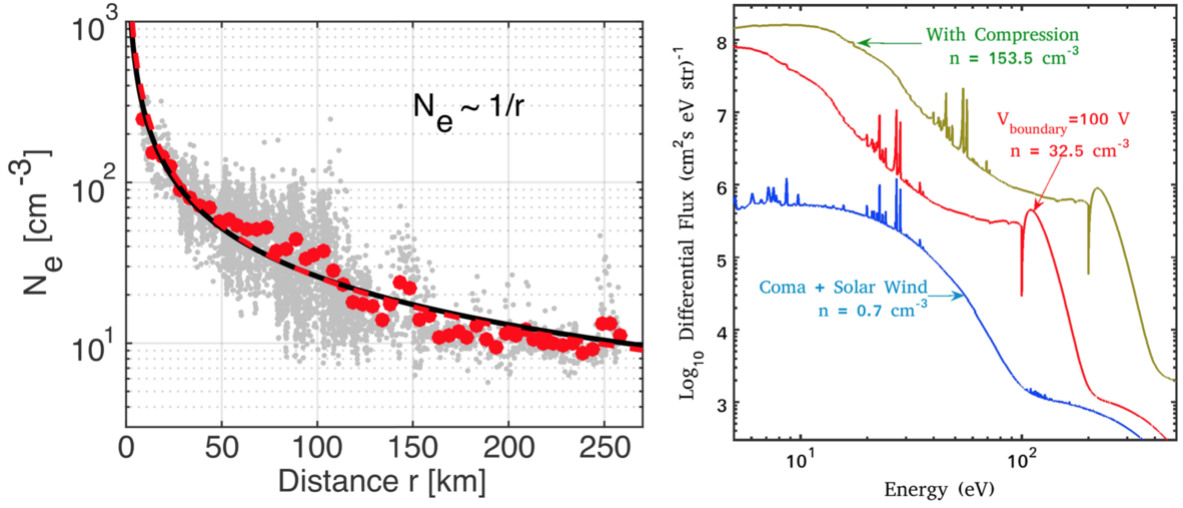
Left: Observed plasma density close to the nucleus during two flybys in February 2015 (Edberg et al. 2015). Right: The effects of an ambipolar electric field and of plasma compression on electron spectra in a kinetic model (Madanian et al. 2016)

Vigren et al. (2015a) assumed a model with $1/r$ profiles both for the ion density and the electric field. Their results imply ion acceleration up to the order of the ion acoustic speed. In other words, the typical ion flow kinetic energy becomes equal to the electron thermal energy. This is consistent with RPC observations (e.g. Berčič et al. 2018; Odelstad et al. 2018; Nilsson et al. 2020; Bergman et al. 2021). This can be seen in the third row of Fig. 11, showing that the radially expanding ion population (light blue arrows) has speeds of $\sim 10~\text{km/s}$, corresponding to energies of $\sim 10~\text{eV}$. Note as well that the plasma density given by this model underestimates the density expected if the ions were constrained by collisions to flow with the neutral gas, as assumed in some diamagnetic cavity models (cf. Sect. 4.6). The observed plasma density in the near-nucleus region indeed shows an approximate $1/r$ behaviour, as observed by Edberg et al. (2015) and Myllys et al. (2019) during close flybys (Fig. 12, left panel).

Madanian et al. (2016, 2017) used a two-stream electron model (Gan and Cravens 1990) that includes cometary sources and solar wind electrons to investigate the effects of the ambipolar field surrounding the cometary nucleus. They concluded that only when assuming a potential drop of $100~\text{V}$ at the boundary of the model, mimicking the effect of the ambipolar field, the model was able to reproduce the observed electron densities (right plot in Fig. 12). The ambipolar electric field temporarily traps cometary electrons and accelerates solar wind electrons, naturally leading to the observed “warm” (tens of eV) and “hot” (hundreds of eV and above) electron populations (Sect. 2.3.4). The ability of the ambipolar field to retain electrons for a longer time in the inner coma may also lead to increased cooling of electrons in non-collisionless plasma regimes (Sect. 2.3.5).

Particle-in-cell simulations confirmed that the formation of an ambipolar electric field near the nucleus results in solar wind electrons with elevated energies (Deca et al. 2017). A follow-up study by Deca et al. (2019) investigated the various terms of a generalised Ohm’s law to identify the driving physics in the various regions of the plasma environment of a weakly outgassing comet. The self-consistent kinetic simulations showed the importance of the electron pressure gradient and justified the $1/r$-scaling assumed in the simplified model. The polytropic index of the cometary electrons (contrary to solar wind electrons) within $50~\text{km}$ from the nucleus was found to be close to 1, supporting also the assumption of isothermal electrons within the dense, inner coma region in the analytical ambipolar electric field models (see discussion around Eq. (2.3.3)).

**Fig. 13:**
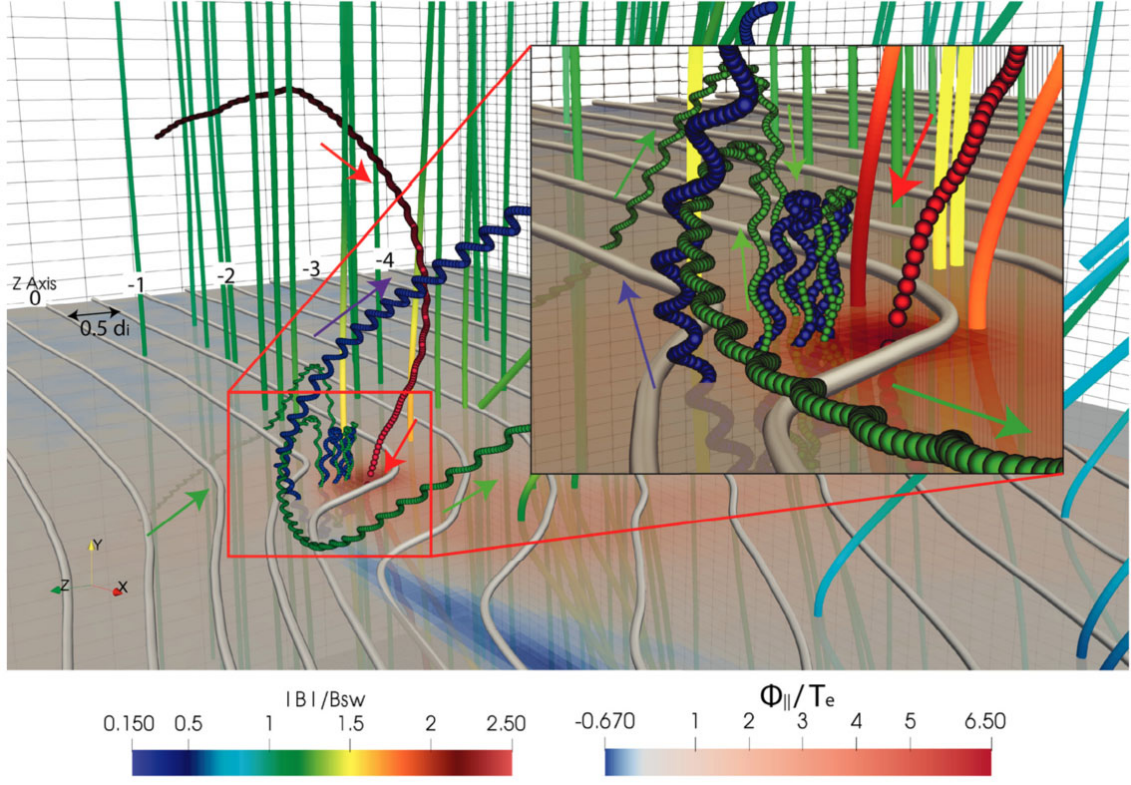
The electron environment close to the nucleus in a particle in cell simulation. For description see text. From Sishtla et al. (2019)

Sishtla et al. (2019) characterised the trajectories of trapped electrons in the ambipolar potential well surrounding the nucleus and defined a clear boundary in velocity space separating temporarily trapped cometary electrons from passing solar wind electrons. Figure 13 illustrates the different classes of electron trajectories identified in the simulation. The nucleus itself is not shown, but resides in the centre of the region of increased ambipolar potential indicated by red shading in the middle of the zoomed insert. The vertical lines are magnetic field lines, colour-coded by the magnetic field strength. Mass loading and partial flow stagnation leads to higher magnetic field magnitudes near the nucleus. The spiralling lines show the motion of three selected electron trajectories, with direction of motion indicated by similarly coloured arrows. A solar wind electron (red spiral curve) is seen to come in from the back of the figure, be deflected and accelerated along the magnetic field by the ambipolar field toward the region near the nucleus, where also its gyroradius decreases and gyrofrequency increases as the magnetic field grows stronger. It crosses the (magnetic) equatorial plane and leaves to the solar wind again on its lower side. The green and blue spiral curves show cometary electrons spending a long time semi-trapped by the combined effects of the magnetic and the ambipolar electric fields near the nucleus, with a bounce motion along $\vec{B}$ due to the outward directed electric field combined with spiral and drift motion. There are no collisions in these simulations, but they nevertheless show that temporary trapping can occur in realistic geometries and can make the electrons stay for much longer in the region of dense neutral gas around the nucleus, thus increasing the cooling rate as discussed in Sect. 2.3.5. In terms of Ohm’s law, it is caused by the asymmetry of the Hall term (Deca et al. 2019).

Divin et al. (2020) concentrated on electron acceleration and pitch angle distributions from cometary plasma kinetic modelling. They found that for the $Q = 10^{25}~\text{s}^{-1}$ case studied, solar wind electrons were accelerated along the magnetic field to energies of 50–70 eV, and found electron spectra qualitatively similar to observations (see Sect. 2.3.4). As shown by Galand et al. (2020) and discussed in Sect. 2.5, these accelerated electrons are of special interest as they can give rise to auroral emissions. As discussed in Sect. 4.1, the electron pressure is also related to the polarisation field investigated by Nilsson et al. (2018) and simulated by Gunell et al. (2019). Simulation codes and numerical models are further discussed in Sect. 3.3.

#### Warm and Hot Electrons

In general, we define three different electron populations at the comet: cold, warm and hot. Generally, the warm population constitutes the bulk of the density, with occasionally cold electrons dominating. Definitions of the energy ranges are not always the same, therefore we present a summary of the electron populations in Table 2.

**Table 2:** Summary of the three different electron populations by temperature. The last column gives the most common generation mechanisms

	Temperature (eV)	Generation
cold	$\lesssim 1~\text{eV}$	collisions
warm	$4\text{--}50~\text{eV}$	photoelectrons, solar wind core
hot	$\gtrsim 50~\text{eV}$	acceleration, solar wind strahl

As Rosetta came within a few tens of kilometres of the weakly active comet nucleus in August 2014, the electrons observed by RPC-IES transitioned from exhibiting typical solar wind-like bi-Maxwellian behaviour to a distribution with energies of several tens up to hundreds of eV (Clark et al. 2015). The observed electron signatures were highly dynamical, with large variations in differential flux in both time and energy. Such electrons remained a staple of the observations throughout the entire comet escort phase of the Rosetta mission (Myllys et al. 2019). The count rates below 100 eV steadily increased until December 2014 and peaked between January and February of 2015 before gradually decreasing again (Broiles et al. 2016a) as Rosetta’s distance to the nucleus increased again and the comet became more active.

**Fig. 14:**
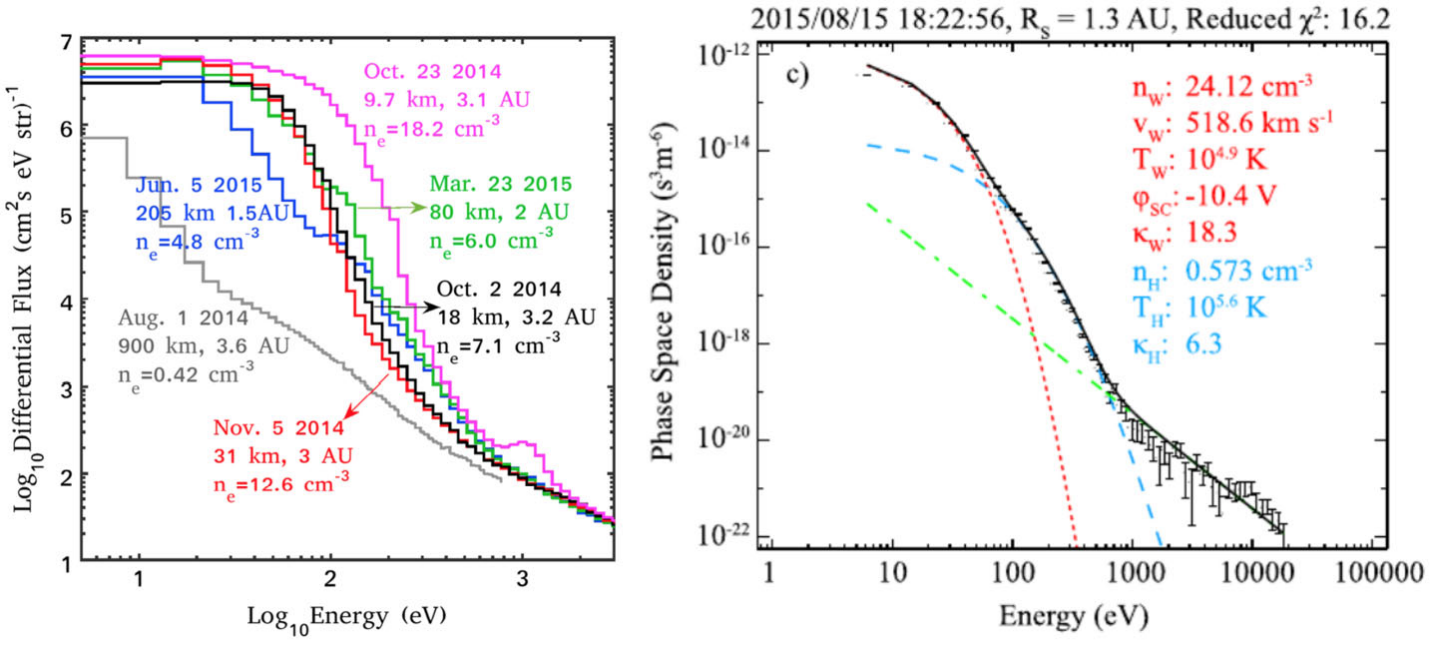
Left: Observed IES electron spectra (daily averages) for selected intervals. The grey spectrum (Aug 1, 2014) is typical for the solar wind, the other represent various activity stages and cometocentric distances (Madanian et al. 2016). Right: An example IES electron spectrum with fit to two kappa distributions. The high kappa value $\kappa _{w} \approx 18$ of the warm population ($T_{w} \approx 7~\text{eV}$) means it is close to Maxwellian, while the lower $\kappa _{h} \approx 6$ implies a significantly stronger high energy tail in the hot ($T_{w} \approx 34~\text{eV}$) population (Broiles et al. 2016b)

Broiles et al. (2016a) fitted two kappa distributions to IES observations for two separate days far from and close to perihelion (30 October 2014 and 15 August 2015, see right panel of Fig. 14). The $\kappa $ values of the warm population were found rather high and ranging from 10–1000 (with variations in this range on time-scales shorter than the 128 s measurement cycle of IES), suggesting the population was near thermal equilibrium.^4^ The $\kappa $ values of the hot population were lower, typically in the range 1–10, indicative of a population much further from thermal equilibrium. Myllys et al. (2019) studied these warm and hot electron populations over the full comet escort phase and characterised the electron properties as a function of cometocentric distance. Based on data from the two close flybys by Rosetta on Feb 4 and 28, 2015, a $r^{-1.2}$ dependence with density was found for the warm population, i.e., close to the $\sim r^{-1}$ dependence for the bulk density found by Edberg et al. (2015) for these same flybys (12). It suggests that the warm population consists mostly of cometary electrons (i.e., electrons created during the ionisation processes). The latter is in agreement with self-consistent numerical modelling results (Deca et al. 2017).

For the hot population, Myllys et al. (2019) found a $r^{-2.1}$ dependence with density. Data from the dayside excursion (out to $\sim1500~\text{km}$ between 2015-08-01 to 2015-10-20) corroborated the $\sim r^{-1}$ dependence of the warm population out to about 900 km, after which a weaker dependence ($\sim r^{-0.7}$) was observed. For the hot population on the other hand, the density was quite constant or even increased somewhat as the spacecraft altitude became higher; thus a different behaviour from the $\sim r^{-2}$ dependence observed months earlier during the close flybys. Neither population’s temperature exhibited any dependence on cometocentric distance.

Overall, when accounting for the radial cometocentric dependence of the warm population, the densities of both populations increased with decreasing heliocentric distance, as might be expected when both the photo-ionisation frequency and neutral gas density increased. The density of the warm population increased from about 30 to $100~\text{cm}^{-3}$ between 3.5 and 1.3 AU, whereas the density of the hot electrons increased from about 0.1 to $3~\text{cm}^{-3}$. The temperature of the warm population on average did not change with heliocentric distance, while the hot population showed a modest increase by about 33%.

Various studies have addressed a possible link between the hot electron population observed at Rosetta and the solar wind electrons. The hot population density observed by RPC-IES was almost one order of magnitude higher than the suprathermal halo density in the solar wind (Myllys et al. 2019), although this was suggested by Broiles et al. (2016a) to be the source of the hot population. However, the solar wind *core* electron density was the same order of magnitude as that of the hot population, and both decreased with increasing heliocentric distance, so it was suggested that the hot population actually consisted of solar wind *core* electrons that had been accelerated by the ambipolar electric field. PIC simulations by Deca et al. (2017) appear to largely support this notion, at least for the very low activity rates in their simulations. However, Myllys et al. (2019) concluded acceleration in an ambipolar electric field is insufficient for providing the hot population energies that extend to $\sim100~\text{eV}$ and beyond. Thus, this high-energy tail of the distribution, which only has a minor contribution to the total density of the population, can possibly be explained as deriving from the suprathermal component of the solar wind electron distribution. Madanian et al. (2020) suggest that the solar wind electrons entering the cometary magnetosphere are mainly strahl electrons. The differential fluxes they observed (for the single event that they study) are higher than the typical flux of solar wind halo electrons at 1 AU, but lower than the typical strahl component.

Goldstein et al. (2019) reported on intermittent measurements by IES of up to $\sim1~\text{keV}$ electrons on 19 January 2016, at about 2 AU from the Sun and 80–100 km from the comet nucleus. Roughly moving anti-sunward, they exhibited beam-like behaviour and were suggested to have been excited by wave activity, specifically a two-stream instability. No further statistical survey of the occurrence of such $\sim1~\text{keV}$ electrons throughout the mission was done.

#### Cold Electrons

Although the Giotto spacecraft did not carry any instrumentation to directly measure the temperature of any low energy electron population that might be present, it was theorised that the inner coma of comet 1P/Halley was sufficiently dense to efficiently cool electrons by collisions with the molecules of the neutral gas (e.g. Feldman et al. 1975; Ip 1985; Eberhardt and Krankowsky 1995). The initial data from the much less active comet 67P showed no evidence of a cold electron population when arriving at the comet.

Mandt et al. (2016) used neutral density data obtained by the ROSINA-COPS instrument to calculate the distance of the electron exobase (or collisionopause, see also Sect. 4.5), outside which electron cooling was expected to be inefficient. The period under study was April–September 2015, corresponding to heliocentric distances of 1.7 to 1.25 AU. The study concluded that Rosetta must have stayed well outside the electron exobase during this time, consistent with the observations of warm and hot electrons discussed in Sect. 2.3.4.

**Fig. 15:**
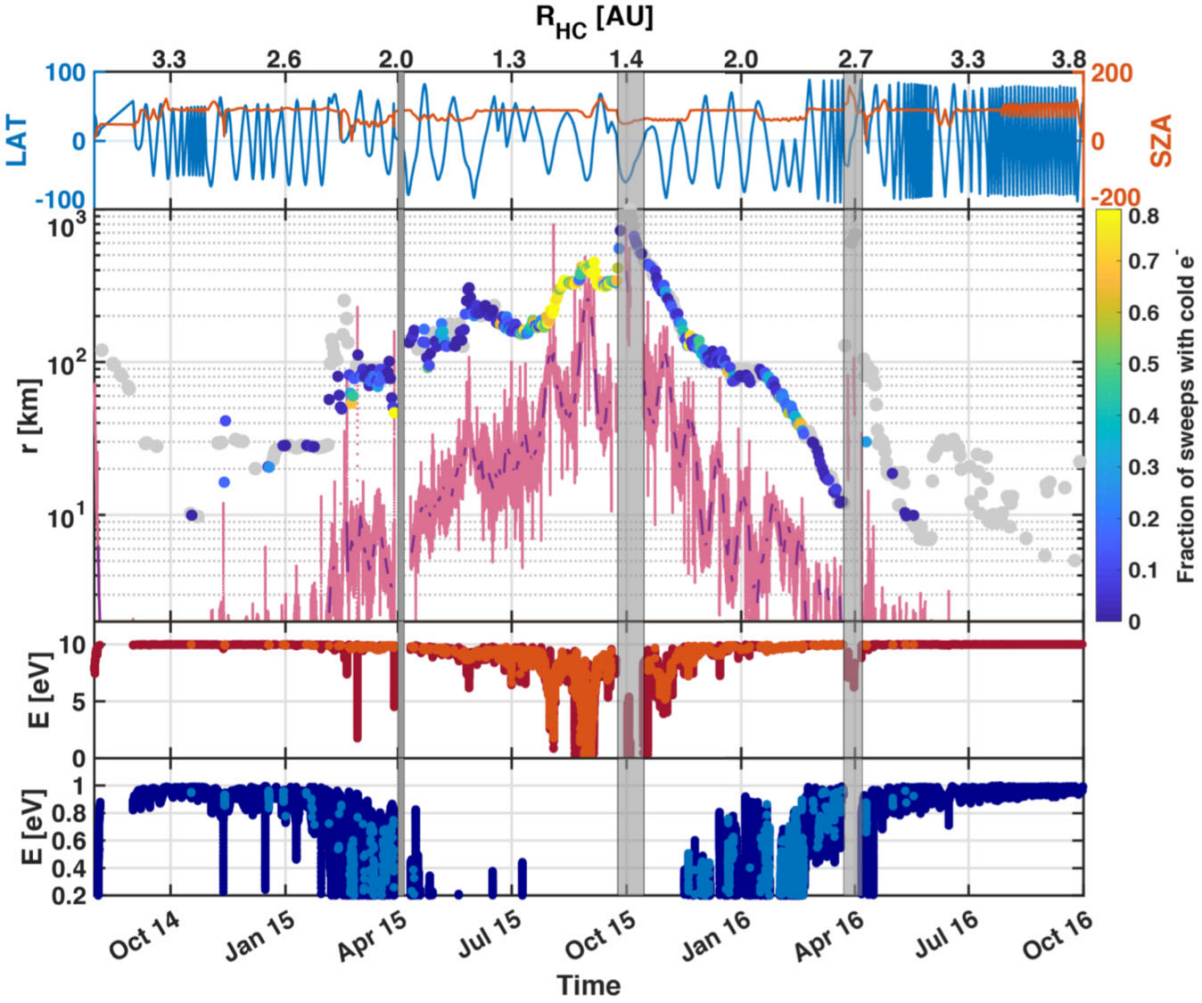
Presence of cold electrons during the Rosetta mission according to RPC-LAP is colour coded in the large panel, the red line indicates the electron exobase distance. The first panel shows the latitude (blue) and solar zenith angle (orange) of the spacecraft. The two bottom panels show the final energies when reaching Rosetta position of two electrons starting near the nucleus at initial energy 10 eV and 1 eV, respectively, using a simple cooling model and the neutral gas density observed by ROSINA-COPS. Grey overlays indicate times of excursions to larger distance from the nucleus. From Engelhardt et al. (2018a), reproduced with permission ©ESO

However, consideration of a slightly more elaborate cooling model, integrating a loss function including rotational and vibrational cooling, showed that cooling could be significant at least for electrons originating close to the nucleus and hence passing through the densest gas as seen in the lower two panels of Fig. 15 for electrons starting at the nucleus surface at 1 and 10 eV, respectively (Engelhardt et al. 2018a). It was suggested that further cooling could be brought about by the mechanism noted by Madanian et al. (2016), i.e. that the ambipolar electric field keeps them inside the dense region, thereby increasing the time available for cooling (see also discussion of Fig. 13 in Sect. 2.3.3). Engelhardt et al. (2018a) also noted that another effect of the ambipolar field simply is to slow down the electron as it moves outward. This not only is a direct decrease of its kinetic energy but also increases its collision cross section, further increasing cooling.

Nevertheless, evidence of cold electrons in both the Langmuir probe (LAP) and the mutual impedance probe (MIP) data was found throughout this period and it was conjectured that the electron gas was a mix of warm electrons created outside the exobase and cold electrons cooled in the dense neutral gas further inside the coma (Eriksson et al. 2017). Such cold electrons might be associated to the diamagnetic cavity (Sect. 4.6). The interpretation of the LAP probe current pulses as structures of cold plasma akin to the regions of higher density in the hybrid simulations by Koenders et al. (2015) was iterated in an extended study of them through all the Rosetta mission (Engelhardt et al. 2018b). This interpretation is consistent with the results by Edberg et al. (2019a), who found that the cold electrons (as well as the diamagnetic cavity) mainly are found on the side of the nucleus where the solar wind convective electric field points toward the nucleus.

Engelhardt et al. (2018a) combined MIP and LAP measurements to establish an unambiguous signature of cold electrons in the LAP data. Their mission summary plot is shown in Fig. 15, showing the first observations already during the Rosetta 10 km orbits in October 2014, at around 3 AU when the gas production rate was as low as $10^{26}~\text{s}^{-1}$ (Hansen et al. 2016). Days without observations of cold electrons (grey circles) were rare inside 2.3 AU, and around perihelion cold electrons were detected almost continuously.

The LAP and LAP-MIP observations of a mix of cold and warm electrons were confirmed and extended through a fully independent method based on MIP measurements alone (Gilet et al. 2017). For all intervals where LAP had detected cold electrons and MIP were operated in a suitable mode, MIP confirmed their presence (Gilet et al. 2020). Interestingly, the opposite was shown not to be true: MIP also observed cold electron detections without corresponding LAP detections. It is believed that such cold electrons could not reach the LAP probe because of the strongly negative spacecraft charging, which would prevent cold electrons from reaching the LAP probes.

**Fig. 16:**
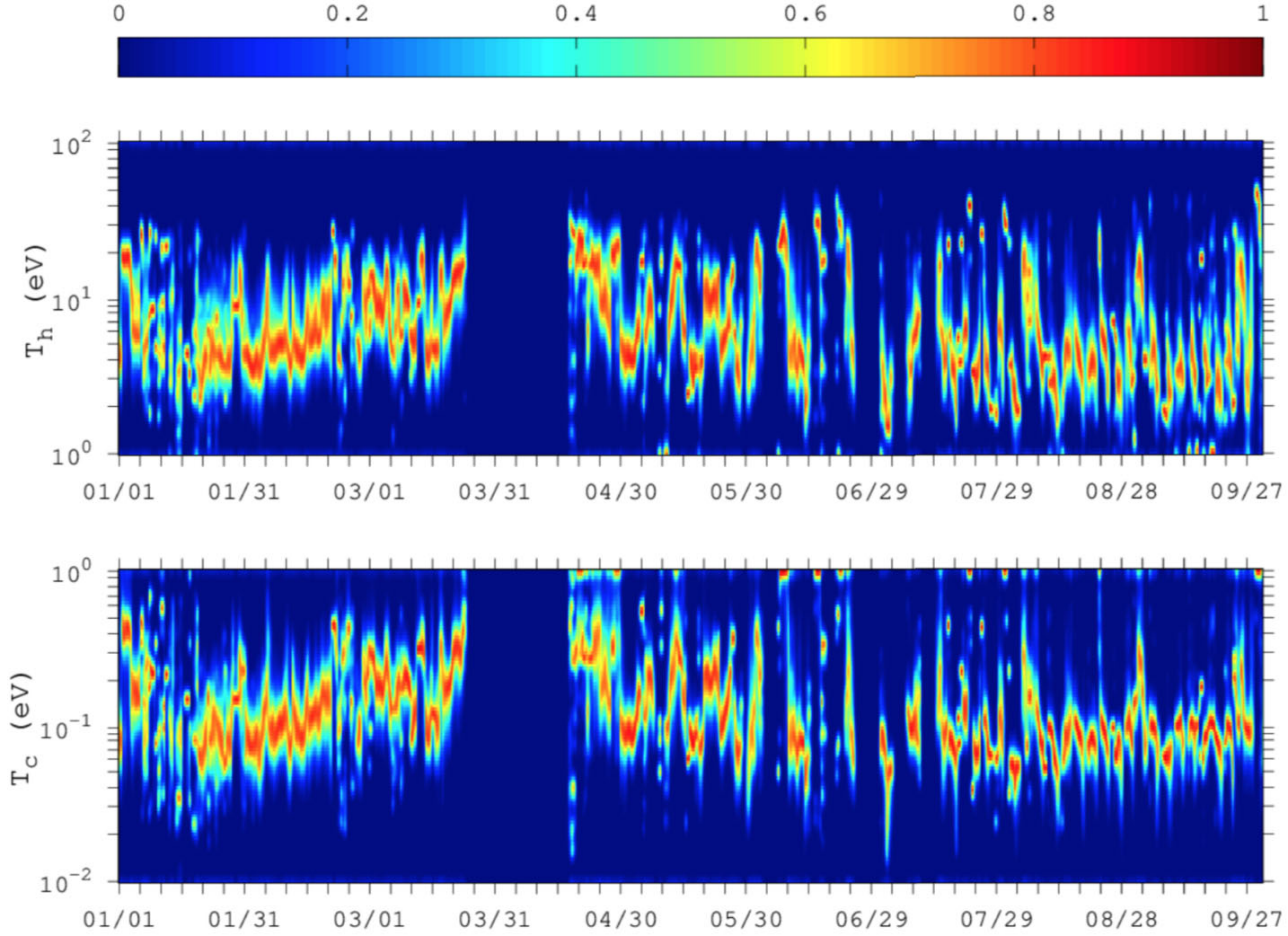
Warm (top) and cold (bottom) electron temperatures derived from RPC-MIP spectra. The colour bar shows the normalised occurrence, calculated for 6 h intervals, so each column can be seen as a colour coded histogram. From Wattieaux et al. (2020), reproduced with permission ©ESO

In a later study, Wattieaux et al. (2020) were able to provide densities as well as temperatures of both populations for the time period January–September 2016 using MIP mutual impedance spectra. They reported that the temperature of the warm (referred to as “hot” in that study) population varied between about 3 and 20 eV, as expected for photoelectrons, while the cold population mostly stayed in the range 0.05 to 0.3 eV (Fig. 16). Interestingly, a significant correlation between the temperatures of these two population was noted. Warm electrons most of the time made up between 10% and 30% of the whole population, and the cold electron population was observed to typically dominate the cometary plasma.

An intriguing result regarding the connection of electron cooling to large scale structures was obtained by Henri et al. (2017), who could show that the Rosetta observations of a diamagnetic cavity was very well organised by the distance to the electron exobase (Sect. 4.6). In addition, Odelstad et al. (2018) found that this region stands out also regarding presence of cold electrons, as almost all LAP bias voltage sweeps acquired during Rosetta diamagnetic cavity observations showed signatures of cold electrons. For the small minority not doing so, it was suggested the very negative spacecraft potential $V_{s}$ blocked the electrons from accessing the probes.

The evolution of the Rosetta spacecraft potential $V_{s}$ has been reported by Odelstad et al. (2015) and Odelstad et al. (2017), the latter including data from the full mission and using data from RPC-ICA as well as RPC-LAP for cross-validation of the results. The spacecraft potential was mainly found to be negative, typically in the range $-5~\text{V}$ to $-10~\text{V}$ but during long periods well below $-10~\text{V}$ and sometimes going below the LAP range of $-30~\text{V}$, as also seen in e.g. the ICA ion spectra published by Stenberg Wieser et al. (2017). The negative $V_{s}$ was initially interpreted as due to high fluxes of electrons in the tens of eV range and hence seen as evidence of the continuous presence of the warm electron population. However, Johansson et al. (2020) noted that the observed $V_{s}$ correlates better with the density of the cold electron population than with the warm, which was unexpected as the cold electrons should not be able to reach the strongly negative spacecraft and thus not be responsible for its charging. The explanation was found in positively (up to +70 V) biased conductive elements forming part of the solar cell strings on the solar panels. Johansson et al. (2020) modelled how these efficiently collect cold electrons and thereby push $V_{s}$ down to large negative values. So while the $V_{s}$ statistics and measurements by Odelstad et al. (2015) and Odelstad et al. (2017) are correct, the strong negative charging in fact is more due to cold electrons than to the warm population originally invoked. However, there is good evidence for the continuous presence of the warm population in any case, as seen in data from LAP, MIP and IES (e.g.  Madanian et al. 2016; Broiles et al. 2016b; Eriksson et al. 2017; Myllys et al. 2019; Gilet et al. 2020; Wattieaux et al. 2020). As yet, no observation of only cold electrons has been reported at 67P.

#### Dust-Plasma Interaction

Comets have been suggested as suitable dust-plasma laboratories (e.g. Mendis and Horányi 2013) and providers of nanodust to the solar system (e.g. Mann 2017). Investigations of the cometary dust have been one of the main themes in Rosetta data analysis, but this is a big subject in its own and far outside the scope of this review. Here we concentrate on the evidence of dust-plasma interactions yet identified, with a particular eye to the influence of dust on the ionisation balance in the coma of 67P. The take-home message is that despite some direct evidence of charged nanograins near the nucleus and indications of an extended upstream nanograin population it still remains unknown whether or not Rosetta ever encountered an electron depleted plasma in the coma, meaning a region where the positive ion number density markedly exceeds the number density of free electrons, though published results have not shown any evidence for this. Acceleration of charged dust particles by the electric fields arising from the interaction of the solar wind and comet plasmas have been invoked to explain some Rosetta dust observations, but no feedback from the dust on the plasma has yet been identified.

The simplest analytical cometary ionospheric models (described in Sect. 3.2.1) assume plasma to move radially outward with the neutral expansion velocity and to not undergo any loss process. Such a model makes it possible to predict the electron number density via the measured ambient neutral number density, an assumption of the expansion velocity and an estimation of the ionisation frequency (e.g. Galand et al. 2016; Vigren et al. 2016). On the one hand, studies have shown that such a model, at least in an average sense, works well at low to moderate activity and close to the nucleus (e.g. Galand et al. 2016; Vigren et al. 2016; Heritier et al. 2017b). On the other hand, studies have also shown that near perihelion the model predicted electron densities often greatly exceed the observed values, sometimes even by an order of magnitude (e.g. Vigren et al. 2017; Henri et al. 2017; Heritier et al. 2018; Vigren and Eriksson 2019; Beth et al. 2019; Hajra et al. 2020). Multiple reasons for this discrepancy have been suggested and it is likely that it comes down to some combination of attenuation of the ionising UV radiation (Johansson et al. 2017), loss via dissociative recombination (Vigren et al. 2015a; Heritier et al. 2018; Beth et al. 2019), outward radial ion acceleration (Vigren and Eriksson 2017; Vigren et al. 2017; Odelstad et al. 2018), non-radial plasma motion and grain charging. Not much attention has been given to the possibility of a pronounced level of electron depletion due to grain charging around active comets despite the fact that the process plays a very central role in the ionisation balance of the water dominated geysers of Enceladus as found in Cassini measurements (Morooka et al. 2011, see also below).

Vigren et al. (2015b), using the grain-charging formalism of Draine and Sutin (1987), showed that if the escaping gas carries aloft nanograins, contributing to 1% of the nucleus mass loss, a significant level of electron depletion would be possible up to cometocentric distances of several 100 km during active phases. The study showed also that while larger grains (tens to hundreds of nm) can acquire multiple negative charges. These grains are unlikely to contribute significantly to the overall level of electron depletion in the plasma. The reason for this is the well-known fact that if a large grain fragments into smaller units, the total charge that can be absorbed by the dust increases, which can be understood as follows. The random thermal motion of electrons will be efficiently repelled and the electrons therefore stop charging a dust grain when it reaches a potential a few times $-k_{B} T_{\mathrm {e}}/e$. As capacitance scales with linear dimension, not with volume, fragmentation of a dust grain into $N$ equal parts increases the total charge carried by the dust by a factor $N^{2/3}$. As an example, consider a cubic dust grain splitting into 8 cubes of half the linear size; the total capacitance clearly increases by a factor of $8/2 = 4 = 8^{2/3}$, and so does the number of electrons attached to the grains and therefore lost from the plasma (at least until the plasma becomes strongly electron depleted).

The plasma effect due to dust is in general terms (e.g. Meyer-Vernet 2013) expected to be pronounced in “dusty plasma scenarios” where the intergrain distance is smaller than the Debye length while smaller in “dust in plasma scenarios” where the individual grains are isolated, as being separated by distances exceeding the Debye length. For reasons discussed below, it was not possible for Rosetta to infer the total charge density bound to dust grains by independent measurements of both the ion and electron densities in the way this could be done by Cassini in the Enceladus plume (Morooka et al. 2011).

Nevertheless, indirect observations indicate that nanodust grains are rare close to the nucleus but much more common far upstream, suggesting that any significant dust effect on the charge balance should be more pronounced at large cometocentric distance. Johansson et al. (2017) compared the photoemission (emission of photoelectrons) from RPC-LAP with extrapolated solar EUV (extreme ultraviolet) spectra from TIMED/SEE (orbiting Earth) and MAVEN (orbiting Mars) and showed that the proportionality factor decreased markedly with the cometary activity. For instance, near perihelion the photoemission was $\sim50$% of the value that would be predicted from extrapolating solar EUV spectra and using the inverse square dependence on heliocentric distance established at lower activity (both pre- and post perihelion). The authors noted that the reduced photoemission cannot be attributed to attenuation of the EUV intensity by absorption in the upstream gas and entertained instead the possibility of an attenuation caused by nanometre sized dust particles. The relative reduction in photoelectron emission showed no correlation with the cometocentric distance and persisted even during the dayside excursion (to about 1,000 km cometocentric distance). This suggested, to Johansson et al. (2017), that the bulk nanograin population causing the attenuation resided further upstream, likely originating from fragmentation and/or erosion of larger grains.

**Fig. 17:**
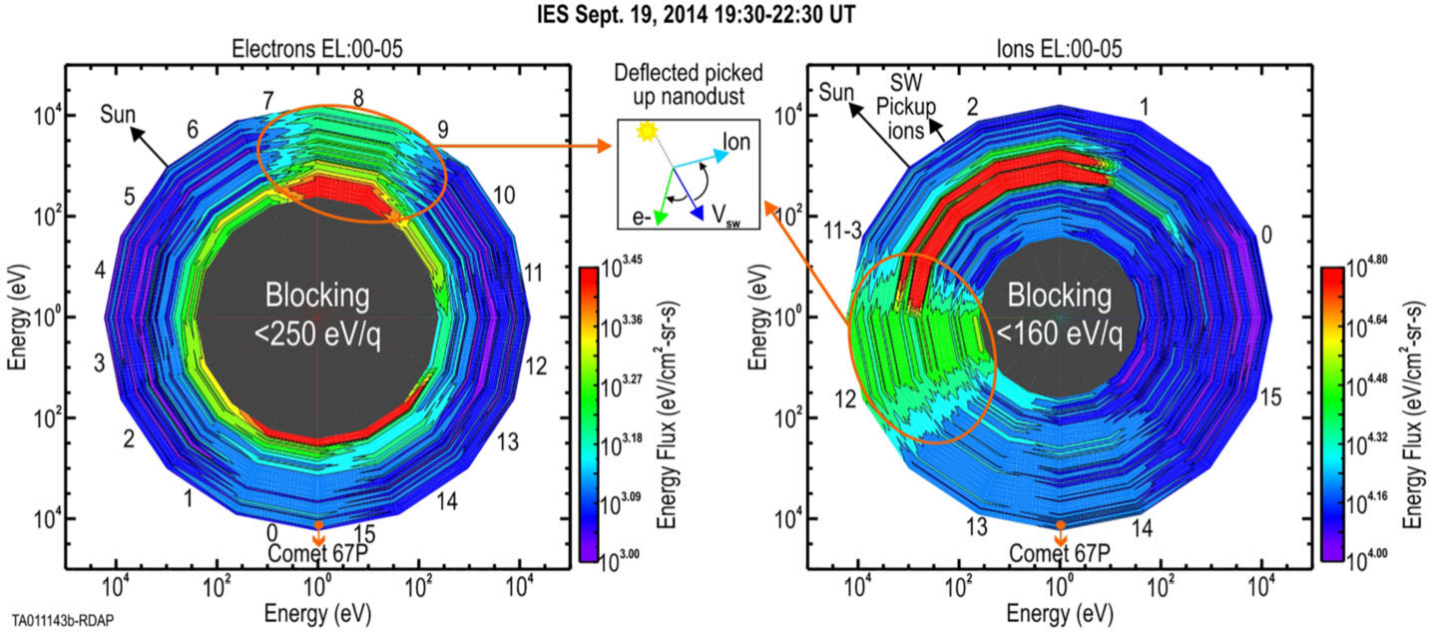
Observation of oppositely charged energetic nanograins by the Rosetta Electron and Ion Sensor, RPC-IES. The energy flux of negative (left, denoted “Electrons”) and positive (right, denoted “Ions”) dust grains is colour coded in this energy-azimuth polar plot from which low energy particles have been excluded. Detections of particles of both charge signs over a wide energy range are indicated by the orange ellipses, with their velocity directions compared to the solar direction shown in the insert. This is one of the few (as yet) published pieces of direct Rosetta evidence of dust-plasma interactions. Figure from LLera et al. (2020)

Occasional direct RPC-IES observations of charged nanograins moving antisunward (due to the solar radiation pressure) were reported by Burch et al. (2015b). Gombosi et al. (2015) found the observations consistent with grains of radius 30–80 nm accelerated antisunward mainly by the radiation pressure. It can be noted that Szegö et al. (2014) calculated that if the nanodust at 67P constituted the 6% of the nucleus total dust emission inferred by Utterback and Kissel (1990) for 1P/Halley, the flux of charged nanodust from the nucleus direction would be possible to measure by RPC-IES. No such observations have yet been reported. Instead, the nanodust events found by RPC-IES show antisunward directed fluxes, with some deviation that could be interpreted as the effect of electric fields. The latter is particularly clear in the simultaneous detection of positive as well as negative nanograins by LLera et al. (2020), where the deviation from antisunward motion is opposite for oppositely charged grains (Fig. 17). Mainly antisunward motion of submicrometer- to micrometer sized grains was found also by the GIADA dust analyser (Della Corte et al. 2019).

To better constrain how large fraction of the electrons were bound to dust grains at 67P, Vigren et al. (2021) considered equilibrium charging of a dust population synthesized from Rosetta observations by Marschall et al. (2020). The conclusion of this study was that because of the dominance of large grains, the typical value of this fraction likely was below 10% and possibly less than 1%. Photoelectron emission counteracts the negative charge and may even (depending on the electron temperature) charge grains predominantly positively, but a follow-up study showed that positive grains also cannot carry a significant amount of the total charge in the plasma Vigren et al. (2022).

These findings can be taken as a verification of the lack of emission directly by the nucleus of particles in the sub-micrometre range, though there may be complications due to the significant negative charging of Rosetta itself (Odelstad et al. 2017; Johansson et al. 2020) which could deflect negative grains away from the spacecraft (Fulle et al. 2015). The presence of positively charged elements on the solar panels (Johansson et al. 2020) can only locally alleviate this effect and does not explain the general lack of observations. In any case, the antisunward grain motion fits very well to the scenario with fragmentation and/or erosion at a distance, as the radiation pressure most efficiently accelerates grains at or below $\sim 100~\text{nm}$ size (Gombosi et al. 2015). The electric force, which deflects particles off the antisunward direction, is most efficient for even smaller grains, but the simulations by Gunell et al. (2015) showed that also grains in the 10 nm range may follow electric field lines through the coma. The intermittency of the RPC-IES nanodust could then be due to the variations of the large scale electric field (see Sects. 4.1 and 2.3.2) in the comet plasma environment (Gombosi et al. 2015).

Consistent interpretation of remote observations of dust at comets also requires the presence of smaller grains (Combi 1994) at cometocentric distances much larger than Rosetta’s. Such observations also show that even further upstream, the density of such grains is depleted as they are pushed back by the solar radiation pressure (e.g. Boehnhardt et al. 2016). Reconciling the in situ observations of the near-nucleus environment with the remote observations of the more distant coma implies some sort of fragmentation or erosion processes are active. What these may be is outside the scope of this paper, but we may note that the large aggregate dust grains found by Rosetta show low tensile strength (Hornung et al. 2016) and thus are sensitive to fragmentation and erosion mechanisms such as electrostatic repulsion (e.g. Hill and Mendis 1981) and the centrifugal force from increasing rotation speed of dust grains due to direct solar radiation (Herranen 2020) or sublimation driven effects (Steckloff and Jacobson 2016). Gas spectrometer measurements have shown that some minor species, HF, HCl and HBr, emanate from an extended distributed source. This indicated that these species are evaporated from icy grains in the coma, showing the presence of such dust grains. However, the contribution from these grains to the water atmosphere was found to be insignificant (De Keyser et al. 2017).

The Cassini observations of an ion and electron density mismatch from which the dust charge density was inferred was possible because the ion density could be measured by the Langmuir probe of the RPWI instrument (Gurnett et al. 2004). It should be repeated that while Cassini probed Saturn, Enceladus and Titan at relative speeds of $\sim30\text{--}35~\text{km/s}$, $\sim17~\text{km/s}$ and $\sim6~\text{km/s}$, respectively, Rosetta moved extremely slowly (typically below 1 m/s) around the nucleus of 67P, with the result that the equivalent instrument on Rosetta (Eriksson et al. 2007) could not provide an independent ion density to sufficient accuracy (on both spacecraft, the Langmuir probes as well as independent techniques based on plasma resonances could establish the electron density). If Rosetta had moved faster, enabling independent ion and electron density measurements, would there have been any hint of a mismatch due to dust charging? From the theoretical models applied to dust observations as described above (Vigren et al. 2021, 2022), the likely answer is no. This contrasts to the strong electron depletion inferred for the Enceladus plume (Morooka et al. 2011; Hill et al. 2012) and Titan’s ionosphere (Coates et al. 2007; Shebanits et al. 2013). The key to this difference seems to be the very different properties of the dust from the objects in question, relating to the their equally different dust production processes, despite the apparently comet-like attributes of Enceladus (Boice and Goldstein 2010). At Enceladus and Titan, the dust particles are thought to be continuously produced and processed, from liquid water expanding as jets through the nozzle of cracks in the ice cover (Porco et al. 2006; Hsu et al. 2015) and by photochemical processes in a complex atmosphere (Waite et al. 2007), respectively. In both cases dust formation is ongoing, starting at small size. At the comet the understanding is radically different: the nucleus was once formed by large dust grains virtually untouched by the weak gravity binding the body together, as indicated by the resemblance of the dust aggregates at 67P to interplanetary dust particles (Langevin et al. 2016; Bentley et al. 2016; Mannel et al. 2016). Now these large grains are released as the volatile part of the solid material on the surface sublimates due to solar heating. The grains apparently leave the surface mainly under the influence of a gentle gas pressure gradient and/or weak electrostatic forces, barely sufficient to lift the grains which therefore do not immediately fragment to the tens of nm sized parts they have been shown to be comprised of Bentley et al. (2016), Mannel et al. (2016).

So how can we best summarise the Rosetta results on dust-plasma interactions at comets, five years after end of mission? The main finding is that no such effects have been obvious in the plasma data. Rosetta investigations of dust and of plasma have provided a wealth of new knowledge and understanding and changed the agenda of the scientists investigating each field, but regarding their interaction almost nothing have as yet been found from the data. This negative finding may to some extent be due to the measurement conditions and also to the complexities of the data set, where understanding of the plasma and dust observations on their own have taken years and is far from finished. Nevertheless, it must be said that the picture of a near-nucleus dust environment dominated by large grains is entirely consistent with the absence of conspicuous dust-plasma interaction signatures. Large grains means a small fraction of the plasma electrons adsorbed on the dust particles, so that the dust effect on the plasma is small. Conversely, the big and heavy dust grains are relatively insensitive to the electric fields the plasma may set up.

However, much remains to explore in the Rosetta datasets. Even if the picture we paint here, with limited dust-plasma interaction in the innermost coma, would mainly hold true also in the coming years of continued Rosetta data analysis, it might well be that the situation could be different at particular events. For example, could there be strong nanodust releases at outbursts from the nucleus, or some high energy electron events causing so much charging of dust grains that electrostatic fragmentation near the nucleus becomes very strong, yielding lots of sub-micron grains and corresponding dust-plasma interactions effects? There may still be surprises awaiting us even in the existing datasets.

#### Waves

Waves are important in the cometary plasma, because they can transfer energy and heat the particle populations (Krall and Trivelpiece 1973; Tsurutani and Oya 1989; Treumann and Baumjohann 1997). Plasmas react to contain the effects of perturbations through Debye shielding, but when the wave modes of a plasma are excited, these waves can travel long distances. Typically, waves are created by instabilities, they travel through regions where they are weakly damped and deposit their energy in regions of strong damping. It is through steepening of waves that shocks are created, and waves provide dissipation in collisionless shocks (see Sect. 4.2). Waves can be used as a remote sensing tool as the detection of waves provides information of the remotely located place where they were generated.

**Fig. 18:**
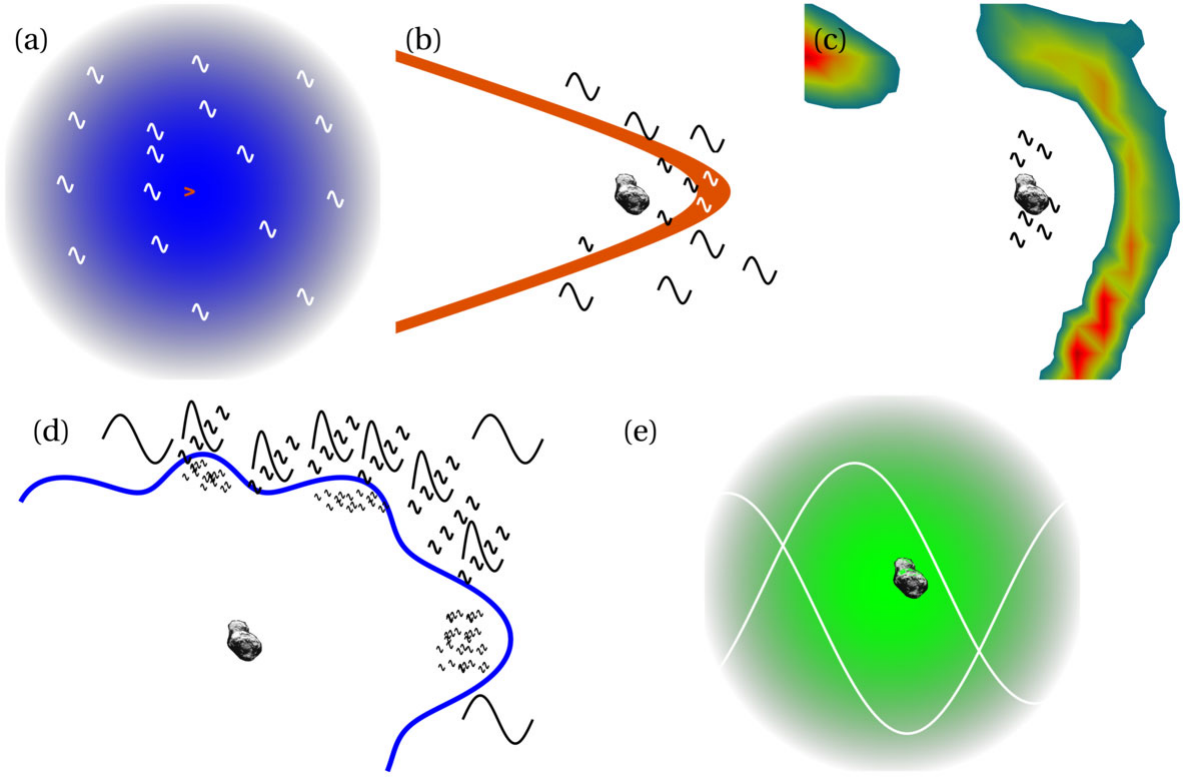
The wave environment of comets on different scales. (**a**) Ion pickup-related waves at distances 1–2 orders of magnitude larger than the bow shock for a comet at high activity. The bow shock is shown in orange in the centre of the panel, and the blue, fading into grey, cloud represents the coma. On this scale, the nucleus is too small to be seen. (**b**) Waves in the foreshock and at the bow shock for a high activity comet. The orange region represents the bow shock. (**c**) Waves in the inner coma, inside of the infant bow shock, at intermediate activity. The infant bow shock is here illustrated through the corresponding proton density structure in a hybrid simulation (see Sect. 4.2 and Gunell et al. 2018). The red parts correspond to the highest density. (**d**) The multitude of waves seen in and around the diamagnetic cavity: ion acoustic waves near the boundary but on the inside; and on the outside lower hybrid waves, steepened waves, and ion Bernstein waves. In addition, the boundary itself may exhibit surface waves. (**e**) Singing comet waves in the coma of a weak activity comet, where the wavelength is on the same order of magnitude as typical length scales of the ionised coma itself, the latter being represented by the green to grey region. From Gunell (2020)

The spacecraft that flew by comets 1P/Halley, 21P/Giacobini-Zinner, and 26P/Grigg-Skjellerup probed a large part of the comet-solar wind interaction regions of their respective targets, but due to the nature of a flyby they could only provide a snapshot of the wave activity and only at perihelion. Rosetta, on the other hand, accompanied comet 67P through the inner solar system, but stayed relatively close to the nucleus throughout the mission. Thus, there is no spacecraft that can provide a complete survey of the wave environment of its target comet, but we can still derive a picture based on the pieces of information that we have from the different comets. Figure 18 illustrates where waves have been observed at comets on large and small scales and for different outgassing rates.

**Pick-up induced waves**. In a very large region around the nucleus (Fig. 18a), one to two orders of magnitude larger than the bow shock, both electrostatic and electromagnetic waves were detected at comets 21P/Giacobini-Zinner and 1P/Halley (Scarf et al. 1986b; Yumoto et al. 1986). These were the result of the interaction between the solar wind and newly formed pickup ions (Wenzel et al. 1986; Scarf et al. 1986a; Scarf 1989). Initially, these pickup ions are practically at rest in the comet frame, due to the low neutral outgassing speed. In the solar wind frame they will therefore, from the start, have velocity components perpendicular and parallel to $\mathbf{B}$. This results in a ring-beam distribution, which is unstable and can lead to various wave modes, like the ion cyclotron wave and the mirror mode (Gary 1991, 1992; Gary et al. 1993). The first in-situ cometary encounters with 1P/Halley, 26P/Grigg-Skjellerup and 21P/Giacobini-Zinner made it possible to investigate the presence of these waves near these medium-to-highly active comets (Motschmann and Glassmeier 1993; Cao et al. 1995; Ip 2004). A full review of instabilities caused by ion pick-up can be found in Wu and Davidson (1972), in Gary and Madland (1988), Gary (1991) and, more recently, in Matteini et al. (2015) for cometary plasmas.

The Giotto magnetometer data (Neubauer et al. 1986) for the flyby of comet 26P/Grigg-Skjellerup were investigated for the presence of ion cyclotron waves (see e.g., Neubauer et al. 1993a; Glassmeier et al. 1993; Glassmeier and Neubauer 1993; Coates et al. 1993; Volwerk et al. 2013). Waves near the $\text{H}_{2}\text{O}^{+}$ cyclotron frequency were found to be weak ($\sim 1~\text{nT}$) and irregular during the inbound pass and strong ($\sim 10~\text{nT}$) “anharmonic” outbound (Glassmeier et al. 1997). The reason for this asymmetry in the growth of the cyclotron waves remains unclear. However, one possibility is that the wave properties could be influenced by asymmetries in the bow shock at comet 26P/Grigg-Skjellerup (see Sect. 4.2). At the other comet visited by the Giotto spacecraft, 1P/Halley, no heavy ion cyclotron waves were found (Tsurutani et al. 1987), however waves with characteristics matching those of ion cyclotron waves were observed at large distances from the nucleus (Yumoto et al. 1986). At comet 67P/Churyumov-Gerasimenko ion cyclotron waves have not been found. This might be due to the high plasma-$\beta $ in the inner coma, which suppresses the growth of these waves. The related mirror mode, however, thrives in high-$\beta $ plasmas, and these structures, and the possibly related magnetic holes, have been found at comets.

**Mirror modes**. At comet 67P, evidence was found for two different kinds of mirror modes on 5–6 June 2015. There were mirror mode trains with a size of one to three water-ion gyro radii, which are created by local pick-up of freshly created $\text{H}_{2}\text{O}^{+}$, and another set of trains with a size of 10 to 16 water-ion gyro radii, created by $\text{H}_{2}\text{O}^{+}$ pick-up in the solar wind, and transported towards Rosetta (Volwerk et al. 2016).

Mirror modes are non-propagating structures, but as they are transported by the plasma flow they are embedded in, they tend to diffuse. Hasegawa and Tsurutani (2011) proposed a Bohm-like diffusion (Bohm et al. 1949) for the mirror modes, in which the higher frequencies of the structures diffuse faster than the low frequencies, and thereby the mirror modes get larger as they are transported downstream. Schmid et al. (2014) showed that this diffusion takes place both in the magnetosheaths of Venus and of comet 1P/Halley.

**Magnetic holes** are thought to have evolved from mirror mode waves and are identified as distinct depressions in the magnetic field embedded in average plasma conditions (Winterhalter et al. 1995). They have been identified near comet 67P (Plaschke et al. 2018), albeit at a rather low rate of 23 holes in 2 months (April and May 2015), i.e. $\sim 0.4$ per day. Interestingly, magnetic holes were observed by Rosetta both in and out of the solar wind ion cavity (Sect. 4.3). At first the holes showed the characteristics of those in the solar wind, e.g., with an ion density maximum at the centre of the hole. However, after the spacecraft entered the solar wind ion cavity in April 2015, the characteristics of the holes seemed to change, with observations of density maxima shifted with respect to the magnetic decrease. Sometimes, the magnetic structure also changed to a more complex form than a simple dip. This may indicate that the holes were interacting with the cometary ions, as they became of comparable size to the heavy pick-up ion gyro radii.

**Waves upstream**. At a high activity comet, outside the bow shock, there is a foreshock region that exhibits phenomena not unlike those at the bow shock of a planet, see Fig. 18b. In the foreshock, wave spectra indicative of electron plasma oscillations, whistler mode waves, as well as ion acoustic waves were detected at comets 21P/Giacobini-Zinner (Fuselier et al. 1986) and 1P/Halley (Oya et al. 1986). Fuselier et al. (1986) also detected enhanced levels of field-aligned electron heat flux, which they could backtrace to the bow shock region of comet 21P/Giacobini-Zinner. In the bow shock region of comet 21P/Giacobini-Zinner, ion acoustic waves and electron plasma oscillations were observed on the upstream side, and on the downstream side lower hybrid waves and also whistler mode waves were seen (Scarf 1989).

The Rosetta spacecraft spent its time close to the nucleus of comet 67P, and thus it was never in a place where it could detect the kind of large-scale waves described above for comets 1P/Halley and 21P/Giacobini-Zinner. Instead there have been a few observations of ion acoustic waves (e.g. Gunell et al. 2017b), lower hybrid waves (e.g. Karlsson et al. 2017), “steepened” waves of yet undetermined origin (Stenberg Wieser et al. 2017; Ostaszewski et al. 2021), and singing comet waves (e.g. Richter et al. 2015).

**Ion acoustic waves** are electrostatic waves in a plasma on ion timescales, that is to say, for frequencies below the ion plasma frequency. The purely ion acoustic mode exists in unmagnetised plasmas and for propagation directions parallel to the magnetic field in magnetised plasmas. These waves are weakly damped only when the electrons are much warmer than the ions, $T_{\mathrm{e}}\gg T_{\mathrm{i}}$. In this limit and for long wavelengths, their phase speed is given by the ion acoustic speed $c_{\mathrm{s}}=\sqrt{k_{\mathrm{B}}T_{\mathrm{e}}/m_{\mathrm{i}}}$ (see for example Krall and Trivelpiece 1973). The plasma in the inner coma of comet 67P fulfils these conditions. The majority of the ions are water-group ions with a very low temperature, and the electrons have temperatures in a range $5~\text{eV}\lesssim k_{\mathrm{B}}T_{\mathrm{e}}\lesssim 10~\text{eV}$ (Eriksson et al. 2017). Also, at comet 67P the plasma can often be seen as unmagnetised from a wave theory point of view even if the magnetic field is not exactly zero, because the plasma frequency is much higher than the cyclotron frequency and the wavelengths of ion acoustic waves are in most cases much less than the gyroradii of ions and electrons.

**Fig. 19:**
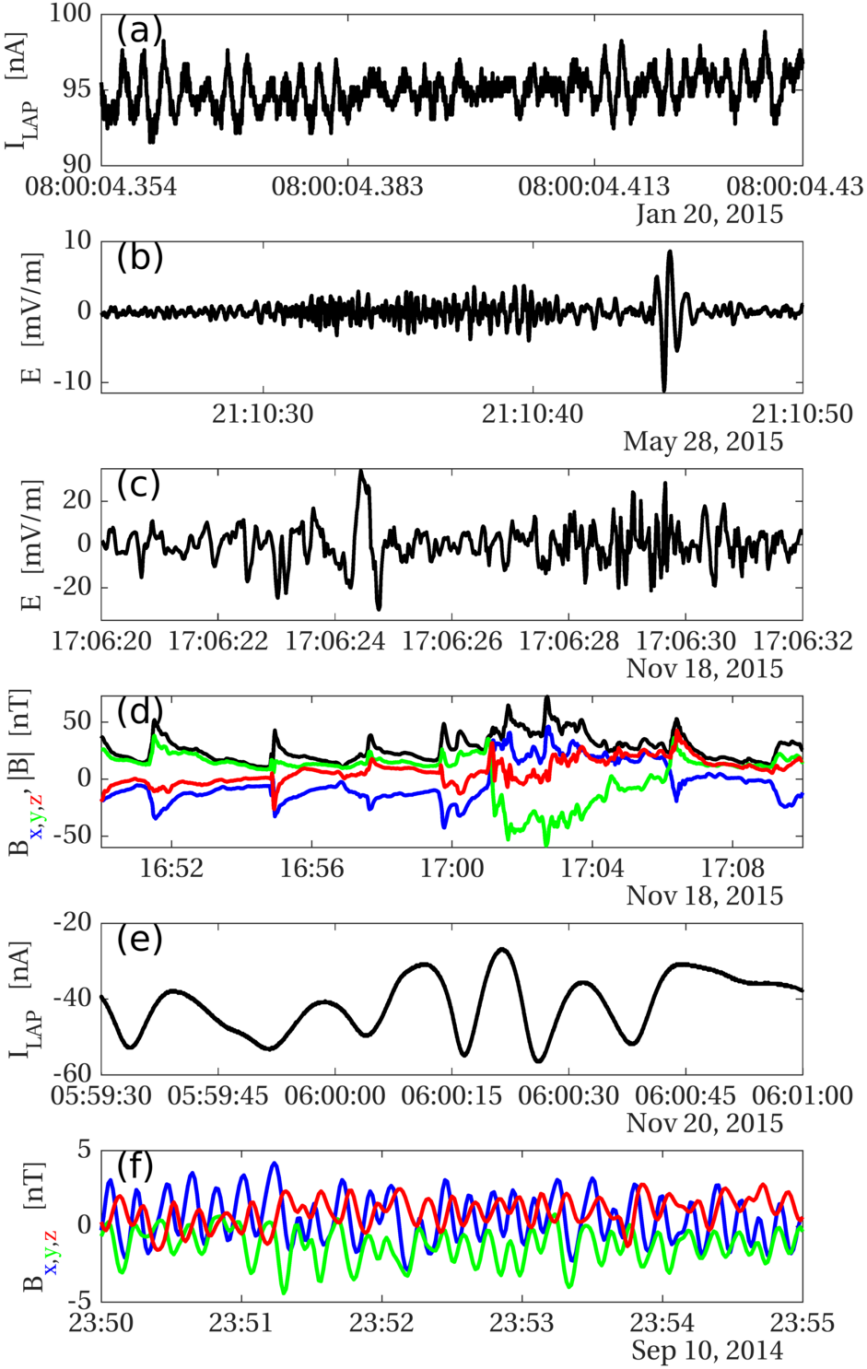
Time series of waves observed by the Rosetta spacecraft during its mission. (**a**) Ion acoustic waves 28 km from the centre of the comet, which was at 2.5 AU heliocentric distance (power spectral densities were published by Gunell et al. 2017b). (**b**) Low frequency ion acoustic waves in the diamagnetic cavity at the same time as waves in the same frequency range were seen outside the cavity (both reported by Madsen et al. 2018). (**c**) Electric field observations of lower hybrid waves during a part of the interval shown in panel (d) (interval selected from André et al. 2017). (**d**) Steepened waves seen outside the diamagnetic cavity in the magnetic field components as well as the magnitude of the magnetic field. (**e**) Langmuir probe current showing waves interpreted as ion Bernstein waves (Odelstad et al. 2020). (**f**) Singing comet waves shortly after the spacecraft arrived at comet 67P (originally reported by Richter et al. 2015)

Gunell et al. (2017b) reported ion acoustic waves like the ones shown in Fig. 19a at approximately 500 Hz observed by RPC-LAP on the 20 January 2015 at a cometocentric distance of $28~\text{km}$. The shape and structure of the ionised coma resemble what is shown in Fig. 18c. At this point in the cometary environment’s development heated proton populations were detectable. However, these protons had only a negligible influence on the ion acoustic waves as kinetic dispersion relation calculations showed (Gunell et al. 2017b). Instead the observations were interpreted in terms of the interaction between the cold cometary ions and a beamlike population of cometary ions that had been accelerated by the convectional electric field. The neutral gas density was seen to modulate the wave power in a way which was consistent with the ion temperature being increased by the neutrals scattering the beamlike ion population. Ion acoustic waves were also observed during the close flyby on 28 March 2015, when Rosetta passed the nucleus at 15 km cometocentric distance (Gunell et al. 2021). The authors interpreted the observations in terms of a current-driven instability, where the current is associated with the draping observed by Koenders et al. (2016a).

**Langmuir waves**. Myllys et al. (2021) reported the observation of higher frequency electrostatic waves, associated to the electron dynamics, namely Langmuir waves, observed by RPC-MIP during the whole mission, mostly in the 100 kHz to MHz regime, corresponding to the local plasma frequency. The Langmuir wave activity monitored by RPC-MIP was significantly enhanced during SIR or CME interactions with the comet, which could be the signature of suprathermal electron beam associated to such energetic interplanetary events. It was also shown that the Langmuir wave activity is strongly enhanced near perihelion.

**Fig. 20:**
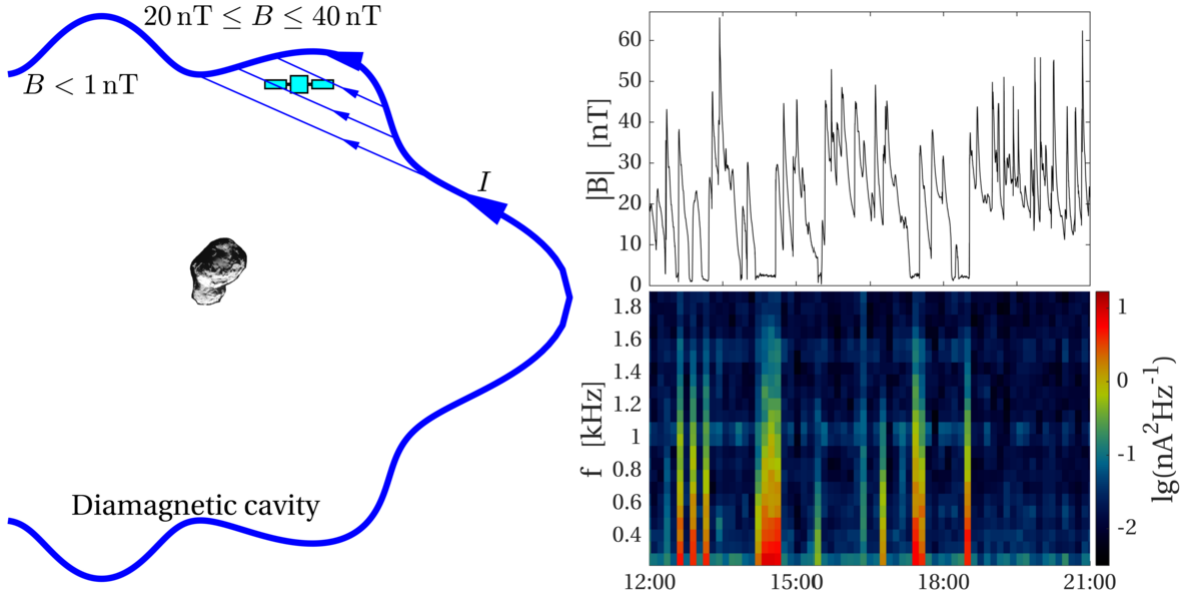
Wave observations when Rosetta was in the diamagnetic cavity. The power spectral density of the waves was measured using the Langmuir probe instrument, RPC-LAP, on 3 August 2015. From (Gunell 2020)

**Waves at the diamagnetic cavity**. When comet 67P was at perihelion further ion acoustic wave observations were made while Rosetta was in the diamagnetic cavity. The wave spectrum peaked around 200 Hz and the wave power decreased at higher frequencies, vanishing in the noise floor around the ion plasma frequency at 1.5 kHz (Gunell et al. 2017a). An example from 3 August 2015 is shown in Fig. 20.

The waves almost exclusively appeared inside the diamagnetic cavity. When the spacecraft was outside the cavity the waves were much diminished, if they could be detected at all. The diamagnetic cavity itself is described in more detail in Sect. 4.6. The explanation by Gunell et al. (2017a) was that since the spacecraft velocity is negligible, the diamagnetic cavity encounters are caused by bulges on the diamagnetic cavity boundary moving past the spacecraft as illustrated in Fig. 20. A current must flow at the boundary to maintain the difference in magnetic field between the inside and the outside of the boundary. If this difference is not constant along the boundary some of the current closes through the bulges, exciting current-driven ion acoustic waves. Calculations, using kinetic theory, showed as an example that a current density of $51~\text{nA}\,\text{m}^{-2}$ would lead to growing ion acoustic waves, while keeping the magnetic flux density within the observational constraint that $B\leq 1~\text{nT}$, provided that the current-carrying layer is no more than $37~\text{km}$ wide (Gunell et al. 2017a). Examining a much lower frequency range of only a few Hz with the aid of the dual Langmuir probe mode of RPC-LAP, Madsen et al. (2018) found ion acoustic waves (see Fig. 19b) in the cavity at the same frequency as that of lower hybrid waves outside the cavity, where the electrons are magnetised. This suggests the possibility of mode conversion at the boundary of the diamagnetic cavity. Further observations, preferably supported by simulations, are needed to identify the drivers of such a mode conversion and to charter the paths of energy transfer in the boundary region.

A multitude of waves, not only those mentioned above, appear near the diamagnetic cavity as illustrated in Fig. 18d. Both André et al. (2017) and Karlsson et al. (2017) used the electric field mode of RPC-LAP to observe lower hybrid waves, $f\lesssim 15~\text{Hz}$, in the plasma outside the diamagnetic cavity of comet 67P (see Fig. 19c). These lower hybrid waves appear in bursts that coincide with sharp density gradients being present in the plasma at the spacecraft position. The gradients were found to be sufficient for the lower hybrid drift instability to cause waves to grow in the frequency range where the waves were observed. It was found that both the density, the water ion energy, and the magnitude of the magnetic field increases over approximately 20 s followed by a relaxation period of 1–5 min (Stenberg Wieser et al. 2017). These minute-timescale oscillations are known as *steepened waves* (see also the diamagnetic cavity Sect. 4.6). As can be seen in Fig. 19d the magnetic field pattern of the steepened waves is quite distinctive and was detected already during the first encounter of the diamagnetic cavity (Goetz et al. 2016b). Both magnetic field and ion signatures of steepened waves can be seen in Fig. 42 in Sect. 4.6. The cause of the steepened waves is yet unknown. Another wave was found at comet 67P by Odelstad et al. (2020), who detected large-amplitude density oscillations at frequencies near 0.1 Hz in a region outside the diamagnetic cavity boundary. The density oscillations were, in some but not all of the observed cases, accompanied by magnetic field oscillations phase shifted by $\sim90^{\circ}$. Odelstad et al. (2020) interpreted the observations as Ion Bernstein waves (see Fig. 19e).

**Singing Comet waves**. Soon after Rosetta arrived at comet 67P, magnetic field oscillations, as illustrated in Fig. 19f, were detected at frequencies below 100 mHz with a peak around 40 mHz (Richter et al. 2015). These waves were called *singing comet* waves, and using the magnetometers aboard both the Rosetta orbiter and the Philae lander the wavelength was estimated to be in an approximate range of $100\text{--}700~\text{km}$ (Richter et al. 2016). This means that the wavelength is on the same order of magnitude as typical length scales of the ionised coma itself during the comet’s low activity phase as illustrated in Fig. 18e. Breuillard et al. (2019) combined magnetic field measurements with density measurements to show that the singing comet waves are compressional, and Goetz et al. (2020b) found these waves extending to distances of $800~\text{km}$ from the nucleus during Rosetta’s tailward excursion in 2016. An analytic three-fluid model of a modified ion Weibel instability was used to explain the generation of the singing comet waves (Weibel 1959; Meier et al. 2016). There is partial agreement between observations and model results (Goetz et al. 2020b). However, as a uniform plasma was assumed in the model and the cometary plasma is not uniform – the plasma density near the nucleus changes significantly in a fraction of the wavelength – one cannot expect it to be a perfect description of the plasma. Kinetic and space charge effects are also beyond the simple model and may have an impact on the generation of singing comet waves.

The solar wind-comet interaction, through the pick up of the newly created ions leads to a large energy input into the plasma and the ion scale. This energy partly goes into the creation of waves and fluctuations. However, in part it also diffuses to smaller scales through a turbulent cascade (see also Tsurutani et al. 2018).

In the case that the IMF is more aligned with the solar wind flow, such that an anisotropy of $T_{\|} \gg T_{\perp}$ is created, right-hand polarized waves can be generated, observed, e.g., at comet 21P/Giacobini-Zinner (Tsurutani and Smith 1986a,b). At three different highly active comets (mentioned above) energy is pumped into the magnetic field at the $\text{H}_{2}\text{O}^{+}$ gyrofrequency, and the power cascades to smaller scales, i.e., higher frequencies with a power law around $f^{-2.0}$, which would be according to Kraichnan turbulence (Kraichnan 1965).

**Fig. 21:**
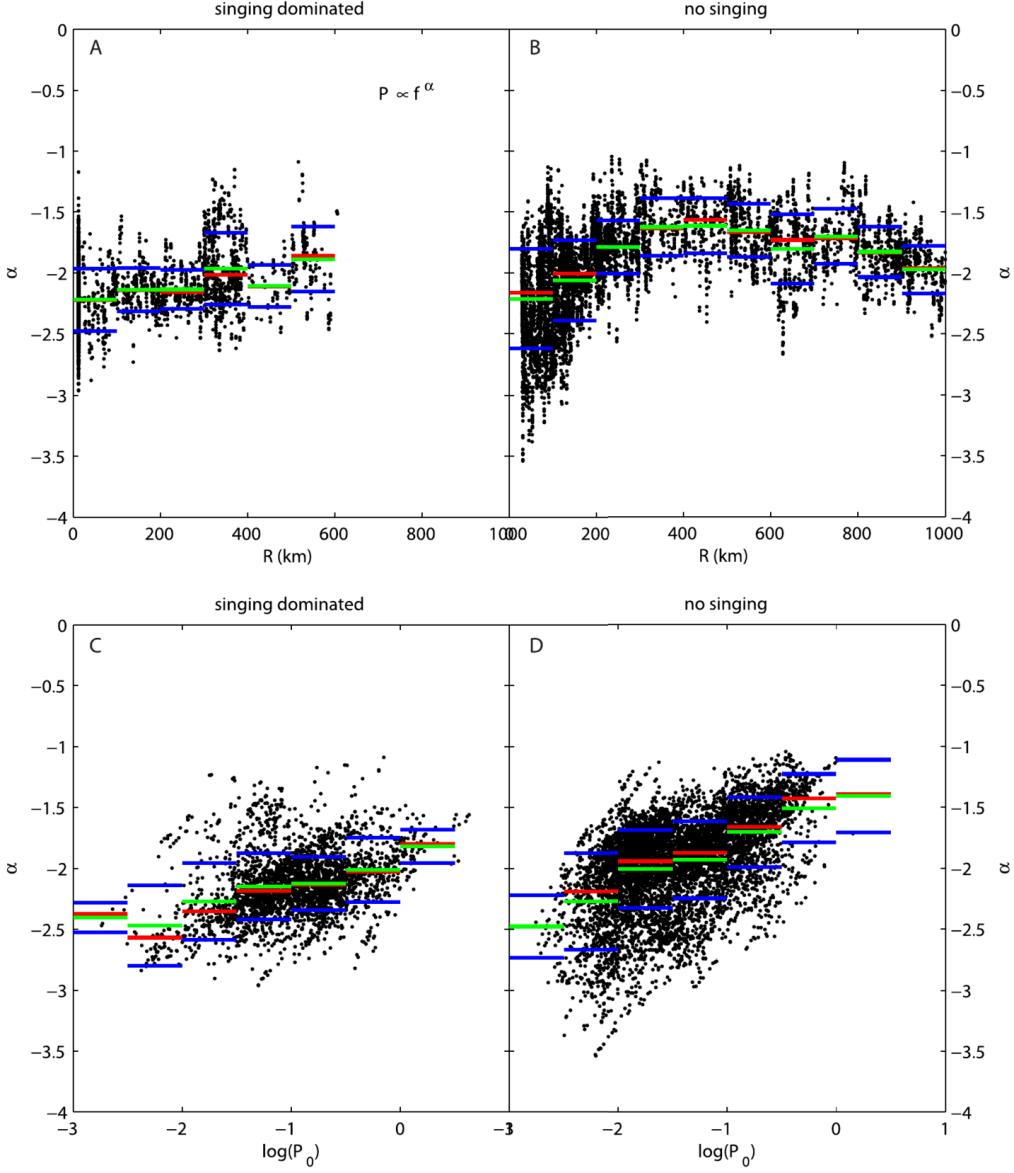
**A**–**B**: The spectral index $\alpha $ as a function of radial distance from the comet for the singing dominated intervals and the no singing intervals. **C**–**D**: The spectral index $\alpha $ as a function of $\log (P_{0})$, from the power law fittings. In all panels the median (red), mean (green), and standard deviation (blue) of the $\alpha $-distributions are plotted. From (Volwerk et al. 2018), reproduced with permission ©ESO

During the Rosetta tail excursion, and taking advantage of the long dwelling time of the spacecraft, Volwerk et al. (2018) determined the power spectral slope of the magnetic field by an analytic fit, $P_{fl}^{fh}(f) = P_{0} f^{\alpha}$, to the power spectral density. The spectral index $\alpha $ is shown in Fig. 21 as a function of both cometocentric distance and of $\log (P_{0})$ and separated into periods with and without singing comet waves. There is a large spread in $\alpha $, but the mean/median spectral slope is around $\alpha \approx -2$ over the full distance travelled by Rosetta. However, when the spectral slope is plotted as a function of $\log (P_{0})$ there is a significant change in $\alpha $ from $\alpha \approx -2.5$ to $\alpha \approx -1.5$ for the non-singing events. This flattening of the spectrum for stronger input $P_{0}$ is expected, as more power at large scales leads to more cascade to smaller scales. However, the diffusion at the smallest scale will not increase with more input, and thereby the total power over the frequency range will increase.

### Response of Plasma Environment to Solar and Cometary Transients

There are two types of transients that modify the plasma around comet 67P: external ones such as flares, CIRs, and ICMEs (see Sect. 2.1.2) and internal ones such as outbursts. The effects of these triggers can be quite different depending on the heliocentric distance of the comet, the location of Rosetta within the coma and the strength of the transient.

#### External Transients

**Fig. 22:**
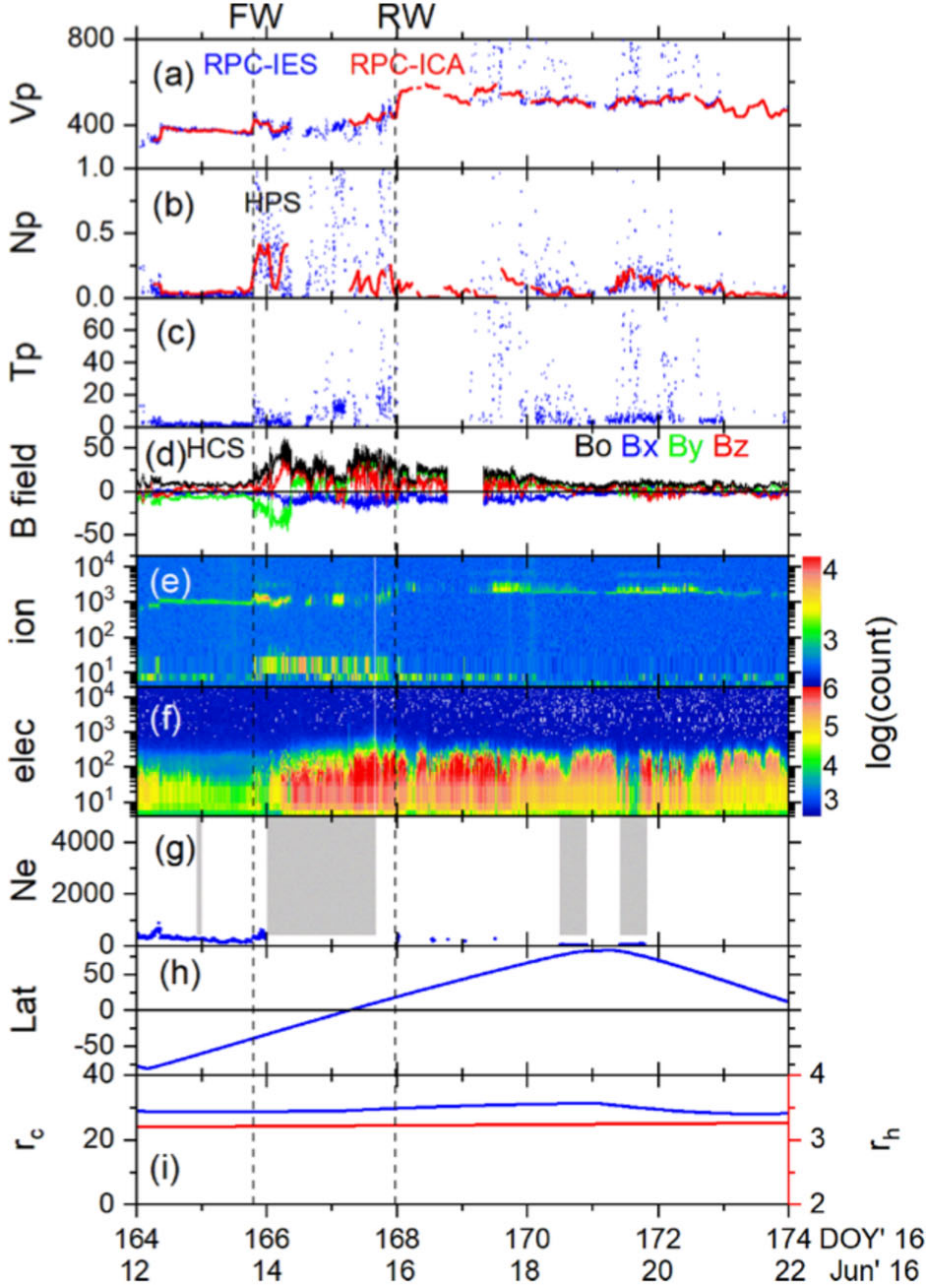
Example of the cometary plasma response to a CIR as measured by the RPC instruments. **a**) Proton velocity in km/s, **b**) proton density in $\text{cm}^{-3}$, **c**) proton temperature in eV, **d**) magnetic field in nT, **e**) and **f**) ion and electron energy spectra with energy in eV, **g**) electron density in $\text{cm}^{-3}$, **h**) latitude of the spacecraft in degrees, **i**) cometocentric (blue) and heliocentric (red) distance of the spacecraftin km. From Hajra et al. (2018a), Fig. 3

**Corotating Interaction Regions**. The impact of CIRs on the cometary plasma has been studied for two intervals: October 2014 to December 2014 and June 2016 to September 2016 (Edberg et al. 2016b; Hajra et al. 2018a). These intervals have the advantage that the solar wind ions still penetrated the coma and could be used to identify the characteristic signatures of CIRs. For both intervals the gas production rate was below $10^{26}~\text{s}^{-1}$. A typical example is given in Fig. 22. Although each CIR impact is different in the details, generally the plasma at the comet during a CIR impact is characterised by increases in proton density, velocity and temperature, as well as in magnetic field strength, suprathermal ($12\text{--}200~\text{eV}$) electron flux and cometary plasma density. This increase depends on the location of Rosetta and on the characteristics of the CIR. For example Hajra et al. (2018a) found that the increase in plasma density over the southern (summer) hemisphere is much stronger than over the northern (winter) hemisphere (see also Sect. 3.2.1). However, when the increase is normalized to quiet-time values these differences tend to vanish. This indicates that the plasma density increase is heavily dependent on the neutral gas density at the measurement location. Effects of the CIR usually last around one day, and the signatures of a forward and reverse wave are clearly visible even in the coma.

While first speculations attributed the large increase in plasma density to either compression, additional ionisation or acceleration due to the ion pick-up process, it was later found that an increase in the suprathermal electron flux in turn leads to a significant increase in the electron impact ionisation rate (Galand et al. 2016; Heritier et al. 2018; Hajra et al. 2018a). Thus, the dominant process behind the overall increase in plasma density is electron impact ionization which produces the bulk of the plasma. The increase in proton density (the minor contributor to overall density) and magnetic field strength are mostly attributable to magnetic compression.

The sudden change of plasma properties around the comet can also move boundaries or cause boundaries to form. One such example was shown in Edberg et al. (2016b), where a region with very warm protons, increased magnetic field turbulence and higher electron fluxes was briefly observed. This boundary was later identified as the infant bow shock (Gunell et al. 2018; Goetz et al. 2020a), see also Sect. 4.2.

While CIRs impact on a comet are now understood in the case of a low outgassing activity that does not prohibit solar wind ions from penetrating the inner coma (Edberg et al. 2016b; Hajra et al. 2018a), the understanding of CIRs behavior within the so-called solar wind ion cavity, observed for strong enough outgassing rates to form such a cavity, is still lacking.

**Interplanetary Coronal Mass Ejections**. As with CIR impacts, the reaction of the cometary plasma to ICMEs varies with each event. Five events have been studied in detail at 67P so far, whose common denominators regarding the plasma response are: (i) increase in magnetic field and plasma density, (ii) increase in flux of energetic electrons, and (iii) a Forbush decrease in the radiation environment. During the low activity phase, in October 2014, an ICME was tracked throughout the solar system, using data at multiple planets (including Mars and Saturn), combined with solar observations and Rosetta data at 67P (Witasse et al. 2017). The ICME signature showed the typical increases as well as an increase in the measured proton speed. This event and the two described by McKenna-Lawlor et al. (2016) are the only ones during which it was possible to observe the solar wind in-situ during a CME, as the other events impacted the comet when Rosetta was in the inner coma. For the October 2014 event, the increase in plasma density occurred in two separate increases, indicating that a shock and ejecta cloud formed the impact ICME, which led to first a small increase in density (shock impact) and then a larger increase (impact of ejecta). Using multiple spacecraft it was possible to constrain the ICME speed to $550~\text{km/s}$ at $3.1~\text{AU}$ and a slowly decreasing velocity at higher heliocentric distances.

Another ICME impact was detected in July 2015, very close to perihelion when Rosetta was only $170~\text{km}$ from the nucleus. The compression of the magnetic field produced field strengths of about $300~\text{nT}$, the highest value observed at the comet during the entire Rosetta mission (Goetz et al. 2019). As with the earlier ICME event the increase in magnetic field and density occurred in two steps. It was speculated that this was due to the ICME structure. The density increase could also be due to the magnetic field connecting the measurement location to different locations in the near-coma environment. Both explanations fit the observations and thus no conclusion could be drawn. The same goes for the electron flux and magnetic field turbulence, which were observed to increase for about half an hour before decreasing and then increasing again. The authors also raised the possibility of a CIR as trigger for the plasma changes, but the Forbush decrease visible in the radiation monitor data indicated that an ICME must have played the more significant role in this event.

**Fig. 23:**
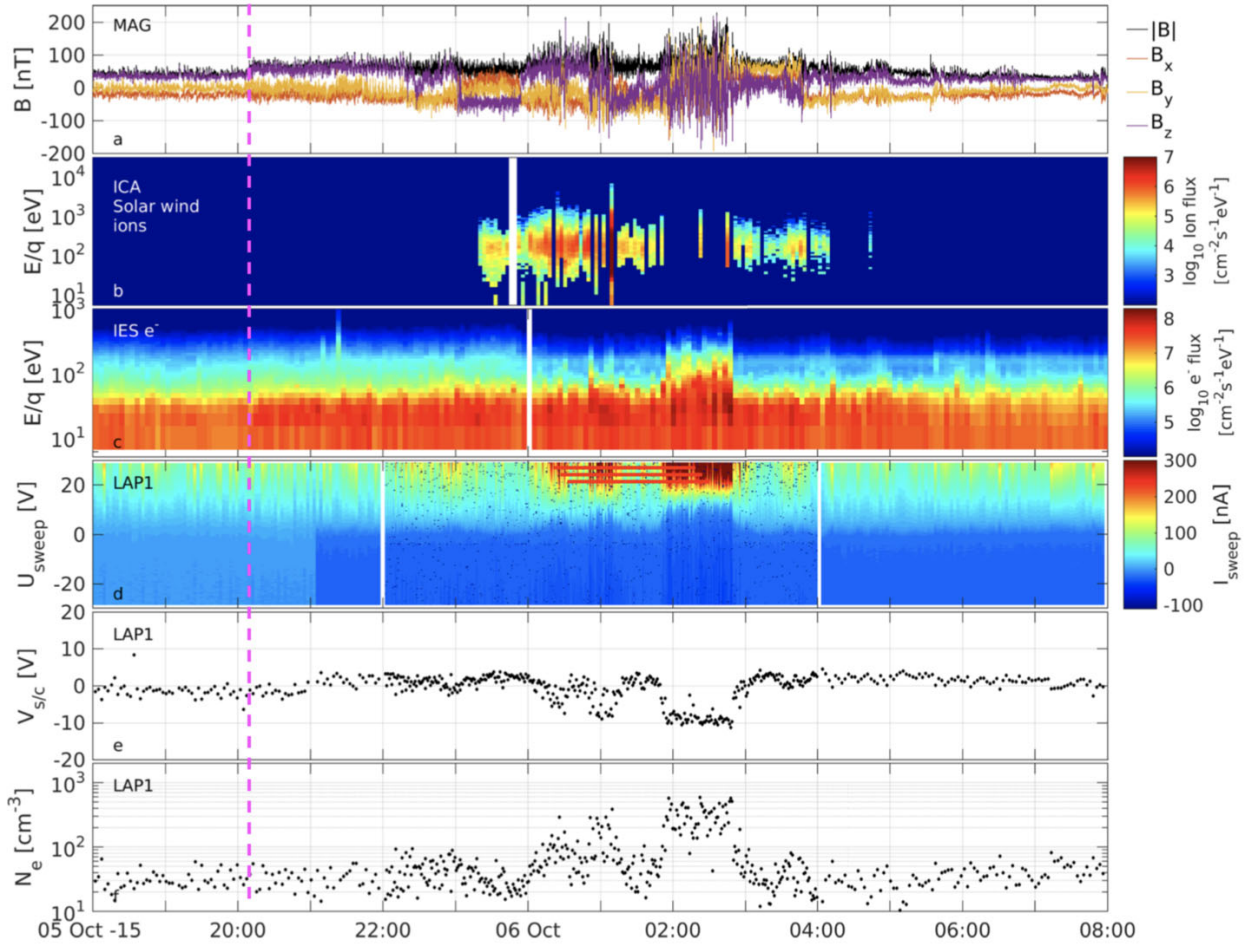
Measurements during the October 2015 ICME impact. From top to bottom: **a**) magnetic field components in CSEQ and magnitude, **b**) solar wind ion flux, **c**) electron flux, **d**) Langmuir probe current-voltage sweep, **e**) spacecraft potential, **f**) plasma density. The start of the ICME impact is marked as a vertical dashed line. From Edberg et al. (2016a), Fig. 4

A third event took place during the dayside excursion in October 2015, when Rosetta was at a cometocentric distance of $800~\text{km}$ (much larger than in the previous case) (Edberg et al. 2016a). Before the ICME arrived at 67P, Rosetta was located inside the solar wind ion cavity, but the additional pressure from the high speed solar wind managed to push the solar wind ions closer to the nucleus, so that they were observable at the spacecraft location. The protons were still highly deflected and decelerated. The other signatures were very similar to the previously studied events. Large variations in the magnetic field were identified as flux ropes, which could be the result of reconnection in the inner coma. However, no flux rope signatures could be found in the July ICME impact. This is surprising as the spacecraft was in the inner coma during the July event, where according to Edberg et al. (2016a) the flux ropes should be generated. Figure 23 illustrates the October 2015 ICME impact. The observations of the plasma changes are very similar to the July event. Fortunately, this event also proved to be visible with the ALICE instrument, which showed that the electron impact ionisation contribution was significant due to the higher flux of suprathermal electrons (Noonan et al. 2018, see also Sect. 2.5).

**Flares**. Flares are short-lived increases in the Sun’s EUV and X-ray emissions originating in sunspots and propagating through the entire solar system at light speed. Because of the higher EUV intensity, flares are expected to increase the amount of photoionisation in planetary and cometary atmospheres. Edberg et al. (2019b) investigated a total of 1504 flares directed at 67P and their impact on the plasma environment at 67P. Against expectation only very few flares coincided with an increase in the plasma density. This was attributed to two effects: (i) at low gas production rate, photoionisation is a minor process in the creation of the plasma, thus an increase in EUV does not contribute much to the plasma source term; (ii) at high gas production rates, the plasma at 67P is very dynamic with large density variations and, consequently, the impact of a flare is drowned by the background plasma changes.

#### Internal Transients

During the Rosetta mission, at least one cometary outburst was observed to have a significant impact on the plasma at Rosetta’s location: it took place on 19 February 2016 and was first described by Grün et al. (2016). This outburst occurred close to the sub-spacecraft point on the comet and thus the increase in brightness was followed by a pronounced increase (factor 1.8) in the measured neutral gas density. As is typical, the plasma density also increased with the neutral gas density, but the increase in plasma density did not scale with the increase in neutral density: it was significantly more pronounced (factor 3.8). This over-increase was attributed to the additional effect of electron impact ionisation, which is usually less important at large heliocentric distance, but may become more significant due to the larger neutral densities. In addition, it is suggested that the more efficient ion-neutral coupling in the denser neutral gas reduced the ion speed and thus the ionisation length scale (Hajra et al. 2017). During the outburst, the suprathermal electron flux decreased, and the solar wind ions vanished from the instruments’ field of view. The magnetic field increased and changed directions as a result of local draping, and the intensity of the singing comet waves (see Sect. 2.3.7) that are typical for this stage of activity strongly decreased. Breuillard et al. (2019) studied this interval further and found that the waves did not actually vanish, but rather that their frequency was shifted to lower values. They speculated that this was related to the additional ion-neutral drag and the thus changing Doppler shift. For a discussion of the changing emission profile during an outburst see Sect. 2.5.

### Emissions Induced by the Plasma in the Cometary Environment

In addition to directly measuring and characterizing the plasma, emissions can also be used to indirectly investigate the plasma. Electron-neutral collisions in the coma can cause the excitation of neutral species across many energy and wavelength scales. In the far-ultraviolet, (FUV), 1000–2000 Å photons have energies $\sim 10~\text{eV}$, which are typically associated with resonance transitions of cosmically abundant atoms which are the products of the primary molecular species in the coma. Only CO has electronic bands that fluoresce directly in the UV.

The identification of dissociative electron impact excitation in the coma of 67P connected some periods of ultraviolet and visible near-nucleus coma emissions to the properties of the local cometary plasma environment. Charged-particle/neutral interactions had previously been observed in cometary comae in the form of solar-wind charge exchange (Lisse et al. 1996; Cravens 1997; Bodewits et al. 2012), but the Rosetta mission identified electron-neutral interactions in the analysis of UV spectra and optical images of 67P (Feldman et al. 2015; Bodewits et al. 2016). Dissociative excitation is the process by which a neutral molecule is split after collision with an energetic electron or photon, producing excited atomic or molecular fragments. The brightest emission lines observed at comet 67P in the FUV were associated with the atomic transitions of the dissociation products of the major neutral species in the coma (i.e. H, O and C, see Table 3). In addition to these atomic multiplets, electronic bands of CO were observed towards the long-wavelength end of the FUV range. In optical wavelengths, dissociative electron impact produced excited molecules such as OH, $\text{OH}^{+}$, $\text{CO}_{2}^{+}$, and O i. The electron/neutral collision produces a spectrum that is fundamentally different from typical atomic resonance scattering ($h\nu +\text{neutral}$) emissions in the FUV. This aids in the identification of the parent molecule and thus composition of the inner coma.

**Table 3:** Principal atomic and molecular FUV emission features seen in Alice spectra (top) and known electron impact induced emission features identified in OSIRIS narrowband filter images (bottom)

Excited species	Emission feature	Wavelength [Å]
H	Ly-*α*,*β*,*γ*,…	1216, 1026, 973,…
O	O i	1027, 1152, 1304, 1356
C	C i	1561, 1657
C^+^	C ii	1335
CO	$A\,^{1}\Pi $–$X\,^{1}\Sigma ^{+}$(Fourth Positive)	1400–1800
CO	$a\,^{3}\Pi $–$X\,^{1}\Sigma ^{+}$(Cameron)	>1800
OH	$A\,^{2}\Sigma ^{+}$–$X\,^{2}\Pi ^{+}$(0–0)	3086
OH^+^	$A\,^{3}\Pi $–$X\,^{3}\Sigma ^{-}$	3359
$\text{CO}_{2}^{+}$	$\tilde {A}\,^{2}\Pi _{g} $–$X\,^{2}\Pi _{g} $	3884
O i	^1^D–^3^P	6300, 6364

In this section we discuss the physics of the emission mechanisms directly observed by the remote sensing instruments on board *Rosetta* at 67P as a result of plasma-neutral collisions, in particular dissociative electron impact excitation. Since there are no X-ray observations of 67P to identify charge-exchange emissions with solar wind ions we point the reader to relevant work cited above and Sect. 2.2.1. We focus specifically on the plasma-neutral interactions that were unambiguously observed at 67P by the Alice far-ultraviolet (FUV) spectrograph and the OSIRIS camera.

Seen from Earth-orbiting observatories most cometary FUV spectral features are the result of photon excitation, or resonance scattering of the dissociation products of neutral molecules in the outflowing coma. Resonance scattering of a solar photon produces spherically symmetric emission that can be directly related to both the atomic column density and the fluorescence efficiency, or g-factor, for the transition. This is proportional to the transition probability multiplied by the solar photon flux at the corresponding wavelength. The emission feature’s brightness, in Rayleighs, can then be written as;

14 $$ B^{X} = 10^{-6}\ N_{n}\ g_{X} $$

where $N_{n}$ is the column density in units of $\text{cm}^{-2}$ and $g_{X}$ has units of $\text{photons}\,\text{s}^{-1}\,\text{atom}^{-1}$. Modelling the photodissociation of parent molecules (Sect. 2.1.1) allows the parent production rates to be derived. In addition, prompt emission following photodissociation can also contribute to the FUV emissions. While these photo-excited emissions depend only on the solar flux at the comet and not the plasma environment, it is necessary to understand them in order to identify the emissions that arise from electron excitation.

#### Dissociative Electron Impact Emissions

Unlike resonance scattering, dissociative electron impact excitation, as the name implies, dissociates and excites a neutral molecule. The energy of the impacting electron must be enough to break apart the target neutral and impart the remaining energy to raising the energy level of an atomic electron. This reliance on electrons above a certain energy, which is unique to each emission feature, closely relates the appearance of dissociative electron impact emissions to the plasma environment properties. However, the efficiency of this process changes with the increasing energy of the impacting electron, with maximum efficiency for UV transitions typically within $80\text{--}130~\text{eV}$ and declining thereafter. Proper treatment of dissociative electron impact emissions therefore requires careful calculation of the equivalent $g_{X}$ from the equation for resonance scattering. To do so requires knowledge both of the emission cross section, $\sigma (E)$, and the flux of energetic electrons, $J(E,s)$.

The brightness, $B^{X}$, of an emission feature, $X$, driven by electron impact is dependent on the particle flux of energetic electrons along the line of sight $s$, $J(E,s)$ [$\text{cm}^{-2}\,\text{s}^{-1}\,\text{eV}^{-1}$], and the number density of the neutral species, $n_{l}(s)$ [$\text{cm}^{-3}$], along the observed line of sight. If the excited states leading to emission are short-lived, the emission brightness is given by

15 $$ B^{X} = 10^{-6}\sum \limits _{l} \int \limits _{\text{S}}\int _{E = E_{Th, l}}^{\infty} n_{l}(s)\, J(E,s)\, \sigma _{l}^{X}(E)\ \mathrm{d}E \, \mathrm{d}s \, [{\mathrm{Rayleigh}}] $$

where the spatial integral is over the column along the line of sight. The contributions from each major neutral species in the coma are summed to give the total brightness originating from dissociative excitation. Because the energies of the impacting electrons are not quantized this process is capable of producing atoms in classically forbidden states, which quickly release a “forbidden” photon.

The emission brightness caused by electron impact is not dependent on the total electron density, but rather the density of electrons which exceed the emission threshold energy, $E_{Th}$. Electrons with energies below the threshold will not contribute as the emission cross section $\sigma _{l}^{X}(E) = 0$ in this energy range.

If the energetic electron flux is constant along the line of sight, Eq. (15) can be simplified to

16 $$ B^{X} = 10^{-6}\sum \limits _{l} N_{l}\ \nu _{l}^{X} $$

where $N_{l}$ is the column density of the neutral species, $l$, and $\nu _{l}^{X}$ is the emission frequency of the species in the given emission feature. In this form $\nu _{l}^{X}$ is equivalent to the $g$-factor described in Eq. (14). This assumption can be made at large heliocentric distances where electron-neutral collisions are uncommon and therefore there is no significant energy degradation of electrons along the line of sight. Chaufray et al. (2017) find that the measurements imply that there must have been a constant suprathermal flux throughout the neutral column when the outgassing rate was low ($Q<10^{26}~\text{s}^{-1}$).

These emissions were most commonly captured in the Alice UV spectrograph in near nucleus coma observations, easily identified by the presence of the classically forbidden O i 1356 Å emission feature, typically associated with aurora (cf. Meng and Huffman 1984). The dissociative electron emissions were pervasive, and were present not only in periods of relative quiet (Chaufray et al. 2017; Feldman et al. 2017; Galand et al. 2020; Stephenson et al. 2021) but in rather unique situations like outbursts (Feldman et al. 2016), co-rotating interaction region impacts (Feldman et al. 2015; Stephenson et al. 2021), and coronal mass ejection impacts (Noonan et al. 2018). Early in the *Rosetta* mission at large heliocentric distances the OSIRIS instrument observed excess emission in gas filter images that could not be accounted for with typical photodissociation, and dissociative electron impact was invoked as a plausible explanation (Bodewits et al. 2016).

The most powerful diagnostics from dissociative electron impact emissions are the intensity ratios of atomic emissions that allow determination of relative molecular abundances. These intensity ratios are measured for different molecules in laboratory experiments (see McConkey et al. 2008, for a review), providing a template for spectral analysis. As an example, the atomic line ratios between Lyman-$\beta $, O i 1304, and O i 1356 are near to 7:2:1 for H_2_O (Makarov et al. 2004). For CO and CO_2_ the line ratios of atomic carbon features at C i 1561 and C i 1657 Å are key for identifying the dominant neutral source; a CO_2_-rich column will produce a C i 1657:1561 ratio of 2:1, while a CO-rich column will produce a C i 1657:1561 ratio of 1:2 (Mumma et al. 1972; Ajello 1971a,b; Ajello et al. 2019). Examples of these signatures are shown in Sect. 2.5.3.

The dissociative electron impact results from UV spectra taken at 67P rely heavily on emission cross sections for the dominant neutrals in the coma: H_2_O, CO_2_, CO, and O_2_. These cross sections, which detail emission rate as a function of electron energy, are often taken with low energy resolutions or with only a few energy settings. Analysing electron impact spectra then requires assumptions about emission line ratio behaviour at different electron energies to initiate models. The need for improved dissociative electron impact emission cross sections to further our understanding of the link between the plasma and neutral environment of 67P is a clear area for future work (Sect. 3.1.3).

#### Ion-Electron Dissociative Recombination

In addition to excitation by energetic electrons, thermal electrons, through dissociative recombination with molecular ions (Sect. 2.2.2), can also leave the resultant atomic or molecular fragment in an excited state leading to visible and FUV emissions that are dependent on the electron temperature. Laboratory studies of electron recombination with $\text{H}_{2}\text{O}^{+}$ (Sonnenfroh et al. 1993), $\text{H}_{3}\text{O}^{+}$ (Gougousi et al. 1997), and $\text{CO}_{2}^{+}$ provide signatures that allow the identification of these processes from the observed spectra. In the case of $\text{CO}_{2}^{+}$, both $a\,^{3}\Pi $, the upper state of the Cameron band system (Skrzypkowski et al. 1998), and $A\,^{1}\Pi $, the upper state of the Fourth Positive band system (Gutcheck and Zipf 1973; Tsuji et al. 1995), are populated. The latter is particularly relevant as the CO Fourth Positive bands are strongly present in Alice spectra when CO_2_ is abundant in the coma (Feldman et al. 2018), produced by energetic electron impact on CO_2_ (Ajello et al. 2019), or solar fluorescence of CO (Lupu et al. 2007). However, as Tsuji et al. (1995) demonstrate, from energy considerations dissociative recombination of $\text{CO}_{2}^{+}$ in its lowest vibrational state, only $v = 0$ and 1 of the CO $A\,^{1}\Pi $ state will be populated, giving rise to a Fourth Positive band spectrum sharply truncated below 1500 Å. This does not appear to be the case in any spectrum in the Alice database (see Sect. 2.5.3), suggesting that this mechanism does not play a major role in the inner coma. This absence in the Alice spectra is in agreement with findings from the plasma instruments on board. The ion-electron recombination timescale was much longer than the transport timescale for the majority of the escort phase; for $Q_{0}<10^{28}\,\text{s}^{-1}$ recombination is not a major ion loss process (Beth et al. 2019). Further analysis, using RPC ion and electron measurements, is warranted to support this conclusion.

#### Observations

The far-ultraviolet bandpass of the Alice spectrograph is 750–2050 Å with a spectral resolution of $\sim10$ Å for a filled slit. Alice observed atomic and molecular emissions excited by dissociative electron impact in the inner coma for a range of cometary activity levels and composition. The excited species were identified by comparison of laboratory electron impact emission cross-sections for H_2_O, CO, and CO_2_, (Makarov et al. 2004; Mumma et al. 1972; Ajello 1971a,b; Ajello et al. 2019) to the Alice spectra.

**Fig. 24:**
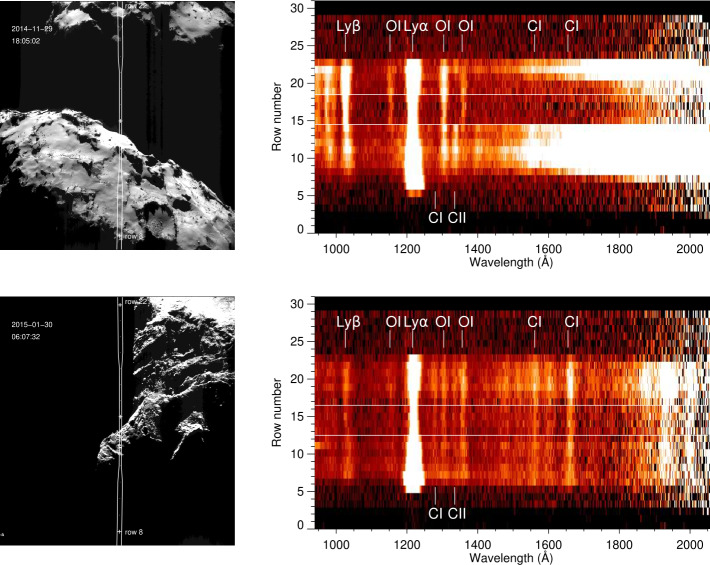
NAVCAM context images (left) with the Alice slit superimposed and near simultaneous spectral images (right) for 2014 November 29 (top) and 2015 January 30 (bottom). The white horizontal lines outline the 4 rows used in the spectral extraction. In all of the images the Sun is towards the top. From Feldman et al. (2018), ©AAS. Reproduced with permission

Alice spectra taken against the shadowed nucleus of the comet provide stronger constraints on the electron and neutral densities along the line-of-sight to the nucleus and also avoid contamination from the extended coma and interplanetary background. Examples of this geometry are shown in the NAVCAM (navigation camera) images of Fig. 24, together with their corresponding two-dimensional FUV spectral images. In addition, since Alice is an imaging spectrograph, the variation in brightness along the slit also provides a measure of the distribution of the energetic electrons producing the emission.

The first identification of dissociative electron impact emissions were made from observations in September and October 2014 (Feldman et al. 2015). The latter were limb observations from a distance of 10 km giving a spatial resolution (2 pixels) of $\sim100~\text{m}$ along the slit, which was positioned roughly perpendicular to the limb. These observations showed that the emissions decreased outward from the limb and were concentrated in a region $\sim1~\text{km}$ above the limb. A robust multi-instrument analysis of the FUV emissions is described in Sect. 3.2.2 and by Galand et al. (2020) and Stephenson et al. (2021).

**Fig. 25:**
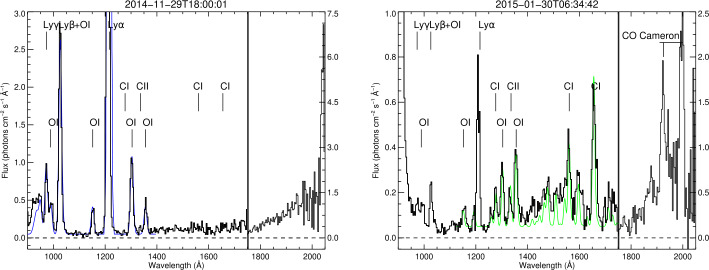
Coma spectra corresponding to the spectral images in Fig. 24. All of the spectra are summations over 4 rows ($0.05^{\circ}\times1.2^{\circ}$) in the narrow center of the slit. The blue line is a synthetic spectrum of electron impact on H_2_O. The green line is the same for CO_2_. Both are adjusted to compensate for uncertainties in the energy distribution of both the cross section and electron flux. The positions of the ($2,0$) and ($1,0$) CO Cameron bands are also indicated. Note the scale change at 1750 Å by a factor of 2.5. From Feldman et al. (2018), ©AAS. Reproduced with permission

As described in Sect. 2.5.1 the most obvious spectral indicators of dissociative electron impact emissions are relative strengths of atomic emission features, more commonly referred to as line ratios. Dissociative electron impact on different neutral molecules produces unique atomic line ratios that can easily be distinguished from resonance scattering. Far-ultraviolet spectra shown in Figs. 24 and 25 yield diagnostic information about the composition and local plasma environment along the instrument’s line-of-sight. In the first example, from 2014 November 29 at high northern latitude, the spectrum is due to energetic electron ($>18~\text{eV}$) dissociative excitation of H_2_O. The second example, from 2015 January 30 at high southern latitude, is characteristic of electron excitation of CO_2_. Both spectra are taken from a distance of $\sim30~\text{km}$ from the comet, observing towards the shadowed nucleus to avoid contamination from emissions in the extended coma, as seen in the associated NAVCAM images. Details are given in Feldman et al. (2018).

Observations with the OSIRIS wide angle camera (WAC) filters of 67P pre-perihelion provided additional spatial distribution information about the prevalence of dissociative electron impact.

The WAC included multiple narrowband filters, including filters that were centered on OH (A-X) and O i 6300 Å, which are mainly produced from photodissociation of H_2_O. Dissociation of CO_2_, CO, and O_2_ is also capable of producing oxygen in the metastable $^{1}\text{D}$ state, which can radiate to the ground state via emission of a 6300 Å photon unless collisionally quenched at high inner coma densities (Festou and Feldman 1981; Cessateur et al. 2016).

From observations in early 2015, the column densities of H_2_O and CO_2_ derived from the narrowband filter images appeared to be consistently larger than those reported by MIRO, VIRTIS, and ROSINA/COPS when assuming photofluorescence of fragment species as the underlying process (Bodewits et al. 2016). The reason for this discrepancy was twofold; first, the emission was driven by electron impact rather than photofluorescence; second, multiple fragment species were emitting within the bandpass of some of the narrowband filters. The apparent discrepancy decreased between March and June 2015, opposite the trend of increasing production rates measured by other instruments, indicating that the presumed emission mechanisms, fluorescence and photodissociation, were now dominant over dissociative electron impact which was presumed to be a major emission mechanism at larger heliocentric distances. Evidence of a “plume” was also seen in these two filters, an indication that dissociative electron impact emissions may have been stronger within the a region on the sunward side of the nucleus for this period.

This shift in mechanism dominances in early 2015 suggests a change in the local electron population, resulting from electron cooling (Bodewits et al. 2016) and/or a decrease of solar wind electrons (Galand et al. 2020). Prior to the 2015 decrease, the CN emission likely contains contributions from dissociative electron impact of HCN and contamination from local $\text{CO}_{2}^{+}$ emission in the bandpass. After the 2015 decrease, fluorescence of CN following photodissociation of HCN is expected to dominate the CN filter images, but the exact contribution of the competing processes is unknown without further supporting laboratory studies of HCN dissociative electron impact.

Based on the early Alice detection of dissociative electron impact measurements by Feldman et al. (2015) the OSIRIS data is intriguing, but the plethora of possible emission mechanisms, such as ion-electron recombination (Sect. 2.5.2), capable of producing excess emission for e.g. O i 6300 Å leave other possibilities for the surplus emission. As highlighted in Bodewits et al. (2016), emission cross sections necessary for uncovering the role of dissociative electron collisions in the coma are lacking. While recent experiments for e.g. $e^{-}+\text{H}_{2}\text{O}$ (Bodewits et al. 2019) have made good progress on this front, there remain many systems without supporting laboratory measurements (see Sect. 3.1.3 and discussions therein).

## Methods

### Observations

#### Spacecraft-Based Observations

The Rosetta mission was the third cornerstone mission in the ESA Horizon 2000 programme. Originally targeting comet 46P/Wirtanen, a launcher issue and subsequent launch delay meant that in 2003 comet 67P/Churyumov-Gerasimenkov was selected as the mission target. Following launch in March 2004, Rosetta performed three Earth flybys and one Mars flyby to obtain a trajectory suitable for comet rendezvous. During this period, the mission also visited asteroid 2867 Steins in 2008 and 21 Lutetia in 2010. In August 2014 the spacecraft began the comet phase of the mission. The Rosetta orbiter instruments were a mix of remote sensing and in-situ experiments designed to characterise the cometary atmosphere and nucleus, while the Philae lander was equipped to carry out science at the surface itself. Most relevant to the investigation of space plasma phenomena was the Rosetta Plasma Consortium (RPC). RPC consisted of 5 instruments: the Ion composition Analyser (ICA, Nilsson et al. 2007), Ion and Electron Senor (IES, Burch et al. 2007), the Langmuir Probe (LAP, Eriksson et al. 2007), the Fluxgate Magnetometer (MAG, Glassmeier et al. 2007), and the Mutual Impedance Probe (MIP, Trotignon et al. 2007). RPC shared a common instrument control, interface and power unit, the Plasma Interface Unit (PIU, Carr et al. 2007). In addition, the Philae lander carried the ROsetta MAgnetometer and Plasma monitor instrument (ROMAP, Auster et al. 2007). The Rosetta Orbiter Spectrometer for Ion and Neutral Analysis (ROSINA, Balsiger et al. 2007) also contributed significantly to the analysis of the plasma environment around the comet, as well as Far Ultra Violet (FUV) observations by the Alice instrument (Stern et al. 2007). All data are available at ESA’s Planetary Science Archive (PSA, Rosetta section), and also the NASA PDS small bodies node, including valuable accompanying documentation, such as the RPC user guide,^5^ which describes instrument capabilities and caveats in detail. Any potential user of the RPC data is strongly encouraged to study these user guides to avoid data analysis issues. The original prime scientific goals of the Rosetta mission (and not just the plasma instruments) related to the comet phase are described in detail in the ESA Rosetta science definition team study report or so called ‘red book’ (ESA 1994).

**Fig. 26:**
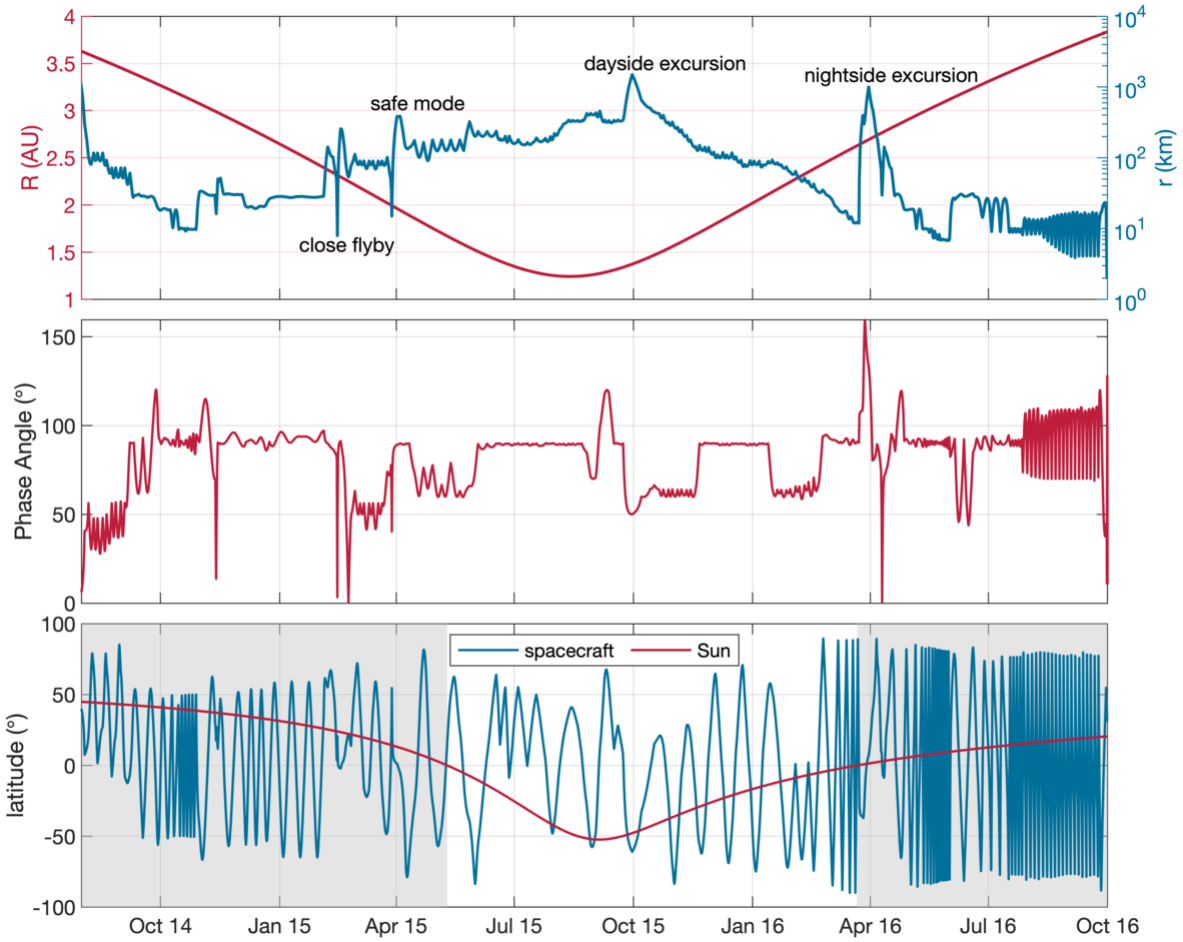
Top: Cometocentric distance $r$ (red) of Rosetta and heliocentric distance $R$ (blue) of the comet during the main phase of the Rosetta mission. Major milestones are marked by arrows. Middle: Phase angle between the spacecraft and the Sun in a cometocentric system. Bottom: Longitude of the Sun and spacecraft in a cometocentric system. The gray shaded boxes show northern summer, while the white background denotes southern summer. The latitude was not included as it changes periodically with the comet’s rotation rate ($\sim12~\text{hours}$) and would not be visible at this scale

The comet phase was broken into three distinct periods – pre-landing (up to and including delivery of Philae to the comet surface and its subsequent first science sequence in November 2014) and then the escort phase from mid-November 2014 to December 2015, followed by the extension period from January–September 2016. During the pre-landing phase, trajectory, operations and observations were very much driven by operational necessity to support the lander delivery, focusing on characterising the cometary environment to facilitate this. Initially during the escort phase, the trajectory design was based on science observation requirements and the predicted capability of successfully navigating that trajectory driven by the spacecraft drag in a particular level of cometary activity. These were initially based on ground-based estimates of cometary gas production, but then incorporated the Rosetta measurements themselves. Gas activity was deemed low enough up to about January 2015 to allow regular circular orbits around the nucleus, after which box like segments were implemented, including near and far flybys of the nucleus (see Fig. 26). This approach ensured that, in the case navigation was problematic, the spacecraft would be in an escape trajectory. This trajectory scheme had a long lead time and lacked flexibility to change closer to implementation. An alternative scheme was proposed, based on flying in the terminator plane, considered the most robust location by flight dynamics, and limiting instrument pointing requests (to facilitate a robust observation plan) and it was agreed to move towards a hybrid of the long term trajectory, with more flexible pointing, and the ‘terminator scheme’. During a close flyby of the comet through zero phase angle in February 2015, the Star Trackers (STR) onboard the spacecraft encountered issues reacquiring stars after losing track and locking on false stars, related to the dust environment near the comet nucleus. Although mitigation procedures were put in place, in March 2015, a subsequent close flyby at almost double the altitude, resulted in more prolonged STR tracking and related issues, which ultimately led to a safe mode onboard which moved the spacecraft away from the comet. As the source of the problem was dust and not gas pressure, following recovery of the spacecraft back to full operations, the spacecraft was brought back to the comet gradually to re-characterise its near-comet capabilities in a similar manner to its initial approach in 2014. The trajectories implemented during this period were so called pyramid orbits (hyperbolic trajectories on the day side that could form triangles) and also terminator orbits, which were considered the most operationally robust.

A robust trajectory design was implemented based on the simple requirement from the science team of getting as close as possible (ACAP) to the comet. The ACAP criteria meant that a majority of the time the spacecraft remained in the terminator plane, as at lower phase angles, STR behaviour led to higher altitudes being required. As the instrument and operations teams became more familiar with the new process, further requirements were added to optimise the trajectory for various goals, including aiming for various latitudes, which includes region optimising potential lander re-contact. Indeed, when the lander did communicate with the orbiter during June–July 2015, the flexibility of the new scheme allowed very rapid trajectory re-design to optimise potential contacts, which was absolutely necessary to facilitate this activity. Flying as close as possible was a huge challenge during the approach to perihelion, as activity and hence the dust environment grew, causing several retreats from the comet.

**Fig. 27:**
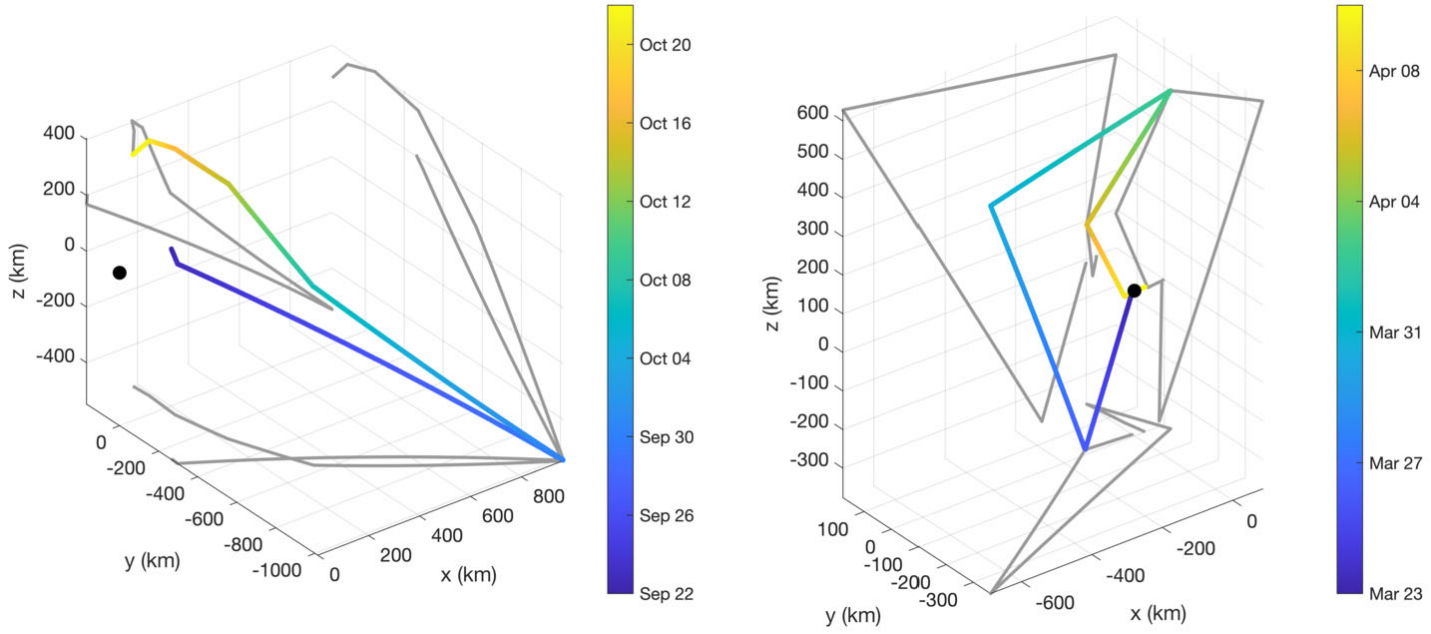
Left: Dayside excursion, Right: Nightside excursion in a CSEQ system. Color code is blue to yellow increasing in time

Following perihelion, with the exception of a $\sim 1500~\text{km}$ dayside excursion (Fig. 27, left) at the end of September, the rest of 2015 saw the spacecraft stepping closer to the comet and preparation for the extension period. The trajectory plan for 2016 included targeting a night side excursion $\sim 1000~\text{km}$ (Fig. 27, right), another zero phase flyby and a number of specific orbits, including a $24 \times 10~\text{km}$ elliptic orbit, so called ‘quiet nadir period’ avoiding spacecraft slewing and then finally the approach of Rosetta to the surface of 67P, during which time overflights going down to just under 3 km altitude were enacted, culminating on the soft impact of Rosetta ($<1~\text{m/s}$) on the surface of the comet in the Abydos site. It was during this final period that Philae’s location was finally confirmed via a dedicated search campaign.

#### Remote Sensing

Active comets usually display two primary types of tail: a dust tail, comprising grains ultimately released from the nucleus, and an ion, or plasma tail. The two tails are usually oriented at different angles: the curved dust tail arises from individual dust grains being affected to varying degrees by the effect of solar radiation pressure, whilst the ion tail traces pickup ions as they are carried downtail by the solar wind. Dust tails are visible as the constituent grains reflect sunlight, whilst ion tails are observable remotely as their ions emit light due to resonance fluorescent processes.

Despite the clearly unambiguous presence of pickup ions in the comet’s vicinity, as discussed extensively in this work, and the coordinated international observing campaign for Comet 67P utilising both ground- and space-based observatories (Snodgrass et al. 2016, 2017), there exist no unambiguous images of comet 67P’s ion tail during its 2014–2016 apparition. Unfortunately, it was therefore not possible to compare changes to the comet’s tail with variations in the solar wind in 67P’s vicinity, and other changes in the cometary environment measured in-situ by Rosetta instruments.

**Fig. 28:**
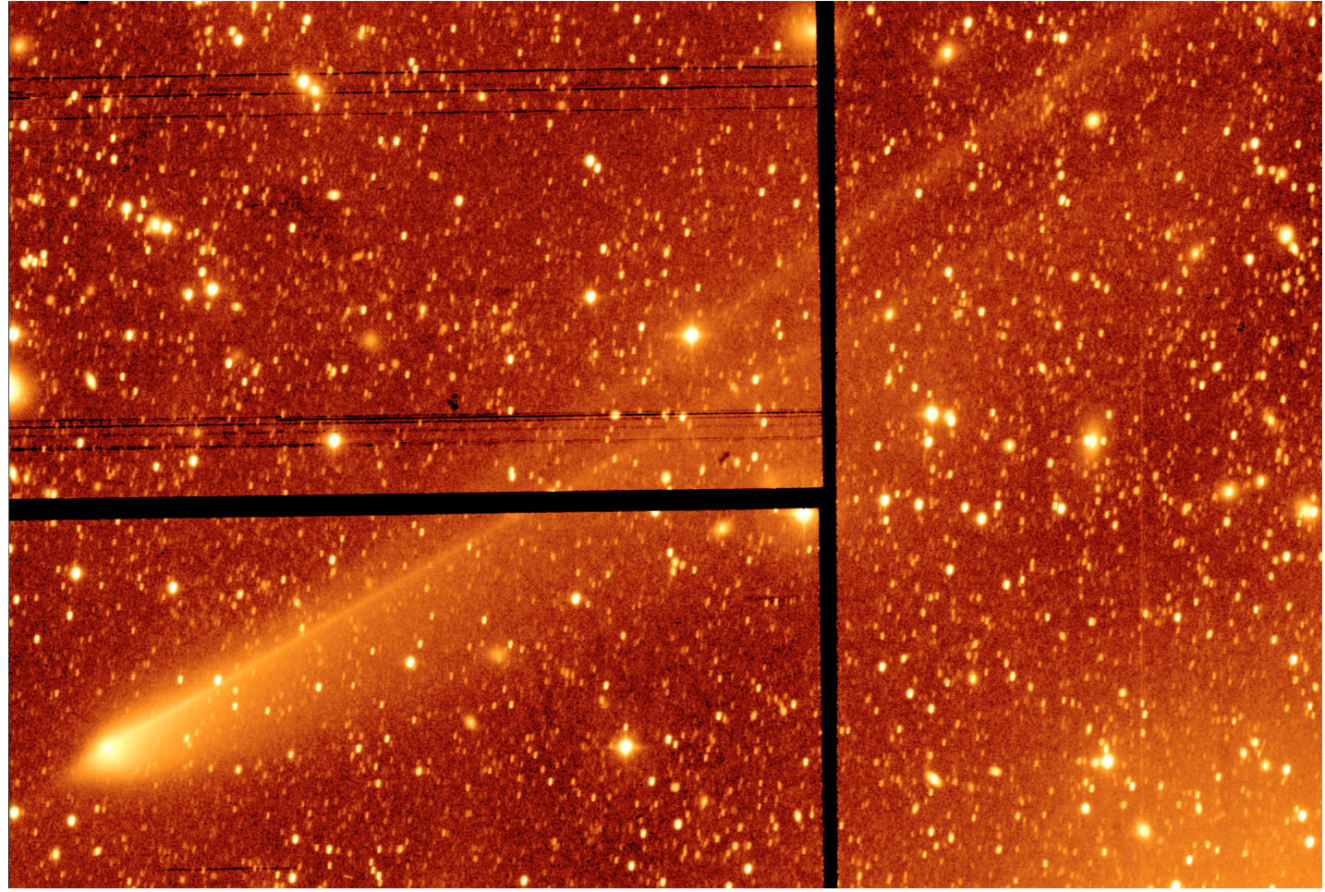
Image of 67P/C-G obtained with the 2.5 m Isaac Newton Telescope on La Palma on the morning of 19 January 2016. The picture was taken through a red filter; the apparent colour has been added to help pick out faint structures by eye. The tail extends 0.5 degrees from the nucleus (the apparent size of the full moon) before reaching the edge of the image, corresponding to a minimum length of 2.2 million km. Note that the thick black lines are gaps between CCDs in the array (the camera has 4 CCDs to cover half a degree). Credit: Alan Fitzsimmons/Isaac Newton Telescope

There are several reasons for the lack of detection of the ion tail, the primary one being the very low orbital inclination of 67P to the ecliptic. This low inclination meant that all observations from Earth were usually viewing both the comet’s dust and plasma tails, as well as the dust trail distributed along its orbit, all superimposed on one another (e.g. Fig. 28). There is no doubt that light emitted by ions in the comet’s vicinity was detected, but as the comet’s gas production rate was modest, the ion tail was likely overwhelmed by scattered and reflected sunlight originating at the dust tail and trails.

#### Laboratory Measurements and Cometary Analogues

The interpretation of in-situ and remote sensing observations of cometary plasma environments can be very challenging. In addition to the processes known from collisionless plasma physics collisional processes and chemical reactions become relevant to varying degrees. Accurate reaction efficiencies and their uncertainties are a crucial input of numerical models that describe the physics of the cometary plasma environment (Sect. 3.3). A complementary and informative method of investigation is by *laboratory measurements* of the physico-chemical processes and reactions at work. These laboratory studies aim at:

1. Characterising reaction efficiencies and energy transfers: this is done by measuring elastic (i.e. scattering) and inelastic (e.g. ionisation, excitation, dissociation) energy-dependent ion-neutral cross sections $\sigma (E)$ and reaction rate constants $k$ for two (or more) species colliding with each other,
2. Identifying molecular/atomic transitions for diagnosing local plasma conditions, including, for spectra analysis, threshold energies/electron energy dependencies,
3. Reproducing plasma physical conditions similar to those encountered at a comet (i.e. a comet analogue), but in a more controlled environment.

Typical target species at a comet include major and minor volatile constituents (e.g., H_2_O, CO_2_, CO, and O_2_, see Sect. 2.1.1), whereas impactors range from solar photons and plasma of solar wind origin ($\text{e}^{-}$, $\text{H}^{+}$, $\text{He}^{2+}$), including fast neutrals (energetic neutral atoms or ENAs also of solar wind origin, e.g., H and He atoms) produced from charge-exchange reactions (Sect. 2.2.1), to slow-moving cometary ions (partaking in the cometary chemistry per se). Secondary electrons produced by the first ionisation (photoelectrons, protoelectrons, etc.) can have sufficient energy to further ionise and excite the neutral medium (see Sect. 2.5 and Bhardwaj 2003).

Several techniques of investigation in the laboratory exist depending on the process studied; a description of the laboratory instrumentation is outside the scope of this paper, but the interested reader is encouraged to consult for example McDaniel et al. (1970), Bransden and McDowell (1992), Brunger and Buckman (2002), Itikawa (2007) and, specific to charge-transfer reactions and ENA production, the review of Lindsay and Stebbings (2005). In brief, any experiment starts with the controlled production of ions (for instance, with an electron cyclotron resonance ion source) or electrons, which are then guided by means of electromagnetic fields through a neutral gas jet, whose purity is kept at an optimum level. It ends with the detection of collision fragments and/or of light via several, usually complementary, techniques such as collision-induced fluorescence, translational energy spectrometry, or time-of-flight spectrometry, to name a few.

Laboratory measurements of ion-neutral collisions have been performed since the 1950s (e.g., Barnett et al. 1964) and have historically focused on reactions taking place in fusion plasmas and, more recently, in radiation dosimetry applications to investigate the transport of very fast charged particles through biological matter (Nikjoo et al. 2006). Both of these applications have led to a measurement bias towards higher impactor energies, typically above 1 keV/u (keV per *unified atomic mass unit*). This is in contrast to the practical needs of the space physics community, as solar wind energies are less than 1 keV/u on average (a proton beam at $400~\text{km}\,\text{s}^{-1}$ has a kinetic energy of about $830~\text{eV}/\text{u}$). Theoretical quantum mechanics developments in ion-neutral and electron-neutral collisions belong to a rich and dynamic field of investigation, which is most difficult at the low energies relevant to space physics. It is important to keep in mind that laboratory measurements and theoretical calculations often work in synergy and complement each other, for example when electron/ion energy ranges cannot be probed in the laboratory for technical reasons (see as an illustration the work of Anzai et al. 2012, on electron collisions with various molecules).

Table 4 gives a non-exhaustive list of physico-chemical processes for several targets of cometary relevance, as a resource for those looking for fundamental atomic data related to cometary atmospheres, as well as experimentalists searching for astrophysically-relevant pursuits. It must be mentioned that though extensive work on this area has been done, one must consider the uncertainties and scope of the laboratory data. For example, the PHIDRATES database (Huebner and Mukherjee 2015) provides reactions rates for two solar activities, and may not be applicable to every environment. The reaction rate survey of Anicich (1993) covers many bi- and tri-molecular collisions, but at a single temperature (300 K). In Table 4, the information related to dissociation and ionisation is bundled under the broader context of electron impact, for simplicity. In general, the data related to dissociative ionisation/emission by electron impact of cometary neutrals is sparse. Whereas the data for individual reactions may be applicable, it may be insufficient when considering large ranges of electron impact energies simultaneously. For example, many emission cross section studies suffer from one or more of the following: poor resolution (spectral or in terms of $E_{\text{electron}}$), relative (and uncalibrated) cross sections and energy dependencies, and/or large systematic uncertainties. Recent work has progressed on this front (e.g., Bodewits et al. 2019, for $e^{-}+\text{H}_{2}\text{O}$), but many more neutrals remain unstudied in sufficient detail (see also Sect. 2.5 on cometary emissions). Any and all efforts related to dissociation dynamics/emission, including the role of metastable states, should be encouraged.

**Table 4:** Recommended measured reaction rates and cross sections for cometary chemistry (CC), photoionisation (PI), photodissociation (PD), electron impact ionisation (EI), solar wind charge exchange (SWCX) and solar wind ionisation (SWI) of a few selected cometary neutrals. The column entitled “Nature” emphasises whether energy-dependent cross sections $\sigma (E)$ (and angular-dependent cross sections $\sigma (E,\theta )$) or reaction frequencies/rates $k$ (subscripts $in$ for ion-neutral, $en$ for electron-neutral and $ei$ for electron-ion recombination reactions) are given. In some cases, only the most recent reviews are provided. Databases are in capital letters. Emphasis has been placed on EI of H_2_O, CO_2_, CO and O_2_, which are listed out here instead of the more recent synoptic review of Anzai et al. (2012), because they usefully contain tabulated values for the cross sections. DR stands for “Dissociative electron Recombination”, for which a synoptic table of reaction rate constants can usefully be found in Appendix B of Heritier et al. (2017a). Prominent emissions are also given with wavelengths $\lambda $ in nm

Process	Target(s)	References	Nature	Comment	Prominent emissions
CC	H_2_O, CO_2_, CO, etc.	McElroy et al. (2013), Anicich (1993), Wakelam et al. (2012) (KIDA), McElroy et al. (2013) (UMIST), Pierce and A’Hearn (2010), Heritier et al. (2017a)	$k_{in}$, $k_{ei}$	Hundreds to thousands of reactions, at various temperatures, including DR	
PI, PD	H_2_O, CO_2_, CO, etc.	Huebner and Mukherjee (2015)	$k_{\text{ph}}$	140 target species, vs solar activity, dissociation/excitation	O i *λ*557.7, *λ*630.0, CO($a\,^{3}\Pi $) *λ*180-300
	H_2_O, CO_2_, CO, etc.	Heays et al. (2017)	$\sigma _{\text{ph}}(\lambda )$	102 target species	
EI	H_2_O	Itikawa and Mason (2005), Bodewits and Hoekstra (2019)	*σ*(*E*)	Dissociation/excitation included	H_2_O^+^ $425\text{--}675~\text{nm}$, OH $300\text{--}325~\text{nm}$, H i $400\text{--}500~\text{nm}$
	CO_2_	Itikawa (2002)	*σ*(*E*)	Dissociation/excitation included	$\text{CO}_{2}^{+}$ $293\text{--}440~\text{nm}$, $218\text{--}227~\text{nm}$
	CO	Itikawa (2015)	*σ*(*E*)	Dissociation/excitation included	CO $127\text{--}200~\text{nm}$
	O_2_	Itikawa (2009)	*σ*(*E*)	Dissociation/excitation included	$\text{O}_{2}^{+}$ (A-X) 180–530 nm, (B-A) 450–850 nm, O i 130 nm, 610 nm
	N_2_	Itikawa (2006)	*σ*(*E*)	Dissociation/excitation included	$\text{N}_{2}^{+}$ (B-X) 390–471 nm
	CH_4_	Song et al. (2015)	*σ*(*E*)	Dissociation/excitation included	
	Oxygen-bearing molecules	McConkey et al. (2008)	*σ*(*E*)	Dissociation/excitation included	
	H_2_O, CO_2_, CO, O, N_2_, H	Cravens et al. (1987)	*σ*(*E*), $k_{en}(T_{e})$	Dissociation/excitation included	
	CO, O_2_, N_2_, H_2_, H	Brunger and Buckman (2002)	*σ*(*E*,*θ*)	Differential cross sections (elastic/inelastic)	
SWCX	H_2_O	Simon Wedlund et al. (2019)	*σ*(*E*)	hydrogen/helium impactors	He ii 47–63 nm
	H_2_O, CO_2_, CO, etc.	Mullen et al. (2017) (KRONOS)	*σ*(*E*)	State-selective CX cross sections for highly charged impactors	C iv 17–27 nm, O vii 1–116 nm, Fe xvi 1–90 nm
SWI	H_2_O	Simon Wedlund et al. (2019)	*σ*(*E*)	hydrogen/helium impactors	

Other recent databases listing cross sections (sometimes even differential ones) and reaction rates for various applications (not necessarily astrophysical) are for example LXCat (Pitchford et al. 2017) and QDB (Tennyson et al. 2017), both dedicated to low-temperature plasmas; they function as an aggregator to many other more specialised databases. A most recent addition to the field of collisional data is the updated NIFS database (Murakami et al. 2020), which compiled and evaluated hundreds of thousands of datasets involving many of the collision processes in Table 4. Many useful references for electron-neutral and ion-neutral reactions are also contained in the appendices of the excellent book of Itikawa (2007), and in the critical review by Avakyan (1998) for photo-, electron-impact, and proton/hydrogen-impact ionisations, and for UV/visible emissions in an aeronomic context.

Gaps in the current laboratory measurements must be addressed to better interpret observations and constrain numerical models (see, e.g., Huestis et al. 2008; Simon Wedlund et al. 2019; Gronoff et al. 2020). Several directions of investigation benefiting cometary studies can be envisioned:

1. Extend integral (and whenever possible, differential) cross section measurements towards the lower energy range ($0.01< E\leq 1~\text{keV/u}$) with precise evaluation of uncertainties, especially for fast ion collisions (charge-transfer reactions, momentum transfer and ionisation), leading to recommended datasets for e.g. electron capture, stripping, and ionisation of important molecules (CO, CO_2_, …),
2. Expand the current range of neutral targets (including H, H_2_, He, C, N, O, CH_4_, OH, H_2_O, Ne, N_2_, CO, O_2_ and other minor cometary volatiles) and impactors (photons, electrons, H, $\text{H}^{+}$, He, $\text{He}^{+}$, $\text{He}^{2+}$, and high ion states of C, O and Fe), and characterise them in ionisation and emission at relevant energies,
3. For dissociative electron impact emissions, systematically measure cross sections for the main cometary neutrals (H_2_O, CO_2_, CO, O_2_) at high energy resolution below 1 keV impact energy (see Sect. 2.5),
4. Characterise ion-ion reactions energetics and energy-dependent cross sections (e.g., $\text{H}_{2}\text{O}^{+}_{\text{fast}}+\text{H}_{2}\text{O}^{+}$). Rosetta measurements show that pickup cometary ions accelerated by the solar wind convective electric field play a large role in the overall dynamics of the cometary plasma environment.

All of these points have strong implications on our current knowledge of plasma-neutral reactions in the cometary coma and will help constrain global and local numerical models more precisely.

**Cometary plasma analogues**. Is it possible to create a laboratory setup which can serve as an analogue to the plasma at a comet? To build up a laboratory experiment that mimics all processes at work at a comet at the same time is not realistic, because the applicable scaling laws are different for different processes. However, it may be possible to conduct experiments on specific processes in the comet plasma environment and by studying those scaled-down systems learn something about comets. In recent years, advances in laser-plasma interaction experiments have provided a means to this end. Potentially suitable experiments have been performed on diamagnetic cavities and bow shocks (see Sects. 4.6 and 4.2). In both cases these experiments have been performed in a large plasma device (Gekelman et al. 1991), where discharges create a background plasma with densities of $10^{12}$–$10^{13}~\text{cm}^{-3}$, electron temperatures $\sim10~\text{eV}$, in a magnetic field of 0.03–0.08 T. A laser pulse is shone on a target, which sends a carbon plasma, $n_{\mathrm{e}}\approx 10^{15}$–$10^{17}~\text{cm}^{-3}$ initially, expanding into the surrounding magnetised background plasma. Bonde et al. (2015, 2018) observed the formation of a diamagnetic cavity as the laser produced plasma expanded into the background. They presented measurements of the electric field both in the cavity, at the boundary, and outside of it. The size of the diamagnetic cavity was a few centimetres. The net charge of the cavity was found to be positive, which is also true for comets where there is an ambipolar field near the nucleus (see Sects. 4.1 and 4.6). The charge density spatial distributions differ somewhat between the laboratory and the comet, and the pulsed laser produced plasma expands at $130~\text{km}\,\text{s}^{-1}$, whereas the continuously generated comet plasma expands at only a few $\text{km}\,\text{s}^{-1}$. In spite of such differences, the experiment may still be used to understand the physics of the diamagnetic cavity boundary.

Niemann et al. (2014) conducted experiments, where the laser produced plasma expanded at $500~\text{km}\,\text{s}^{-1}$. This led to the formation of a subcritical shock (Alfvénic Mach number $M_{\mathrm{A}}\approx 2$ which is below the critical number of $\sim 2.7$ Balogh and Treumann 2013). The authors also presented hybrid simulations of the experiments, showing an asymmetry to some extent resembling that of the infant bow shock (Sect. 4.2). Heuer et al. (2018) and Heuer et al. (2020) performed experiments on the ion/ion-beam instabilities behind shock formation, particularly of quasi-parallel shocks. At another laser facility (Boehly et al. 1995), shock experiments were carried out which allowed the electron and ion distribution functions to be measured (Schaeffer et al. 2019), and these experiments were modelled, using PIC simulations (Schaeffer et al. 2020). The high expansion velocities in all these shock experiments are similar to the relative velocity between a comet and the solar wind, and the overall features resemble what is seen in comet–solar wind interaction very well. Both in the case of shock experiments and for the diamagnetic cavity experiments described above, it is likely that comparing PIC simulations of the cometary and laboratory plasmas will establish what the similarities and differences are on a detailed micro-physical level. These shock experiments also produce diamagnetic cavities, but considering the much higher expansion velocities used in the shock experiments, the dedicated diamagnetic cavity setups are likely better comet analogues.

### Multi-instrument Analysis Using a Physics-Based Model

#### Multi-instrument Analysis of the Plasma Density

While it is very valuable to analyse the dataset from one single instrument, the science return is enhanced by combining coincident observations from different instruments through a physics-based model. Here we apply such an approach to Rosetta neutral gas, particle, and plasma multi-instrument measurements, which are linked through the continuity equation applied to the plasma in the close environment of comet 67P. The goal is to assess the cometary plasma balance (see Sect. 2.2.4), to confirm the major plasma sources, and to establish the major plasma losses, at large heliocentric distances (2–4 AU) at comet 67P.

**Fig. 29:**
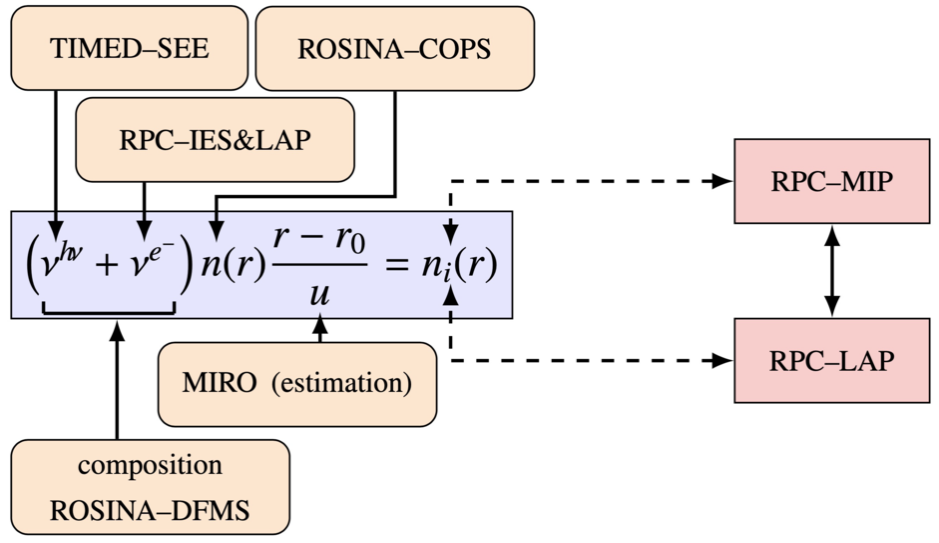
Multi-instrument approach applied to the analysis of the plasma density. Coincident neutral gas, particle, and plasma observations are linked though the solution of the continuity equation in order to assess the plasma balance. The ion bulk velocity is derived from observations from MIRO. The solar flux observed by TIMED-SEE at Earth is extrapolated in phase and distance to comet 67P. From Heritier et al. (2018), reproduced with permission ©ESO

For low outgassing rates (local $Q=10^{25}\text{--}10^{27}~\text{s}^{-1}$), photo-ionisation and electron-impact ionisation have been found to be major contributors to the cometary plasma (Heritier et al. 2018, see Sect. 2.2.1). The chemical loss timescales (associated with electron-ion dissociative recombination) are negligible compared with transport timescales (see Sect. 2.2.2) and steady-state conditions hold. As a result, the continuity equation applied to the cometary plasma is reduced to a balance between the sum of the ionisation production rates and the divergence of the plasma flux (see Sect. 2.2.4). The solution to this equation is given by Eq. (9). A multi-instrument approach is undertaken by comparing the plasma density observed by RPC-LAP (Eriksson et al. 2007) and RPC-MIP (Trotignon et al. 2007) and the modelled plasma density along the trajectory of $\textit{Rosetta}$. The modelled plasma number density is derived from coincident observations of energetic electron spectral fluxes by RPC-IES (Burch et al. 2007), neutral number density by ROSINA-COPS, and neutral composition by ROSINA/DFMS (Balsiger et al. 2007; Galand et al. 2016; Heritier et al. 2018). The approach is illustrated in Fig. 29. The cometocentric distance $r$ corresponds here to the one of Rosetta. A detailed description of the approach and a justification of the assumptions are presented in Galand et al. (2016).

At these low outgassing rates and close to the comet, ions are assumed to travel radially at the same bulk velocity ($u$ in Fig. 29) as the neutrals (corresponding to the neutral terminal expansion velocity). The values are derived from observations from MIRO (Gulkis et al. 2007) and typically range between 400 and 750 m/s (Gulkis et al. 2015; Biver et al. 2019) at large heliocentric distances (2–4 au).

The coma at the level of activity considered is optically thin, hence the photo-ionisation frequency, $\nu ^{h\nu}$, is independent of the cometocentric distance (Heritier et al. 2018; Heritier 2018). This frequency is derived from the daily value of the spectral solar flux obtained from measurements by the Thermosphere Ionosphere Mesosphere Energetics and Dynamics (TIMED) – Solar EUV Experiment (see Woods et al. 2005). As the Sun, Earth, and comet 67P are not usually aligned, a phase shift is applied in order to identify the most representative period as seen from Earth to infer the solar spectral flux at comet 67P. The longitudinal phase shifted spectral flux $J^{h\nu,1au}$ at 1 AU is adjusted to the heliocentric distance, $d_{h}$, of comet 67P. The photo-ionisation frequency, weighted with respect to the local neutral composition measured by ROSINA-DFMS, is given by:

17 $$ \nu ^{h\nu} = \dfrac{1}{d_{h}^{2}}\sum _{p} \int _{\lambda _{min}}^{ \lambda ^{th}_{p}} \upsilon _{p} \, J^{h\nu ,1au}(\lambda )\sigma ^{h \nu , ioni}_{p}(\lambda ) \,\mathrm{d}\lambda $$

where $\upsilon _{p}$ is the volume mixing ratio of the species $p$ derived from ROSINA-DFMS and $\sigma ^{h\nu , ioni}_{p}(\lambda )$ is the photo-ionisation cross section of the neutral species $p$ through absorption of solar photons of wavelength $\lambda $. $\lambda ^{th}_{p}$ is the wavelength threshold around 90–100 nm (see Sect. 2.2.1). The minimum wavelength, $\lambda_{min}$, considered is typically around 0.1–1 nm.

The coma is anticipated not to be dense enough to have the energetic, suprathermal electrons undergo significant energy loss (Heritier 2018). The electron-impact ionisation frequency, $\nu^{e-}$ is hence assumed to be constant within the radial column between *Rosetta* and the cometary nucleus. Weighted with respect to the local neutral composition measured by ROSINA-DFMS, it is given by:

18 $$ \nu ^{e-} = \sum _{p} \int _{E^{th}_{p}}^{E^{max}} \upsilon _{p} \, J^{e-}(E) \sigma ^{e-, ioni}_{p}(E)\, \mathrm{d}E $$

where $\sigma ^{e-, ioni}_{p}(E)$ is the electron-impact ionisation cross section of the neutral species $p$ through collisions by electrons of energy $E$. The energy threshold $E^{th}_{p}$ is around 12–14 eV (see Sect. 2.2.1) and $E^{max}$ is typically around 200 eV. The relevant ionisation cross-sections are largest at electron energies of around 100 eV. $J^{e-}$ is the suprathermal electron spectral flux measured by RPC-IES. It is derived from in-situ measurements by RPC/IES (Burch et al. 2007) (see Sect. 3.1). The measured flux is corrected for the spacecraft potential ($V_{S/C}$), measured by RPC/LAP (Eriksson et al. 2007), using Liouville’s theorem (Galand et al. 2016).

19 $$ \frac{J^{e-}(E)}{E} = \frac{J_{IES}}{E_{IES}} \quad \text{where} \quad E \, [\text{eV}]= E_{IES} \, [\text{eV}]- V_{S/C}\, [\text{V}] $$

**Fig. 30:**
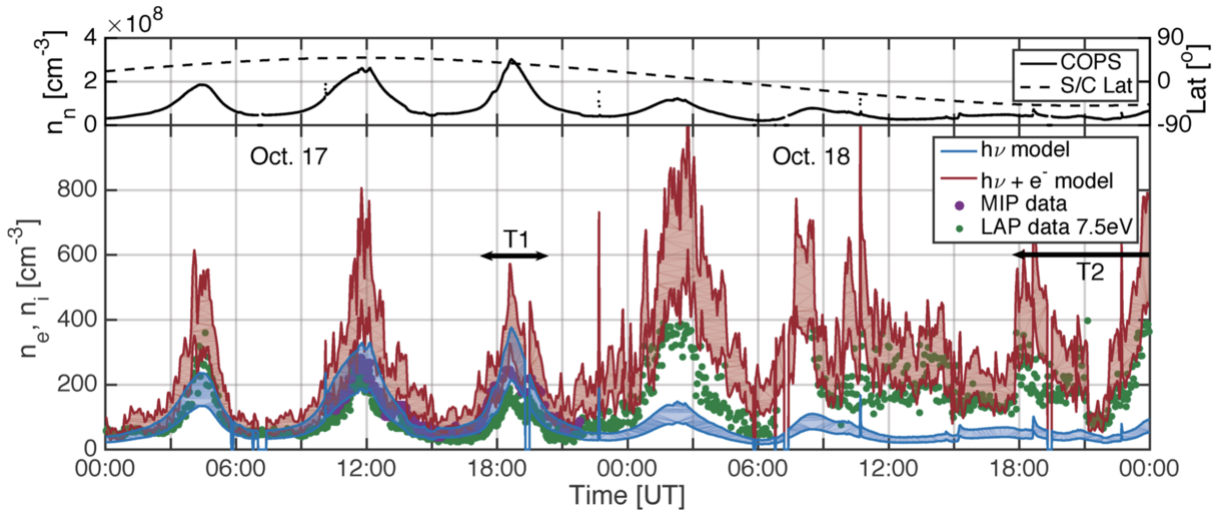
Top: ROSINA-COPS total neutral number density (solid line) measured at Rosetta and the sub-spacecraft latitude (dashed line) as a function of time. Bottom: Ionospheric density as a function of time. The period shown is 2014 October 17–18. The blue (red) curves correspond to the calculated plasma density assuming photoionization alone (photoionization and electron-impact ionization). The vertical spread of these curves corresponds to the range of ion outflow velocity considered, spreading from 400 m/s (top boundary) to 700 m/s (bottom boundary). The RPC-MIP electron density is shown with large, violet dots. The RPC-LAP electron density is shown with small green dots, assuming an electron temperature of 7.5 eV (light green). Due to a different operation mode, there is no RPC-LAP electron density available between 07:00 and 08:30 UT on 2014 October 18. The subsolar latitude was $40^{\circ}$ and ${\textit{R}osetta}$ was in the solar terminator plane. From Galand et al. (2016), Fig. 15
  In contrast, the plasma number density over the *southern, winter hemisphere* during pre-perihelion is dominantly produced through electron-impact ionisation (Galand et al. 2016; Heritier 2018). If only the photo-ionisation contribution is taken into account, the modelled plasma density largely underestimates the observed plasma density (see 2014 October 18 in Fig. 30). The observed electron flux is more intense over the winter hemisphere than over the summer hemisphere. This intensification over-compensates the weaker neutral density over the winter hemisphere; as a result, the plasma density was found to be higher over the winter hemisphere than over the summer hemisphere (see Fig. 30).
- **During post-perihelion, quiet solar conditions:** As the solar activity decreased over the course of the escort phase, the photo-ionisation frequency at a given heliocentric distance is lower pre-compared with post-perihelion; the relative importance of photo-ionisation compared with electron-impact ionisation decreases. The observed plasma density at post-perihelion is higher in the summer/autumn (southern) hemisphere compared with the winter/spring (northern) hemisphere. The strong electron fluxes over the winter/spring hemisphere did not compensate for the weaker neutral density (Heritier et al. 2018), as it did during pre-perihelion. The multi-instrument analysis was applied during the return from the cometary tail excursion in April 2016. The very good agreement between the observed and modelled plasma density demonstrates the robustness of the model over a large range of cometocentric distances up to 80 km (Heritier et al. 2018).
- **During solar events:** A Co-rotating Interaction Region (CIR, see also Sect. 2.4) hit comet 67P over several solar rotations in summer 2016 (Hajra et al. 2018a). The multi-instrument analysis was applied to the disturbed cometary environment in July and August 2016. The relative importance between photo-ionisation and electron-impact was found to be highly variable, with the latter sometimes largely dominating the former and increasing by one order of magnitude during quiet and disturbed times. The very good agreement between the observed and modelled plasma densities attests that the enhancement seen in the plasma density during the CIR was driven by an increase in the electron-impact ionisation frequency (see Fig. 31), and was not due to a plasma compression driven by the increase in the solar wind dynamic pressure (Heritier et al. 2018; Hajra et al. 2018a).

The multi-instrument analysis of the plasma density is robust over a large variety of conditions (e.g., season, hemisphere, pre- and post-perihelion, quiet and disturbed solar activity) encountered by *Rosetta* at large heliocentric distances:

- **During pre-perihelion, quiet solar conditions:**
  Over the *northern, summer hemisphere*, there is a very good agreement between the observed and modelled plasma densities (see 2014 October 17 in Fig. 30), even when the electron-impact ionisation is neglected (Vigren et al. 2016). The pre-perihelion phase started shortly after solar maximum (Heritier et al. 2018): photo-ionisation is the dominant source of ionisation, while electron-impact ionisation has a marginal contribution over the most outgassing hemisphere (Galand et al. 2016). A dependence in $r^{-1}$ of the plasma density was observed (Galand et al. 2016), as predicted by the model (see Fig. 29).

**Fig. 31:**
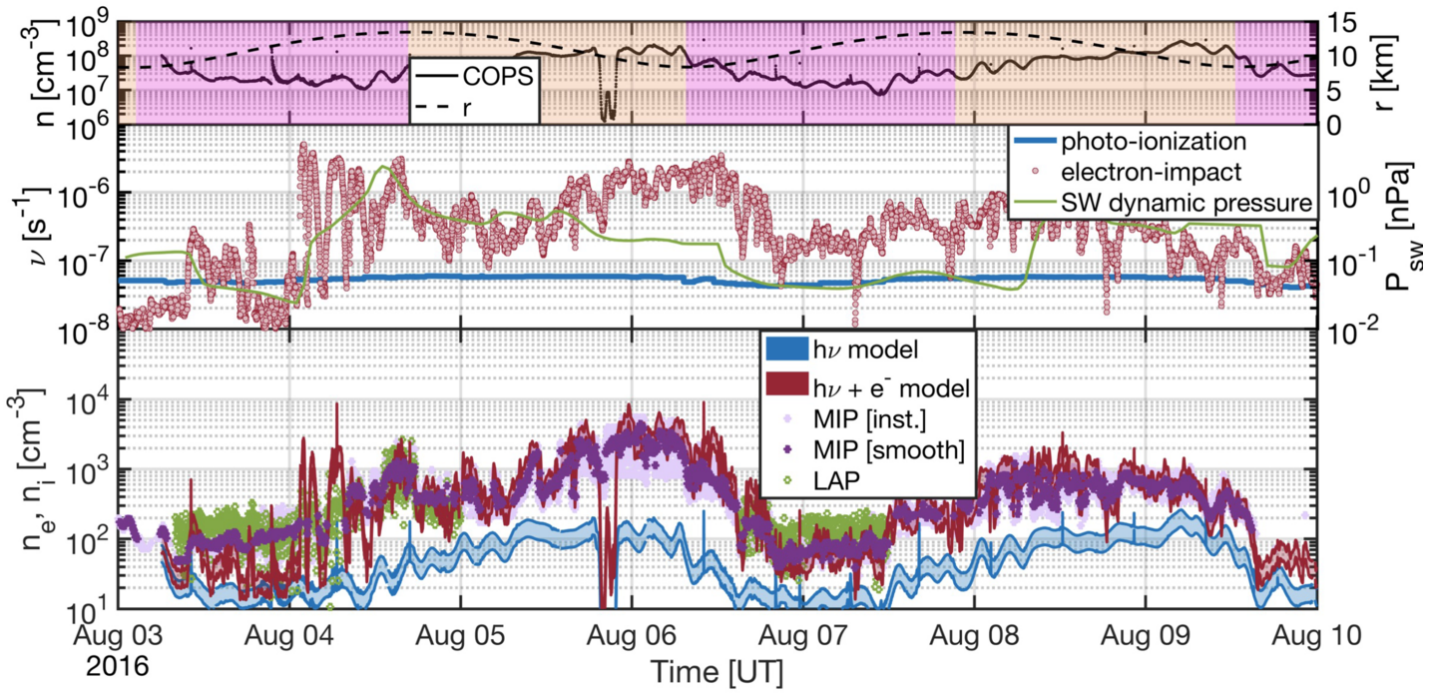
Top: Time series of the ROSINA-COPS measured neutral number density (full line) and cometocentric distance of the spacecraft (dashed line). Middle: Time series of the photo-ionisation frequency $\nu ^{h\nu}$ (blue curve) and total ionisation frequencies ($\nu ^{h\nu} + \nu ^{e-}$) (red dots). Seasonal variations are colour coded in the top panel with pink for spring (northern hemisphere) and yellow for autumn (southern hemisphere). Bottom: Time series of the RPC-MIP measured electron number density (pink dots), smoothed using a 5-minute average (purple) and RPC-LAP derived total ion densities (green). Simplified modelled ionospheric densities (Eq. (9)) using photo-ionisation only (blue) and both photo-ionisation and electron-impact ionisation (red), assuming outflow velocity from 400 m/s (upper bound) to 700 m/s (lower bound). The solar wind dynamic pressure as predicted by the Tao et al. (2005) model is plotted in green (middle panel) to illustrate the effect of the CIR impact. From Heritier et al. (2018), reproduced with permission ©ESO
- **At the end of the** ***Rosetta*** **mission:** *Rosetta* made a controlled landing on the nucleus’ surface which offered the opportunity for taking the first-ever plasma measurements all the way down to the surface (Heritier et al. 2018). Along the descent which covered a large range of latitudes and longitudes over the northern, spring hemisphere, the multi-instrument analysis showed that electron-impact ionisation was the dominant source of the cometary plasma. However, the margin of errors was large due to large uncertainties in the gas bulk velocity. A refined approach was developed for which the ion bulk velocity is no longer constant, but equal to the expansion velocity of the adiabatic gas and varies from 400 m/s close to the surface to 800 m/s (terminal flow velocity) at a cometocentric distance of about 20 km. Apart from a slight overestimation when $\textit{Rosetta}$ was over the nucleus’ neck, modelled plasma density from the refined approach agrees extremely well with the cross-calibrated RPC-MIP/RPC-LAP densities. The peak in the plasma density profile, predicted to be located at twice the apparent radius of the nucleus (see Sect. 2.2.4) is clearly apparent in the observed plasma density dataset.

In summary, the multi-instrument analysis of the plasma density developed for a local outgassing rate between $10^{25}$ and $10^{27}~\text{s}^{-1}$ (that is, less than 2–4 AU for comet 67P Heritier et al. 2018) and cometocentric distances close to the nucleus ($<100~\text{km}$), has shown that the cometary plasma is produced through photo-ionisation of solar EUV radiation and electron-impact ionisation; the relative difference in the sources varies with season, hemisphere, solar activity and solar events. The plasma is lost through transport. The ions are strongly coupled to the neutrals, as the region analysed is below or close to the ion exobase (Galand et al. 2016). The ions do not seem to suffer any significant acceleration, at least if there is any, it is limited and does not affect the cometary plasma density. Very close to the nucleus (a few km above the surface), the analysis is improved by taking into account the neutral expanded flow (Heritier et al. 2017b). The robust multi-instrument approach is a relevant tool not only for the analysis of the plasma source and loss, but also for the analysis of solar events, as shown with the example of the CIR events in summer 2016.

#### Multi-instrument Analysis of the FUV Emissions

To enhance the science return from the Alice FUV spectrometer, a multi-instrument analysis has been applied to the observed FUV emissions from atomic hydrogen, oxygen, and carbon (Galand et al. 2020; Stephenson et al. 2021). This analysis combines gas, particle, and light measurements, taken simultaneously, from in situ and remote-sensing instruments onboard Rosetta, utilising a simple, physics-based model. The multi-instrument analysis has been applied to establish the source and identify the nature of the FUV emissions, as well as to provide further constraints on the composition of the neutral coma. The model focusses on dissociative excitation of cometary molecules by electron impact (see Sect. 2.5) which has been identified as a source of FUV emissions at comet 67P (e.g. Feldman et al. 2015, 2016, 2017). The brightness of an FUV emission line from electron impact is given by Eq. (15). As the analysis is focussing only on low outgassing rates ($Q<2 \times 10^{26}~\text{s}^{-1}$), the differential flux, $J(E)$, of energetic electrons does not undergo any significant energy degradation in the coma and can be assumed to be constant throughout the column along the line of sight of the FUV spectrograph (Chaufray et al. 2017; Heritier 2018). As such, the simplified version Eq. (16) can be used to calculate the brightness of the modelled emissions.

**Fig. 32:**
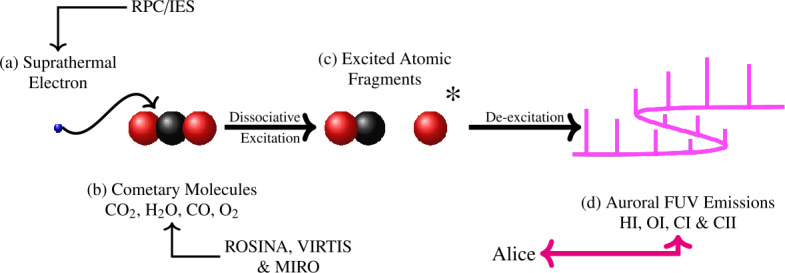
Schematic of the multi-instrument analysis applied to FUV emissions. From Stephenson et al. (2021), reproduced with permission ©ESO

A schematic outlining the multi-instrument analysis is shown in Fig. 32. The observed brightness from the Alice spectrograph is compared with the modelling brightness. The latter is derived from observations of the column density of the major neutral species along the line of sight and the differential flux of suprathermal electrons. By combining coincident in situ and remote-sensing measurements, uncertainty due to the variability of the neutral gas and electron flux is minimized.

This analysis has been applied to two different viewing geometries: nadir viewing and limb viewing. When viewing nadir, the composition and density of the neutral gas along the line of sight are well constrained as the major species do not have an extended source and the column of gas between the nucleus and the Rosetta spacecraft is coming from the same region on the surface. Cases have been selected when the surface of the nucleus was in shadow, reducing contamination from solar flux reflected off the nucleus. It is possible to extrapolate from local measurements of total number density by ROSINA/COPS and neutral composition by ROSINA/DFMS (Balsiger et al. 2007), assuming a $1/r^{2}$ profile as observed (Hässig et al. 2015; Bieler et al. 2015b). Measurements from VIRTIS (Coradini et al. 2007) can also be used for deriving the column density of H_2_O, CO, and CO_2_.

For limb viewing, the column density of H_2_O, CO, and CO_2_ can be derived from observations by the sub-mm spectrograph, MIRO (Gulkis et al. 2007) and the infrared spectrometer VIRTIS. However, the column density of O_2_ cannot be probed remotely with these instruments. It is also not possible to extrapolate it from in-situ composition measurements, as the neutral gas along the line of sight originates from many regions of the nucleus.

The emission frequency $\nu _{l}^{X}$, for a given neutral species $l$ and emission line $X$ is given by:

20 $$ \nu ^{X}_{l} = \int \limits _{E^{th}_{l}}^{E^{Max}}\sigma ^{X}_{l}(E) \, J^{e-}(E) dE $$

where $\sigma _{l}^{X}$ is the emission cross-section of the emission line $X$ due to dissociative excitation of the molecular species $l$ by an energetic electron of energy $E$. A review of the relevant emission cross sections used is given in (Stephenson et al. 2021). $J^{e-}(E)$ is the suprathermal electron spectral flux, introduced already in Eq. (18) and corrected to the spacecraft potential as given in Eq. (19) (see Sect. 3.2.1). $E^{th}_{l}$ is the energy threshold for the dissociative excitation process (15–25 eV). $E^{max}$ is the maximum energy considered, typically around 200 eV for the observed RPC-IES spectral fluxes.

As well as emissions from dissociative excitation, photodissociation of cometary molecules generates emissions in the FUV. These are modelled as discussed in Stephenson et al. (2021). Measurements of the solar photon flux by TIMED/SEE orbiting the Earth is extrapolated to the position of 67P assuming a $1/r_{h}^{2}$ drop off, where $r_{h}$ is the heliocentric distance. As 67P and Earth were at most times out of solar phase during the Rosetta escort mission (except in February 2015), the TIMED/SEE measurements are taken from the same Carrington longitude as 67P for each observation. The neutral coma has been assumed to be optically thin, which again is only valid at large heliocentric distances.

**Fig. 33:**
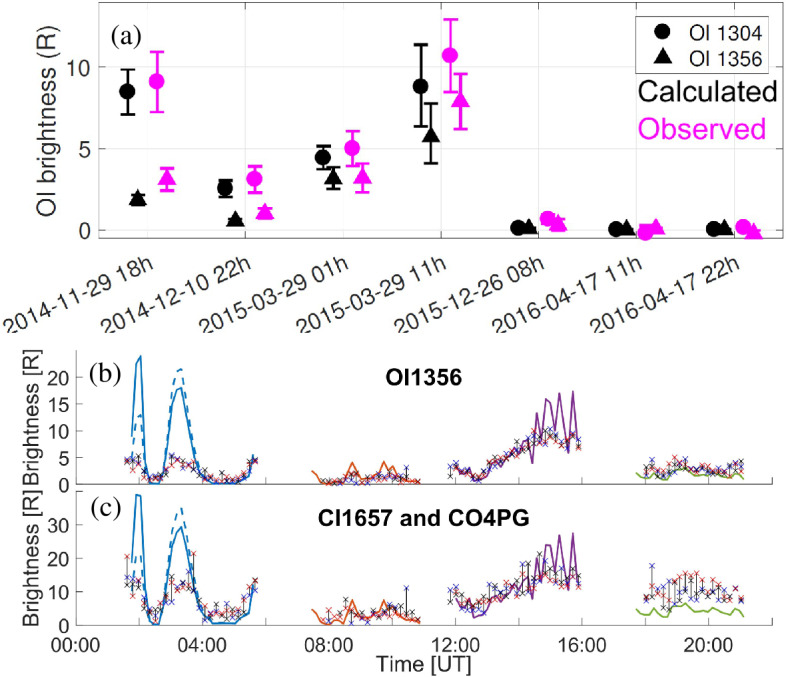
(**a**) Comparison of modelled (black) and observed (magenta) atomic oxygen emissions for seven cases in the northern hemisphere of 67P with a nadir viewing. Measured and modelled points for a given date and time are offset for visibility. From Galand et al. (2020). (**b**) and (**c**) Comparison of modelled (solid lines) and observed (crosses) FUV emissions during a corotating interaction region at comet 67P on 4th Aug 2016. Each time interval of consecutive measurements is plotted in its own color to better distinguish them. Both from Stephenson et al. (2021), reproduced with permission ©ESO. Further atomic emission lines to those shown have been modelled in both of the above studies

From the multi-instrument analysis of nadir-viewing emissions, Galand et al. (2020) and Stephenson et al. (2021) established that dissociative excitation is the major source of FUV atomic emissions at large heliocentric distances (see Fig. 33a). This is true both over the northern, summer hemisphere where H_2_O was the dominant outgassing species and over the southern, winter hemisphere where CO_2_ played a larger role compared to water. The relative contributions of the neutral molecules to the emission lines are very different between the northern and southern hemispheres, for large heliocentric distances, but in both cases dissociative excitation by electron impact was the key driver of the emissions.

The multi-instrument analysis applied to limb-viewing observations showed that the variation in the FUV emission brightnesses is correlated with the variation in the electrons measured in situ at Rosetta (Figs. 33b and 33c; Galand et al. 2020; Stephenson et al. 2021). This demonstrated that the energetic electrons responsible for the FUV emissions are not produced locally, but rather have undergone a large-scale acceleration (see Sects. 2.3.2, 4.1 and Galand et al. 2020). This analysis has revealed the auroral nature of the FUV atomic emissions seen by Rosetta and the relevance of using OI 1356 Å as a remote-sensing tracer of solar wind electron variability.

### Numerical Models

By accompanying 67P for two years, the Rosetta orbiter provided a wealth of fresh new information during various stages of the comet’s life, but as with all single-spacecraft missions, it could only be at one spot in time and space, and possibly the most intriguing plasma physics in the solar wind – comet interaction unfolded partially out of reach of Rosetta instruments. It makes the coupling of observational and modelling efforts a necessity. For instance, while Rosetta was close to the comet nucleus investigating the diamagnetic cavity (Sect. 4.6 and Goetz et al. 2016a,b; Henri et al. 2017; Hajra et al. 2018a), it was impossible to tell how the upstream solar wind variability influenced the cometary plasma environment (e.g. did a local or external physical mechanism cause the observed variability of the boundary of the diamagnetic cavity?).

Over the last decades the numerical methods used to advance theory and to analyse, interpret and predict measurements have been revolutionised. The increasing availability and expansion of computational resources has made possible the implementation of more precise and complete numerical models at larger spatial scales.

Numerical models in plasma physics, and by extension those to study the comet’s interaction with the surrounding plasma environment, can be divided roughly into three broad numerical approaches (e.g. Ledvina et al. 2008): (1) models that treat all simulated plasma species as one or multiple fluids; (2) models that include some of the plasma species as (computational) particles, so-called ‘hybrid’ codes; (3) models that represent all plasma species using computational particles, so-called ‘fully kinetic’ codes. These categories are summarised in Table 5. Note that these categories are only one non-unique attempt to classify the numerical models used in cometary science. In reality, there is a wide spectrum of models often blending the borders we outlined above. Finally, there are some approaches that do not clearly fit in any of the three categories, such as test-particle or semi-analytical models, whose inputs are sometimes based on outputs of the other three categories.

**Table 5:** Summary table for the most common numerical models and references to works that use them. $\Omega _{gi,e}$ are the gyroradii of the ions and electrons, respectively

Model type	Applicable scale L	Examples
Fluid	$L >\Omega _{gi}>\Omega _{ge}$	e.g. Huang et al. (2018)
Hybrid	$\Omega _{gi}> L >\Omega _{ge}$	e.g. Simon Wedlund et al. (2017), Lindkvist et al. (2018) and Koenders et al. (2016a)
Fully kinetic	$\Omega _{gi}>\Omega _{ge}> L$	e.g. Deca et al. (2019) and Gunell et al. (2019)

Different numerical models provide different advantages and disadvantages. Typically, a less complete/self-consistent physical model translates into a lower computational burden, a more efficient use of computational resources. Some physical mechanisms or fully time-dependent simulations (as opposed to, e.g., a steady-state model with fixed neutral background atmosphere and solar wind conditions) might be almost impossible to implement in an efficient manner without losing self-consistency. This may be due to constraints on available computational resources, and the vast range of time-scales to be resolved (e.g., ions collisionally coupled to neutral gas less than $1~\text{km}\,\text{s}^{-1}$ in speed, versus electron speeds of several $1000~\text{km}\,\text{s}^{-1}$), or to a limited understanding of the underlying physics. In reality, the ‘amount’ of physics included in the model is always a compromise against the amount of available resources. Similarly, by enabling the study of a specific process or physical aspect in greater detail, 1-D and 2-D approaches vs 3-D approaches may bring valuable insights into the physics of cometary plasma environments at reduced cost. In the following paragraphs we attempt to give a brief overview of how numerical models, here mostly 3-D, have been applied in preparation to, during and after the Rosetta mission. We refer the reader to the complementary sections for a more in-depth discussion of the science covered in these works.

As Rosetta intended to accompany 67P throughout all outgassing stages of the comet, predicting the global structure of its plasma environment is key to understanding where to look for the most exciting science. Supported by several decades of experience investigating other comets and solar system bodies [e.g., Gombosi et al. 1996 describe mass-loading near comet 1P/Halley; Jia et al. 2008 model the effect of neutral jets during the Deep Space 1 Comet 19P/Borrelly flyby], (multi-fluid) MHD efforts have paved the way. Needing relatively little computational resources, they can resolve larger physical domains. For example, Huang et al. (2016) modelled a computational domain on the order of $10^{6}~\text{km}$ to investigate the plasma and neutral gas environment of 67P near perihelion. The model extended the single fluid MHD model by Hansen et al. (2007), driven by the need to reproduce effects arising from the gyration of the cometary ions and the deflection of the solar wind protons (Rubin et al. 2014b,a, 2015).

The next step is to include ion dynamics self-consistently. This is achieved with kinetic hybrid models, where ions are treated as statistical macroparticles and electrons as a mass-less charge-neutralising MHD fluid, building on the knowledge accumulated on the plasma environments of various solar system bodies, not necessarily cometary (see e.g. Bagdonat and Motschmann 2002; Kallio et al. 2006; Müller et al. 2011; Holmström 2013). More specifically driven by the Rosetta teams, recent improvements in cometary hybrid models include, e.g., more detailed models of photoionisation, solar wind charge exchange and electron ionisation processes and their respective effects on the creation of large-scale boundaries such as bow shock and cometopause regions (see Simon Wedlund et al. 2017, and Sect. 4.2), the ion dynamics of mass loading (Behar et al. 2016a), and analysis of the observed pickup ion energy spectra and shock-like structures (Lindkvist et al. 2018; Gunell et al. 2018; Alho et al. 2019, 2021). Hybrid approaches have also proven successful to investigate the effects of solar wind variability on the global structure of 67P’s plasma environment (Koenders et al. 2013, 2015, 2016a; Alho et al. 2021), in particular during the weak and intermediately outgassing regimes. Comparing measurements with hybrid numerical simulations, Koenders et al. (2016b) studied the generation of low-frequency waves in the comet-solar wind interaction region, including the “singing comet waves” (see Sect. 2.3.7), showing how they arise from initial cometary pick-up ions perpendicular to the solar wind flow and along the IMF direction, triggering a modified ion-Weibel instability. In the most recent iterations of these models, extended boundary conditions (to include mass-loading, charge-exchange and ion productions over the large upstream region extending up to millions of km in the subsolar direction) as well as asymmetric outgassing driven by the nucleus illumination have been included (Koenders et al. 2013; Alho et al. 2021). Structures that are possible to describe with these models without much loss of ion scale physics include for example bow shock and ion collisionopause down to the diamagnetic cavity and the ambipolar electric field (see Sects. 2.3.2, 4.2, 4.6, and 4.5).

Modelling both electron and ion species as particles, fully kinetic approaches are in theory able to present the most complete physical model. However, it comes at a significant computational cost, restricting present-day global simulations to 67P’s plasma environment during its weakly and moderately active periods. Using four plasma species (solar wind protons and electrons; cometary water ions and electrons), Deca et al. (2017) concluded that the dynamical interaction of a weakly outgassing comet is representative of a four-fluid coupled system and they were able to identify the origins of the warm and suprathermal electron distributions observed by Rosetta. Global particle-in-cell simulations disentangled the convoluted observed electron distributions and showed that the ambipolar electric field temporarily traps and accelerates electrons near the comet without the need for wave-particle or turbulent heating mechanisms (Divin et al. 2020; Sishtla et al. 2019). This also leads to the mechanism responsible for the far-ultraviolet aurora identified at comet 67P (Galand et al. 2020). As no collisions are included, the strict applicability of the method is as yet confined to low-activity comets and/or regions far from the nucleus. Nevertheless, considering the limited size of the collisional domain at 67P (see discussions on exobase and collisionopause in Sects. 2.3.5 and 4.5), the particle-in-cell results can be expected to well represent the comet-solar wind interaction during the Rosetta mission.

A second set of fully kinetic models focused on the plasma environment of the Rosetta spacecraft itself. For example, Johansson et al. (2020) analysed the charging behaviour of Rosetta and Wattieaux et al. (2020) characterised the response of the RPC-MIP instrument to different electron populations. A similar study for RPC-ICA was performed by Bergman et al. (2020). All are crucial to aid our interpretation of the in situ plasma measurements, which in turn are dependent on the spacecraft charging and the local plasma environment (disturbed by the spacecraft, including solar arrays, booms and the relative position of the instruments). Finally, note that a fully kinetic description for the complex electron dynamics can provide an effective electron closure relation and identify where an isotropic single-electron fluid Ohm’s law approximation can be used, and where it fails (Deca et al. 2019). The latter can then be used as input for less computationally demanding numerical approaches.

As mentioned above, there are a multitude of numerical approaches that do not clearly fit in any of the categories outlined above. Most prominently in these group are semi-analytical models and test-particle codes. The latter have been successful to characterise, e.g., the solar wind ion cavity (Behar et al. 2017) and suprathermal electrons near the nucleus of comet 67P (Madanian et al. 2016). Direct simulation Monte Carlo methods have been used to characterise the observed non-uniformity of the cometary neutral coma (Bieler et al. 2015b; Fougere et al. 2016a; Combi et al. 2020).

## Large Scale Structures

**Fig. 34:**
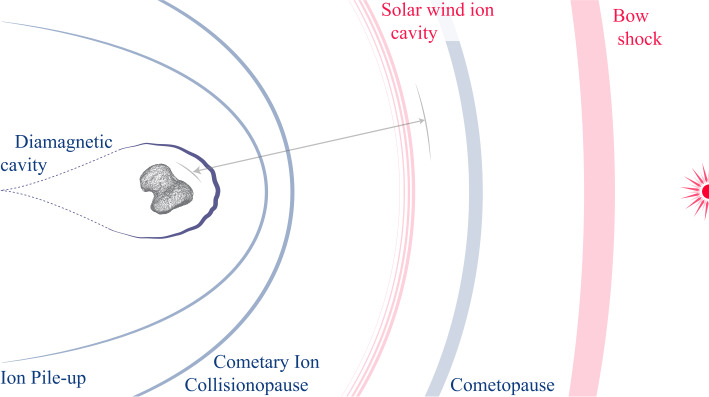
Illustration of the boundaries formed by the interaction of the solar wind with the coma that have been observed during spacecraft flybys of comets. All of these boundaries are thought to be permanent features of the interaction when the outgassing rate is high enough. The grey shaded region is the portion of the comet solar wind interaction explored by Rosetta between 2015 May and December

The interaction of the solar wind with comets is unique compared to how the solar wind interacts with unmagnetized planetary atmospheres. This is because the high neutral outgassing rate of comets and the free expansion of the atmosphere causes this interaction to begin at great distances from the comet through the process of mass loading of the solar wind. The result is large scale structures in the form of boundaries, regions, and electric fields that have been observed by spacecraft flybys of comets and by Rosetta during its extended escort of 67P. Boundaries observed by a spacecraft at a comet can be permanent features, solar wind and interplanetary magnetic field (IMF) boundaries, or small-scale transient features created by instabilities or waves (Cravens 1989). Figure 34 illustrates the relative positions of the boundaries discussed in the following sections.

### Electric Fields

Large scale plasma phenomena can usually be described using a fluid model. The main assumption needed is that the characteristic scale size of the phenomenon being investigated is much larger than an ion gyroradius. On smaller scales, either concerning physics such as the formation of pick-up ring distributions or where the characteristic gradients in the system are smaller than an ion gyroradius, electric fields become important. The same is true in the absence of a background magnetic field, as e.g. in the diamagnetic cavity.

When a comet plasma cloud is small compared to a pick up ion gyroradius, ions may be locally unmagnetised. Locally produced ions will move along the solar wind electric field on these scales. We know from conservation of momentum and observations that the solar wind will be deflected in the direction opposite to the cometary ions (see also Sect. 2.3.1 and Behar et al. 2016b, 2018b; Broiles et al. 2015). To understand the local plasma dynamics on this scale, it is usually helpful to look at the solar wind electric field in the local comet reference frame. The same is true for planet systems that are small compared to the pick up ion gyroradius such as Mars, where a plume of pick up ions is seen along the solar wind electric field direction (Dong et al. 2015).

**Fig. 35:**
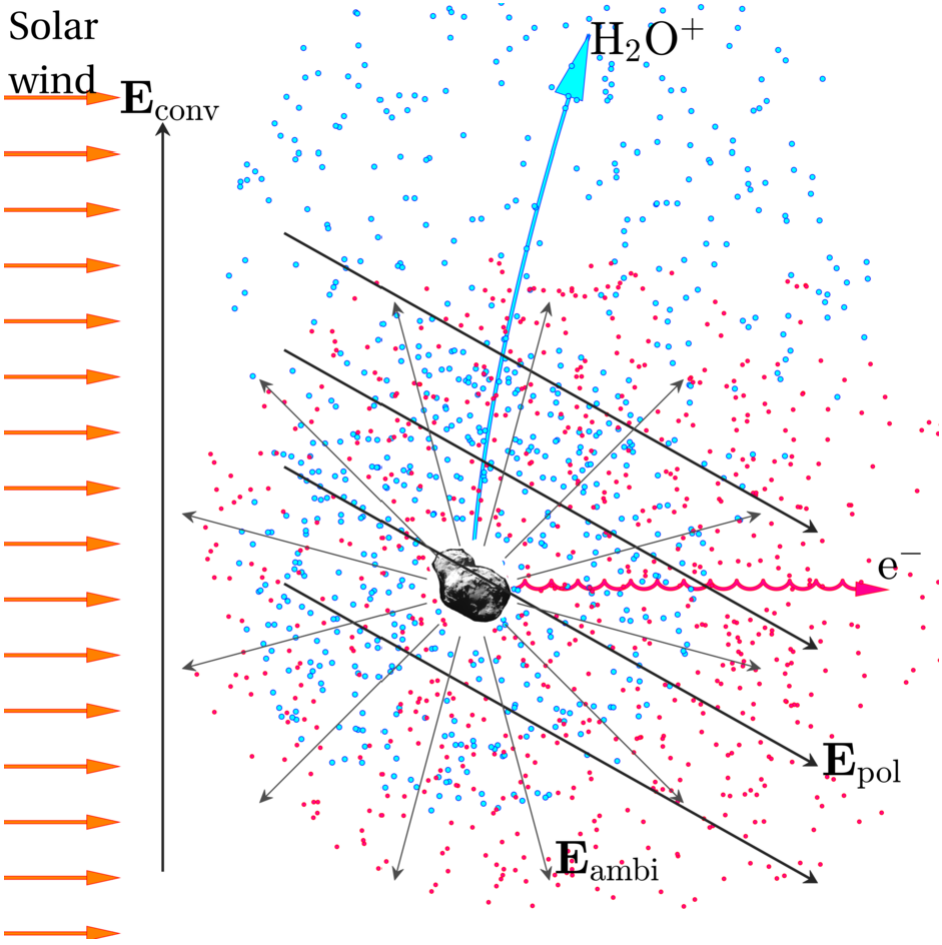
Illustration of the electric fields in the inner part of the coma of a comet that has not developed a diamagnetic cavity. From (Gunell 2020)

Another important electric field comes into play when the newly added ions are no longer just a small perturbation to the solar wind flow. The newly born ions and electrons move in different directions along and against the solar wind electric field, so if the solar wind cannot easily displace the accumulating charges along the field line a polarisation electric field will build up, preventing further charge accumulation (Brenning et al. 1991; Nilsson et al. 2018). In particular this electric field arises in the case of unmagnetised ions and magnetised electrons, as was typically the case at comet 67P during the Rosetta mission. This is illustrated in Fig. 35, where $\mathbf{E}_{\mathrm{pol}}$ shows the polarisation electric field. The effect of the polarisation electric field has been modelled for barium release experiments (Brenning et al. 1991), a model later adapted to the situation at comet 67P (Nilsson et al. 2018). Gunell et al. (2019) further studied the role of the polarisation electric field using electrostatic PIC simulations. Such small scale plasma clouds with polarisation electric field effects have also been discussed based on MAVEN observations from Mars (Halekas et al. 2016). One prediction of the model is a significant anti-Sunward flow of the pick up ions, rather than along the inferred solar wind electric field direction. This is in agreement with observations (Nilsson et al. 2015b, 2017). One can regard it as that the frozen-in electrons drag the ions with them, an interpretation also used by Dubinin et al. (1993) describing a similar situation in the magnetotail of Mars.

A simple model of the polarisation electric field (Nilsson et al. 2018) predicts that the electric field should be quite uniform inside the cometary plasma cloud. If so, the energy spectra of cometary ions moving along the electric field can directly be related to the distance between the measurement and the production region of the ions. The energy spectra can thus be used to remotely probe the plasma cloud structure. Nilsson et al. (2018) suggested that an abrupt increase of fluxes at energies above 1 keV could be related to the bow shock. Such a remote detection of the large scale bow shock close to perihelion was later reproduced in a hybrid model by Alho et al. (2019). The shape of the energy spectra thus reflects how the production of ions changes with distance, and abrupt changes in the energy spectra corresponds to abrupt changes in either production or the electric and magnetic fields and associated ion transport.

As the cometary plasma cloud grows larger and denser, the solar wind electric field is more efficiently shielded from the interior of the plasma cloud (Nilsson et al. 2018). Locally produced pick up ions are then less accelerated, possibly explaining the lower ion energies observed inside the comet magnetosphere around perihelion (Nilsson et al. 2017). This effect occurs at similar times and in regions where collisions also start to play a more significant role (see also Sect. 4.5). The stronger shielding of the solar wind electric field should however not lead to any clear boundary in the plasma, and accelerated ions are still present, just at lower energies.

Closer to the nucleus newborn, hot photoelectrons and a strong density gradient give rise to a significant ambipolar electric field accelerating ions radially outward (Odelstad et al. 2018; Berčič et al. 2018) ($\mathbf{E}_{\mathrm{ambi}}$ in Fig. 35). In a sense this is similar to the polarisation electric field, with the hot photoelectrons dragging the ions with them. However, while the radially directed ambipolar field is caused by the electrons having a higher temperature than the ions, the polarisation field is a secondary field caused by electrons and ions moving in different directions as a result of the solar wind convective electric field. Figure 35 illustrates the directions of the three different contributions to the electric field and the different scales of electron and ion gyroradii, which causes the charge separation behind the polarisation electric field. The presence of several distinct electron populations also means that the polarisation electric field accelerates some electrons inward (Madanian et al. 2017; Deca et al. 2017). The main source of hot electrons is believed to be photoelectrons due to EUV ionisation and secondary electrons due to electron impact ionisation, but additional heating is needed to explain the highest observed electron temperatures (see Sect. 2.3.3).

Rosetta observations showed that low energy ions observed close to the nucleus are moving away from the nucleus with a velocity well above that of the neutral gas (Odelstad et al. 2018). The Rosetta observations were made also well inside the collisionopause illustrated in Fig. 34, (see also Sect. 4.5). The ions can thus attain a speed of typically 2 to 4 km/s also inside the collisionopause, which in turn affects the collision cross section which is energy dependent and thus also the position of the collision dominated region (Mandt et al. 2019).

Rosetta observations also showed that electrons in the inner coma exhibit an excess of their suprathermal component as compared to what is expected from local production of photo electrons (Broiles et al. 2016a; Myllys et al. 2019), which result from inward electron acceleration by this ambipolar electric field (Madanian et al. 2017; Deca et al. 2017).

In the Rosetta observations, the most common flow direction for cometary ions with an energy below about $60~\text{eV}$ as measured by RPC-ICA (Berčič et al. 2018; Nilsson et al. 2017) was a radial outflow in $\mathrm{{Y_{CSEQ}}}$–$\mathrm{{Z_{CSEQ}}}$ plane with a significant anti-Sunward component. This represents a combination of the influence of the polarisation electric field (anti-Sunward) and the ambipolar electric field (radial expansion). Note that the energy limit given here is after acceleration by the typically negative spacecraft potential (Odelstad et al. 2017) which means that the typical ion energy range when compensating for the spacecraft potential (Bergman et al. 2020) was in the energy range typically up to about 40 eV.

Other regions where quasi-stationary electric fields may be important are at sharp boundaries. Of the main boundaries shown in Fig. 34 the bow shock (Sect. 4.2) and the diamagnetic cavity (Sect. 4.6) are the ones most likely to exhibit electric field signatures.

In a coma the ambipolar electric field (see also Sect. 2.3.2) is present as soon as electron collisions are low enough to allow for a hot electron component. Ambipolar acceleration has been observed inside the diamagnetic cavity (Odelstad et al. 2018). At the diamagnetic cavity boundary, which is rather sharp at about 25 km width (Goetz et al. 2016a; Neubauer 1988), further transients can be expected due to the discontinuity of the mobility of electrons and ions from the field-free to the magnetised region outside the cavity. Studies of this are ongoing, but the effect of a sometimes abruptly changing spacecraft potential is yet to be fully taken into account for the interpretation of Rosetta data (Masunaga et al. 2019; Bergman et al. 2020; Johansson et al. 2020).

### Bow Shock

Shocks form in a plasma flow upstream of obstacles, if the relative velocity of the plasma and the obstacle exceeds the characteristic speed of waves in the plasma. The compressional wave which forms in front of the obstacle steepens when more plasma comes in from upstream (see for example, Balogh and Treumann 2013; Burgess and Scholer 2015). A multitude of instabilities and wave modes are possible. Typically though, bow shocks grow from fast magnetosonic waves in the supermagnetosonic solar wind. The dissipation that causes heating of the solar wind as it passes the shock is caused by secondary instabilities leading to waves on smaller and faster scales, involving both ions and electrons.

Early theory showed that hydrodynamic (Biermann et al. 1967) and magnetohydrodynamic (Flammer and Mendis 1991) models of mass-loading break down already when the fraction of incorporated cometary ions reach a few per cent, unless a discontinuity corresponding to a bow shock is introduced. The agreement between these models and observations of the bow shock of the highly active comet 1P/Halley is reasonable (Neubauer et al. 1986).

Mass-loading slows down the solar wind upstream of the shock, leading to a lower Mach number than in the undisturbed solar wind or at planetary bow shocks. The magnetosonic Mach numbers of the Giotto bow shock crossings of comet 1P/Halley were computed by Coates et al. (1990) and were found to be between 1.1 and 1.8 inbound and between 1.6 and 1.7 outbound. This would make the shock marginally supercritical, but criticality also depends on the shock normal angle to the magnetic field (Balogh and Treumann 2013). One would in any case expect cometary shocks to have more subcritical properties than the bow shocks at planets. Subcritical shocks rely more on dissipation at the shock itself, whereas supercritical shocks reflect a significant fraction of the solar wind particles back upstream, which leads to instabilities, wave growth, and dissipation of energy in the foreshock region. In a single fluid MHD description, shocks fulfil the Rankine-Hugoniot conditions. In a multi-component plasma these conditions need to be modified (Motschmann et al. 1991b,a), and the different ion species behave differently. This changes further when kinetic effects are included (Fahr and Siewert 2015).

Comet bow shocks are more diffuse and wide than the bow shocks known from planets. This was found when the International Cometary Explorer (ICE) spacecraft flew by comet 21P/Giacobini-Zinner in 1985 (Smith et al. 1986). Bame et al. (1986) observed that the transitions were smooth and gradual in magnetic field and particle observations, but the wave instrument on ICE measured sharp boundaries where the wave activity increased in the same way as at planetary bow shocks, and an intense broadband spectrum of waves indicative of energy dissipation was found (Scarf et al. 1986b). As expected for shocks, the width of the bow shock was not proportional to the distance to the comet for comets 21P/Giacobini-Zinner and 1P/Halley. The widths were approximately the same, $(3\text{--}4)\times 10^{4}~\text{km}$, whereas the cometocentric distance to the shock differed by an order of magnitude. It was $1.27\times 10^{5}~\text{km}$ at comet 21P/Giacobini-Zinner and $1.15\times 10^{6}~\text{km}$ at comet 1P/Halley (Neubauer et al. 1986). The results obtained by the two Vega spacecraft were similar to those of Giotto (Gringauz et al. 1986a). Both Vega 2 and Giotto passed through a quasi-parallel shock on the outbound legs of their flybys (Galeev et al. 1986; Coates 1995).

The observations of comet 26P/Grigg-Skjellerup, visited by the Giotto spacecraft in 1992, yielded similar results with sharp transitions in both field and waves when the spacecraft was outbound. On the inbound leg only the wave measurements showed a sharp transition, while the increase in magnetic field was gradual (Neubauer et al. 1993b). It was later seen in models and observations at comet 67P/Churyumov-Gerasimenko that bow shocks can be very asymmetric at intermediate cometary outgassing rates. The outgassing rate at comet 26P was estimated to $6.7\times 10^{27}~\text{s}^{-1}$ (Neubauer et al. 1993b), which is similar to comet 67P at perihelion and above the outgassing rate during the infant bow shock observations at comet 67P (see the outgassing rates in Fig. 3 in Sect. 2.1.1). This would place comet 26P between comet 67P and comets 21P/Giacobini-Zinner and 1P/Halley both in terms of outgassing rates and in bow shock symmetry. Asymmetries in bow shock thickness may also be caused by outgassing asymmetries as shown by simulations (Huang et al. 2016).

The last of the pre-Rosetta in situ bow shock observations happened in 2001 at comet 19P/Borelly. The Deep Space 1 spacecraft detected a bow shock $1.47\times 10^{5}~\text{km}$ from the nucleus (Richter et al. 2011). The observed magnetic compression ratio was 2.5, and the magnetic field fluctuations were seen to increase as the spacecraft moved into the region downstream of the shock.

Before Rosetta arrived at comet 67P, simulations predicted that the standoff distance of the bow shock would be a few thousand kilometres (Koenders et al. 2013, 2015). Simon Wedlund et al. (2017) performed a series of hybrid simulations in which first only photo-ionisation was included and then also electron impact ionisation and charge-exchange processes. The result was that with each addition of an ionisation process, the bow shock moved farther upstream up to a maximum standoff distance of more than $6000~\text{km}$. Rosetta ventured out to a cometocentric distance of about $1500~\text{km}$ during its dayside excursion in September 2015. Given that this distance was much below the predicted standoff distance, it was no surprise that the bow shock was not found, and it was thought that Rosetta had not observed the bow shock at all during the mission (Mandt et al. 2016; Simon Wedlund et al. 2017). In fact the magnetic field strength did not decrease significantly even at $1500~\text{km}$ (as compared to $100~\text{km}$), indicating that Rosetta was not near the bow shock, where lower field values are expected (Goetz et al. 2017). Recently, the non-detection of a bow shock was vindicated by Alho et al. (2021) who took into account a more realistic asymmetric outgassing based on the semi-empirical model of Hansen et al. (2016) and an extended mass-loading boundary condition (see also Sect. 3.3). These authors estimated with a kinetic hybrid plasma model that the bow shock subsolar standoff distance was increased by a factor 2 to 3 with respect to non-asymmetric outgassing conditions, reaching about $18{,}000~\text{km}$ for 67P around perihelion. They showed also that simulated pickup ion observations deep in the coma display spectral features in energy that may be interpreted with the simulations as the presence of shock-like structures far upstream of the spacecraft. Both asymmetric outgassing and IMF clock angle were found to drive these spectral features.

The dayside excursion took place when the comet was close to perihelion, and the simulations mentioned above all concerned conditions corresponding to a comet near perihelion. However, far away from the Sun, the comet has only a very thin atmosphere, and its size is smaller than the proton gyroradius ($\sim 10^{3}~\text{km}$ for a solar wind proton in the comet frame of reference and a 4 nT magnetic field). Hence, one would not expect a comet to have a bow shock at large heliocentric distances. Between the no-shock situation far away from the sun and the fully developed shock at perihelion, the bow shock forms. Lindkvist et al. (2018) performed hybrid simulations of comet 67P, comparing two different ionisation rates. In the simulation with the lower ionisation rate, a very asymmetric “shock-like structure” formed, extending far into the $z<0$ plane in CSE coordinates. In the CSE coordinate system the $z$ axis is parallel to the convectional electric field of the solar wind. The purpose of the simulations by Lindkvist et al. (2018) was to study energy conversion at the comet. It was found that the shock-like structure acts as an electromagnetic generator in the same way as Earth’s bow shock. In a generator $\vec{E}\cdot \vec{J}<0$ and energy is transferred from the particles to the fields. The shock-like structure also has the other properties of a collisionless shock, a rapidly increasing magnetic field, and a slowing down and heating of the solar wind, to the extent it can be modelled with the available resolution and in a hybrid simulation that does not include electron physics.

Gunell et al. (2018) found that under specific circumstances heated protons could be detected near the nucleus. Because of the similarity of these observations with numerical simulations, they introduced the concept of a highly asymmetric and unstable “infant bow shock” to explain the presence of these protons. Goetz et al. (2020a) performed a statistical study of the complete data set for the whole mission, and found that this shocked solar wind plasma had been observed on 152 days.

**Fig. 36:**
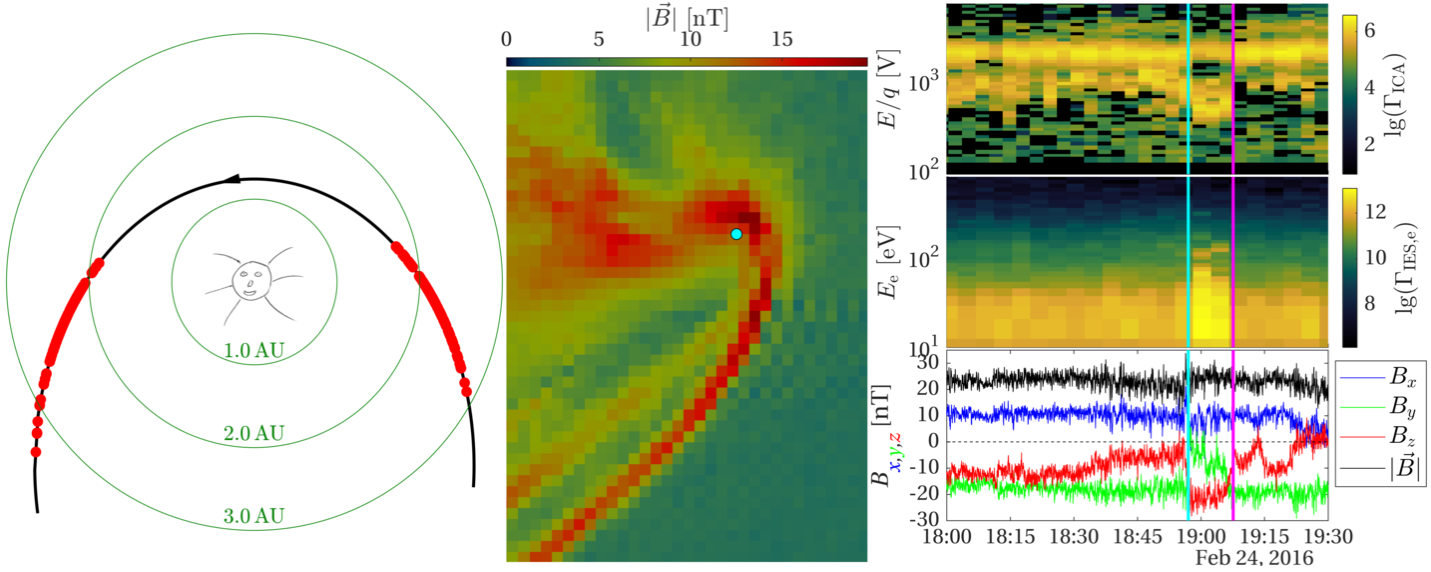
The infant bow shock at comet 67P. In the left-hand panel the red circles mark the position of comet 67P on each of the 152 days on which shocked solar wind plasma was observed (Goetz et al. 2020a). The second panel shows the magnetic flux density magnitude in a hybrid simulation of the infant bow shock. The light blue circle marks the position of the nucleus. The three panels on the right-hand side show from the top: the energy spectrum of the light ions, the electron energy spectrum, and the components and magnitude of $\vec{B}$ as observed by the Rosetta spacecraft (Gunell et al. 2018). The vertical light blue and purple lines mark when the spacecraft entered and exited the region with shocked plasma in an infant bow shock encounter. From (Gunell 2020)

Figure 36 shows a summary of these observations. The left-hand panel shows where along the comet orbit the observations took place. The first occurrence was at a heliocentric distance just below 3 AU. When the comet got closer than 1.7 AU from the Sun the infant bow shock sightings ceased. This is consistent with the bow shock moving outward and sunward as the outgassing and ionisation increased, and Rosetta was then no longer in the region with shocked plasma. The infant bow shock returned after perihelion. It is seen in Fig. 36 that the bow shock sightings are shifted somewhat toward larger heliocentric distances on the outward leg of the comet’s journey when the outgassing rate was higher (Hansen et al. 2016). It was found by Goetz et al. (2020a) that the infant bow shock is seen more often at intermediate outgassing rates.

The middle panel of Fig. 36 shows the magnitude of $\vec{B}$ in the $x$–$z$ plane in a hybrid simulation, where the $x$ axis is directed toward the Sun and the $z$ axis parallel to the solar wind electric field. It is seen how the infant bow shock extends far into the negative $z$ half plane and how the magnetic field increases at the shock, and continues to oscillate on the downstream side. On the right-hand side of Fig. 36 are three panels showing an example of Rosetta crossing the infant bow shock. Here, the spacecraft moves with respect to the infant bow shock because the shock turns due to a change in the solar wind magnetic field direction. This also changes the direction of the electric field, and hence of the $z$ axis in the middle panel of Fig. 36. An example of when this happened is marked by the light blue vertical line in Fig. 36 (see Gunell et al. 2018, for a detailed description).

An infant bow shock can be seen in figures from the simulation by Koenders et al. (2016a) although at the time it was not called by that name. The simulation was used to model the close flyby of comet 67P on 28 March 2015. Rosetta was close to the nucleus and the infant bow shock farther out, which is why it was not observed that day. Edberg et al. (2016b) studied the impact of corotating interaction regions on comet 67P, but they also found another feature. Edberg et al. (2016b) describe it thus: “On 24 December 2015, when the magnetic field orientation has changed, the solar wind flux is decreased and the suprathermal electrons increased significantly. It is possible that these signatures also indicate the crossing of a plasma boundary, which builds up as the solar wind dynamic pressure increases.” This can now be identified as one of the first encounters of the infant bow shock and it is part of the infant bow shock database used in the statistical study by Goetz et al. (2020a).

Since the Rosetta spacecraft was located downstream of the bow shock when the comet was less than 1.7 AU from the Sun, no in situ observations could be made for a considerable length of time. A method for remote detection of a bow shock was suggested by Nilsson et al. (2018), who observed that if cometary ions are accelerated by a constant electric field, the energy at which they reach the spacecraft is proportional to the distance to the place where they were created by ionisation. The ion energy spectrum is therefore determined by the $1/r^{2}$ slope of the ionisation source. Nilsson et al. (2018) applied this method to Rosetta data from 16 June 2015 at approximately 1.4 AU and found the $1/r^{2}$ relationship to hold up to an energy of $\sim1~\text{keV}$, which corresponded to a distance of 4000 km, and they predicted that that was the position of the bow shock. The method has been evaluated by simulations, which yield consistent results (Alho et al. 2019).

The physics of collisionless shocks is complicated, and it involves a number of different types of waves on both large and small scales. A complete understanding of it would require both observations and computer simulations that treat both electrons and ions kinetically and resolves all relevant scales (Balogh and Treumann 2013). At comets the situation is complicated further by the presence of several ion species, and also the neutral atmosphere has a significant influence at least for some regimes. The way forward from an observational point of view is to make simultaneous measurements at multiple points in space as in the upcoming Comet Interceptor mission (Snodgrass and Jones 2019), which will make a fast fly-through of a cometary plasma. To obtain a truly detailed view of the bow shock of a comet one would need a multi-spacecraft mission that accompanies the comet over an extended period of time (Goetz et al. 2021). The possibility of learning about cometary bow shocks by conducting laboratory experiments here on Earth is discussed briefly in Sect. 3.1.3.

### Solar Wind Ion Cavity

**Fig. 37:**
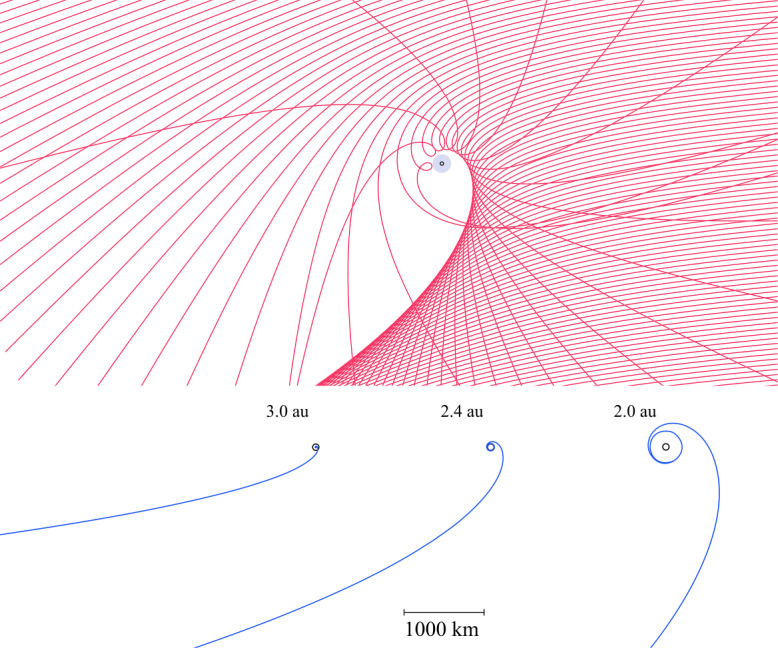
Analytical trajectories of solar wind protons, Sun to the right and upstream magnetic field directed upward, in the same plane. Around the nucleus at the centre, a region is created in which solar wind ions cannot penetrate. According to the model, these trajectories simply scale with the activity and the heliocentric distance. From Behar et al. (2018a), reproduced with permission ©ESO

From May to December 2015, that is to say, several months before and after perihelion, Rosetta was in a region which was mostly free from solar wind ions – the solar wind ion cavity (Nilsson et al. 2017; Behar et al. 2017). Mass loading of the solar wind leads to magnetic pileup (see Sect. 4.4) and the increased field causes deflection of solar wind ions. On large scales and for a low-to-medium outgassing activity, we have reviewed in Sect. 2.3.1 the theoretical and observational understanding of the ion dynamics within the coma. What happens closer to the nucleus, or similarly when the comet draws closer to the Sun? Still considering the electric field as mostly characterised by its motional component, we can further characterise the local ion dynamics, given the ion densities and the magnetic field strength. Using simple analytical models for these, a semi-analytical model of the global dynamics of solar wind protons for a given heliocentric distance can be derived (Behar et al. 2018a), and results in the trajectories shown in Fig. 37. The trajectories shown are monoenergetic test particles moving in a magnetic field proportional to $r^{-2}$. Other magnetic field models are discussed in Sect. 4.4. We find a solar wind deflected in the direction opposite to the upstream electric field, as previously discussed in Sect. 2.3.1, resulting in an asymmetrical flow.

One of the main aspects of this model is the resulting inner-region in which solar ions cannot penetrate: the solar wind ion cavity. Previous cometary missions did observe a region free of solar wind ions, for more active comets, close to the Sun. At 67P, Rosetta observations showed increasingly deflected solar wind ions, and when the deflection reached values of $90^{\circ}$ and more, they were observed more scarcely, with lower densities. They finally disappeared from the observations, to reappear after perihelion (Behar et al. 2017), a result seen in Fig. 11 of Sect. 2.3.1, first and second upper rows. Close to the ion cavity, protons were observed with very large deflections, before disappearing from the data.

The deflection angle estimated by the model could be compared to observed deflections, on the time scale of the mission, or of one of the two excursions, namely the night-side excursion (Behar et al. 2018a). These successfully estimated deflection angles within an extended region of the coma validate the way the solar wind gyrates within the coma, being repelled from the inner-most region – the ion cavity – through this global kinetic interaction.

With a more careful look and during the time the probe was within the solar wind ion cavity, some protons were observed during the dayside excursion, which brought the spacecraft to the very boundary of the solar wind ion cavity at $800~\text{km}$, simultaneously with the interaction between the coma and a CME (Edberg et al. 2016a).

Simon Wedlund et al. (2019b) posed the question of whether charge exchange between solar wind ions and cometary neutrals could be responsible for the decrease in solar wind ion flux that is observed when the spacecraft enters the solar wind ion cavity.

The solar wind ion cavity and the diamagnetic cavity are two different structures, with different spatial scales, involving very different physics. While the ions are deflected and diverted from the solar wind cavity, electrons keep advecting the magnetic field deeper within the ion cavity (Deca et al. 2017). As a result, the diamagnetic cavity is entirely independent and almost always within the ion cavity.

While the solar wind ions are excluded from the solar wind ion cavity, the solar wind magnetic field and pick up ions created upstream permeate the region downstream of the solar wind ion cavity boundary until the diamagnetic cavity boundary. Williamson et al. (2020) looked at the momentum flux continuity and plasma pressure balance over the Rosetta mission. They found the pressure on both sides of the solar wind ion cavity boundary to be approximately equal, as can be expected. The pick up ion momentum flux inside the solar wind ion cavity contributes the anti-sunward momentum flux of the solar wind outside. Essentially the pick up ions replace the solar wind ions. A further aspect of this was reported by Nilsson et al. (2020) who noted that the pick up ions just outside the boundary as well as inside the solar wind ion cavity were deflected in a similar manner to the solar wind. The pick up ions were moving against the inferred solar wind electric field direction. This is consistent with these ions carrying the momentum into the solar wind ion cavity and providing momentum to newborn ions inside the cavity. The description of the deflected solar wind in Sect. 2.3.1 holds here as well, with the pick up ions gyrating around the common centre of mass of the pick up ions and the locally born cold ions. The solar wind ion cavity boundary is thus not a significant boundary in terms of energy and momentum flux from the solar wind to the inner part of the coma.

### Magnetic Field Structure and Tail

**Fig. 38:**
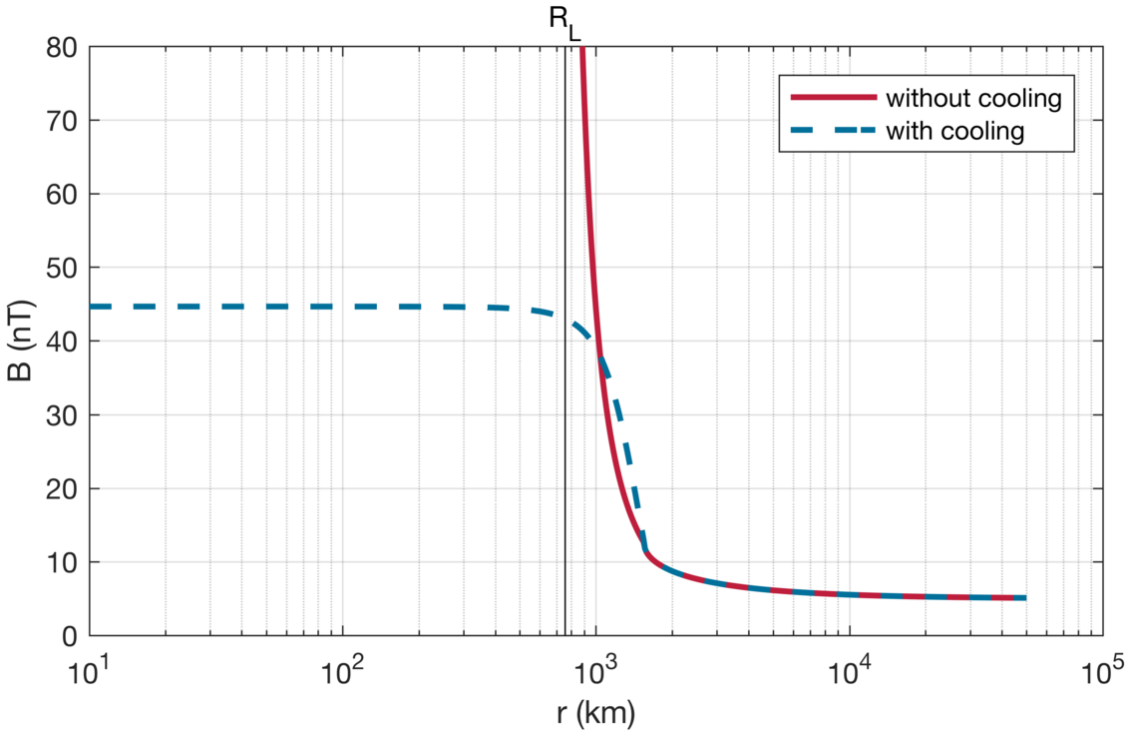
Radial magnetic field profile for the two models as applied to comet 67P/Churyumov-Gerasimenko. From Goetz et al. (2017), Fig. 1

The interaction of the IMF carried by the solar wind and an outgassing comet leads to the formation of an induced magnetosphere through mass loading. The neutral gas coming from the cometary nucleus gets ionized and picked up by the IMF. This mass loading of the solar wind will slow it down and a simple 1D MHD model, presented by Biermann et al. (1967) and Flammer and Mendis (1991), can give an analytic solution of the solar wind velocity by combining the continuity, momentum and energy equations (see also Goetz et al. 2017):

21 $$\begin{aligned} u_{x}(x) =&\, 2 (f + 1) (M(x) + \rho _{\infty} u_{\infty}) \times \\ &\left [ (f+2) \rho _{\infty} u_{\infty}^{2} \pm \sqrt{(f+2)\rho _{\infty}^{2} u_{\infty}^{4} - 4(f+1) (M(x) + \rho _{\infty} u_{\infty}) \rho _{\infty} u_{\infty}^{3} } \right ], \end{aligned}$$

where $\rho _{\infty}$ and $u_{\infty}$ are the undisturbed solar wind density and velocity, $f$ is the number of degrees of freedom of the ions, and $M(x)$ is the mass source which is given by:

22 $$\begin{aligned} M(x) = \int _{-\infty}^{x} \frac{m_{c} Q \nu _{0}}{4 \pi u_{n} r^{2}} \, \mathrm{d}r, \end{aligned}$$

which is the integrated column density from the Haser (1957) model, with $Q$ the outgassing rate, $m_{c}$ the cometary ion mass and $u_{n}$ the outflow velocity of the neutrals and $\nu _{0}$ is the photoionisation frequency. The source is expected to emit radially symmetrically, and thus a $r^{-2}$ fall off of the neutral density. The magnetic field is then simply given by:

23 $$\begin{aligned} B_{y}(x) =\frac{u_{x\infty} B_{y\infty}}{u_{x}(x)}, \end{aligned}$$

with $B_{y\infty}$ the magnetic field of the solar wind. This description has a problem for the location $x_{l}$ where $u_{x}(x_{l})$ becomes complex and the magnetic field $B_{y}(x) \rightarrow \infty $ and the bow shock is created (see Sect. 4.2). A solution to the problem of the flow behind the shock was given by Galeev et al. (1985), when one assumes there is cooling of the plasma through charge exchange at a rate of $\alpha $ (for details, see also Goetz et al. 2017). This then results in a finite magnetic field behind the shock, as shown in Fig. 38.

With this 1D model one can get the magnetic field magnitude in the cometosheath along the Sun-comet line, but it does not describe the 2D/3D structure of the induced magnetosphere. The model needs to be adapted in that the mass loading source falls off radially away from this line. This means that the mass loading further away is less, and thus the braking of the solar wind and IMF will be less. Thereby, the magnetic field will be draped around the active nucleus, as described by Alfvén (1957).

In order to describe the actual situation at a comet, the dynamics of the IMF need to be incorporated. The IMF is usually split-up into four or six sectors, in which the radial component of the magnetic field changes sign. Thereby, differently directed magnetic field will be draped around the active nucleus. Depending on flow velocity of the magnetoplasma and the diffusion time of the magnetic field through the plasma, there can then be a layering of these field lines in front of the nucleus, creating “nested draping.” This was first observed during the flybys of comet 1P/Halley by VEGA 1 (Riedler et al. 1986) and Giotto (Raeder et al. 1987). Indeed, for the Giotto flyby, Raeder et al. (1987) were able to find the corresponding regions during ingress and egress, showing the actual “onion layers” of the draped magnetic field.

**Fig. 39:**
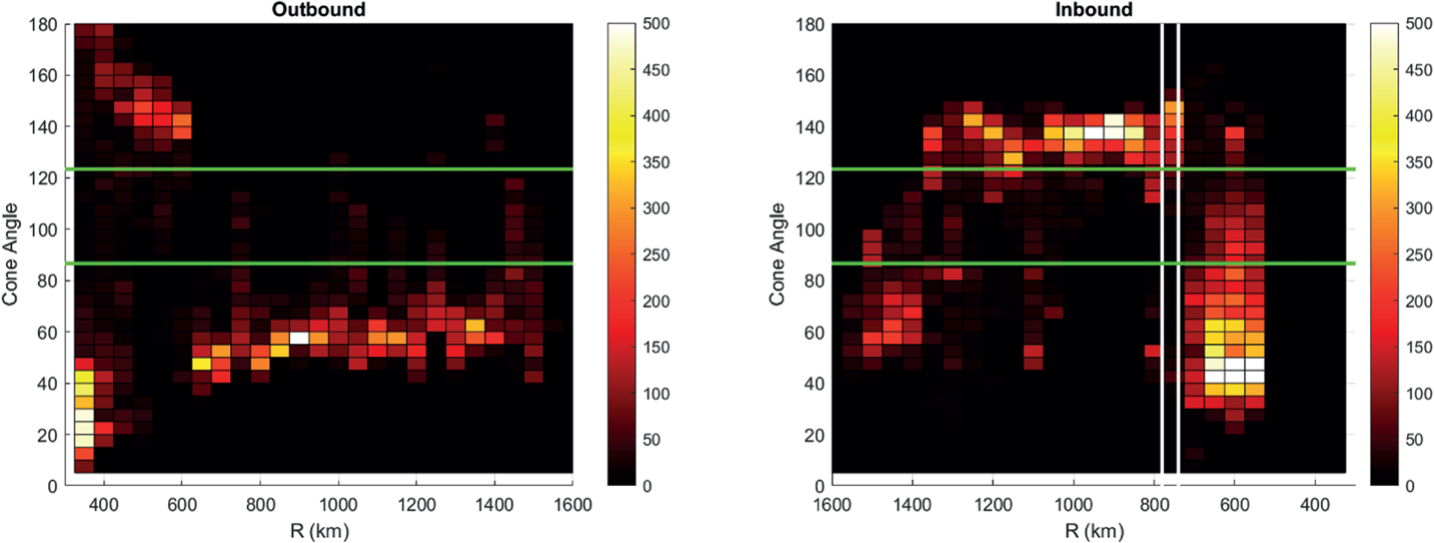
Two-dimensional histograms of the cone angle of the magnetic field along the Rosetta orbit for the outbound (left) and inbound (right) leg. The green lines show the aberrated Parker spiral angles. From Volwerk et al. (2019), reproduced with permission ©ESO

The flybys of comet 1P/Halley took less than 30 minutes, and thus the magnetic picture that was obtained of the upstream region was a snapshot. This was quite different for the dayside excursion of the Rosetta spacecraft at comet 67P/Churyumov-Gerasimenko. In this case the spacecraft took an upstream excursion of $\sim 20$ days up to a distance of $\sim 1500~\text{km}$ from the nucleus. Volwerk et al. (2019) determined the cone angle of the magnetic field during the excursion, shown in Fig. 39 as a function of distance from the nucleus. In the case of nested draping, the cone angle, defined as:

24 $$\begin{aligned} \theta _{\mathrm{co}} = \tan ^{-1} \left \{ \frac{ \sqrt{ B_{y}^{2} + B_{z}^{2}} }{B_{x}} \right \}, \end{aligned}$$

where $\theta _{\mathrm{co}} = 0^{\circ}$ is purely sunward directed field and $\theta _{\mathrm{co}} = 180^{\circ}$ purely anti-sunward. In Fig. 39 it is clear that the cone angle switches between small values, below the abberated Parker spiral (Parker 1958) (green lines), to large values above the abberated Parker spiral. Through the motion of the comet an abberation of $\sim 15^{\circ}$ is created. So, indeed, during the outbound leg there are three regions of differently directed magnetic field. On the inbound leg there is a change in direction at $R \approx 1400~\text{km}$, which demarks a region that did not exist at that location during the inbound leg. This means that the draping moves faster towards the nucleus than the spacecraft is moving. This is called “dynamic draping.”

This means that the upstream structure of the magnetic field looks slightly different depending on the velocity of the spacecraft, the static picture that is obtained from a fast flyby is put into perspective by the slow upstream excursion.

Closer to the active nucleus there is another draping effect that needs to be taken into account. Koenders et al. (2016a) showed that the draping of the magnetic field close to the comet at intermediate gas production rates was mainly in the anti-direction of the solar wind convective electric field. This is caused by Newton’s second law; the newly created ions are accelerated in the direction of the convective electric field, and as a reaction the solar wind ions will move in the opposite direction (see also Broiles et al. 2015), dragging along the frozen in magnetic field. This was observed on 28 March 2015 (see Koenders et al. 2016a, Fig. 2) when Rosetta had a closest approach of $\sim 15~\text{km}$. Naturally, this effect starts to become very important when the pick-up density starts to become similar to the solar wind density.

Following the mass-loaded field lines further downstream the cometary ion tail is reached, where the field lines stretch behind the comet, creating a double lobed (induced) magnetotail as described by Alfvén (1957). The first evidence for this magnetotail structure was given by the ICE spacecraft, on 11 September 1985, when it crossed the tail of comet 21P/Giacobini-Zinner at distance of $\sim 10^{4}~\text{km}$ (Smith et al. 1986).

Slavin et al. (1986, Fig. 1) showed the magnetic field super-imposed on a ground-base image of the comet in a band around the $\text{H}_{2}\text{O}^{+}$ 7025 Å line. Before the bow shock crossing there are small variations in the magnetic field vectors, pointing sunward, with a strength varying up to 10 nT. After the bow shock is crossed, the magnetic field is much stronger and pointing anti-sunward, decreasing in strength as ICE moves towards $Z'_{\mathrm{CSE}} = 0$. Near $Z'_{\mathrm{CSE}} \approx 1000~\text{km}$, the field is very weak, and then turns sunward again, and increases in strength. The field decreases in strength again towards the bow shock and after exiting the field is first anti-sunward and then returns sunward again like before the encounter. This encounter clearly showed the double lobed draped magnetic field of the induced magnetotail.

The second investigation of the tail region of a comet was performed by Rosetta at comet 67P/Churyumov-Gerasimenko. In this case the spacecraft moved up to $\sim 1000~\text{km}$ down tail of the nucleus in the period from 24 March until 10 April 2016 and only the very near-region was studied (Volwerk et al. 2018). In order to study the draping of the field, the cone-angle $\theta _{\mathrm{co}}$ was calculated. Volwerk et al. (2018) showed that the peak in the cone angle is between $60^{\circ}$ and $80^{\circ}$, which means that the “draped” magnetic field is more cross tail than along the Sun-comet-line as one would expect.

The explanation for the “cross-tail” direction of the magnetic field can be found in the deflected solar wind magnetic field (Koenders et al. 2016a) as discussed above. The observations in the tail show that this deflected field is transported to the downstream side of the comet, and then eventually stretches out to the more regular tail structure.

Around apoapsis, for $R \geq 500~\text{km}$, the clock angle, defined as:

25 $$\begin{aligned} \phi _{\mathrm{cl}} = \tan ^{-1} \left \{ \frac{B_{\mathrm{z}}}{B_{\mathrm{y}}} \right \}, \end{aligned}$$

showed unexpected behaviour. In Fig. 6 of Volwerk et al. (2018) the unwrapped clock angle is presented, and it shows a continuously increasing angle $\phi _{\mathrm{cl}}$. Even when the spacecraft turns around at apoapsis $\phi _{\mathrm{cl}}$ increases, which means that RPC-MAG is measuring a traveling rotational wave along the magnetic field. Using the frequency of the wave and the velocity of Rosetta outbound ($\omega _{\mathrm{h}} \approx 5.65^{ \circ}\text{/h}$, $v_{\mathrm{ros}} \approx 1.3~\text{m/s}$) and inbound ($\omega _{\mathrm{h}} \approx 5.83^{\circ}\text{/h}$, $v_{\mathrm{ros}} \approx 3~\text{m/s}$), a phase velocity of the wave was found of $v_{\mathrm{h}} \approx 136~\text{m/s}$. This behaviour of the clock angle is not present in the solar wind, and thus must be from cometary origin. However, with an average magnetic field of $B_{\mathrm{m}} \approx 10~\text{nT}$, this velocity does not correspond to the Alfvén velocity, nor does the gyro frequency correspond to that of any major species.

### Collisionopauses

A collisionopause is a boundary in the comet-solar wind interaction region where collisions first become important inside of the boundary (Mendis et al. 1986; Cravens 1989, 1991). Cravens (1991) outlined various types of collisionopause for both ions and electrons depending on the collision processes involved. To date, three boundaries have been observed by spacecraft at comets that have been proposed to be collisionopause boundaries: the cometopause (Gringauz et al. 1986b; Gombosi 1987; Coates 1997), the cometary ion collisionopause (Mandt et al. 2016, 2019), and the electron exobase (Henri et al. 2017). Two of the three boundaries are illustrated in Fig. 34. The electron exobase is observed in the region near the boundary of the diamagnetic cavity (Fig. 44).

Since a collisionopause is the location where collisions between plasma and neutrals dominate the plasma dynamics (Mendis et al. 1986), this boundary is similar to an exobase of an atmosphere, or the boundary used in aeronomy where collisions dominate the dynamic of the gas. Therefore, we can estimate the location of a collisionopause in a similar manner to how the exobase location is determined. An exobase is located where the ratio of the mean free path to the scale height, is equal to one. In the case of a collisionopause, the plasma mean free path, $\lambda $, is estimated as

26 $$ \lambda =\dfrac{1}{(n_{n}\sigma _{c})}, $$

where $n_{n}$ is the local neutral density and $\sigma _{c}$ is the momentum transfer cross-section of the relevant collision. According to Edberg et al. (2015) the plasma scale height at 67P observed between August 2014 and February 2015 was equal to the distance from the comet, $r$. More generally, Beth et al. (2019) showed that the plasma density asymptotically follows $\sim 1/r$, and therefore the scale height is $\sim r$. Assuming that this scale height is effective for the duration of the Rosetta mission during which the outgassing rate was high enough to form collisionopause boundaries, we can estimate the location of a collisionopause as the point where the distance from the comet is equal to the plasma mean free path. Taking into account that $n_{n} \propto r^{2} $, we can derive the mean free path to be

27 $$ \lambda =r=n_{s/c}\sigma _{c} r_{s/c}^{2}, $$

where $r_{s/c}$ and $n_{s/c}$ are the spacecraft cometocentric distance, and the locally measured neutral density. This expression is equal to Eq. (29), if the relevant cross-section is that of electron-neutral collisions.

There are several sources of uncertainty when attempting to predict the location of a collisionopause, the greatest of which is the momentum transfer cross-section. These cross-sections depend on the composition of the ions and neutrals as well as the energy of the ions and electrons (Johnson et al. 2008), and are in general poorly understood (Mandt et al. 2019). When evaluating spacecraft flybys of comets, Itikawa and Mason (2005) assumed that the electron-neutral momentum transfer cross-section was $\sim 5 \times 10^{-16}~\text{cm}^{2}$ for 5 eV electrons, while Mendis et al. (1986) estimated the ion-neutral cross-section to be between $2 \times 10^{-15}~\text{cm}^{2}$ for solar wind protons and $8\times 10^{-15}~\text{cm}^{2}$ for mass loaded solar wind with a bulk composition of $\text{H}_{2}\text{O}^{+}$. Mandt et al. (2019) conducted a review of cross sections for reactions between water ions and water neutrals and showed that the energy-dependence of these cross sections means that the energy of the mass loaded solar wind ion flow determines the location of a cometary ion collisionopause boundary. However, based on the detailed analysis of solar wind ion cross sections by Simon Wedlund et al. (2019), the assumption of cross sections for the solar wind and cometary neutrals of $\sim 2 \times 10^{-15}~\text{cm}^{2}$ for solar wind protons is reasonable.

**The cometopause** is described as the boundary where the ion composition in the solar wind ion flow changes from predominantly solar wind ions ($\text{H}^{+}$, $\text{He}^{2+}$) to predominantly cometary ions ($\text{O}^{+}$, $\text{OH}^{+}$, $\text{H}_{2}\text{O}^{+}$, $\text{H}_{3}\text{O}^{+}$, etc.) (Gringauz et al. 1986b; Gombosi 1987; Coates 1997). Some have disputed the existence of this boundary (Rème et al. 1994), while others suggest that it is a permanent feature (Gringauz and Verigin 1991; Sauer et al. 1995). The cometopause at previous comet flybys was characterized by a decrease in electron velocity and temperature, an increase in ion densities and deflection of ions (Rème et al. 1988; Cravens 1989). It is thought to be the beginning of the magnetic pileup region (d’Uston et al. 1988). The question remains whether the cometopause could be a collisionopause from ion-neutral charge transfer reactions such as $\text{H}_{2}\text{O}_{\text{fast}}^{+} + \text{H}_{2}\text{O} \rightarrow \text{H}_{2}\text{O}_{\text{fast}} + \text{H}_{2}\text{O}^{+}$ as proposed by Cravens (1989). Based on the cross section for this reaction and the production rate of $6 \times 10^{27}~\text{s}^{-1}$ measured by MIRO during the dayside excursion (Mandt et al. 2016), Rosetta should have crossed the cometopause before crossing the cometary ion collisionopause, which had a larger cross section. As shown in Fig. 34, the observed crossings of the cometopause for Giotto at 1P/Halley and 26P/Grigg-Skjellerup were at a much greater distance from the nucleus than the cometary ion collisionopause, and thus the location of the cometopause based on spacecraft flyby observations is well outside of the region explored by Rosetta, which agrees with ion composition observations made during high outgassing periods (Behar et al. 2017). Therefore, it is not likely that the cometopause is a collisionopause as speculated previously.

**Fig. 40:**
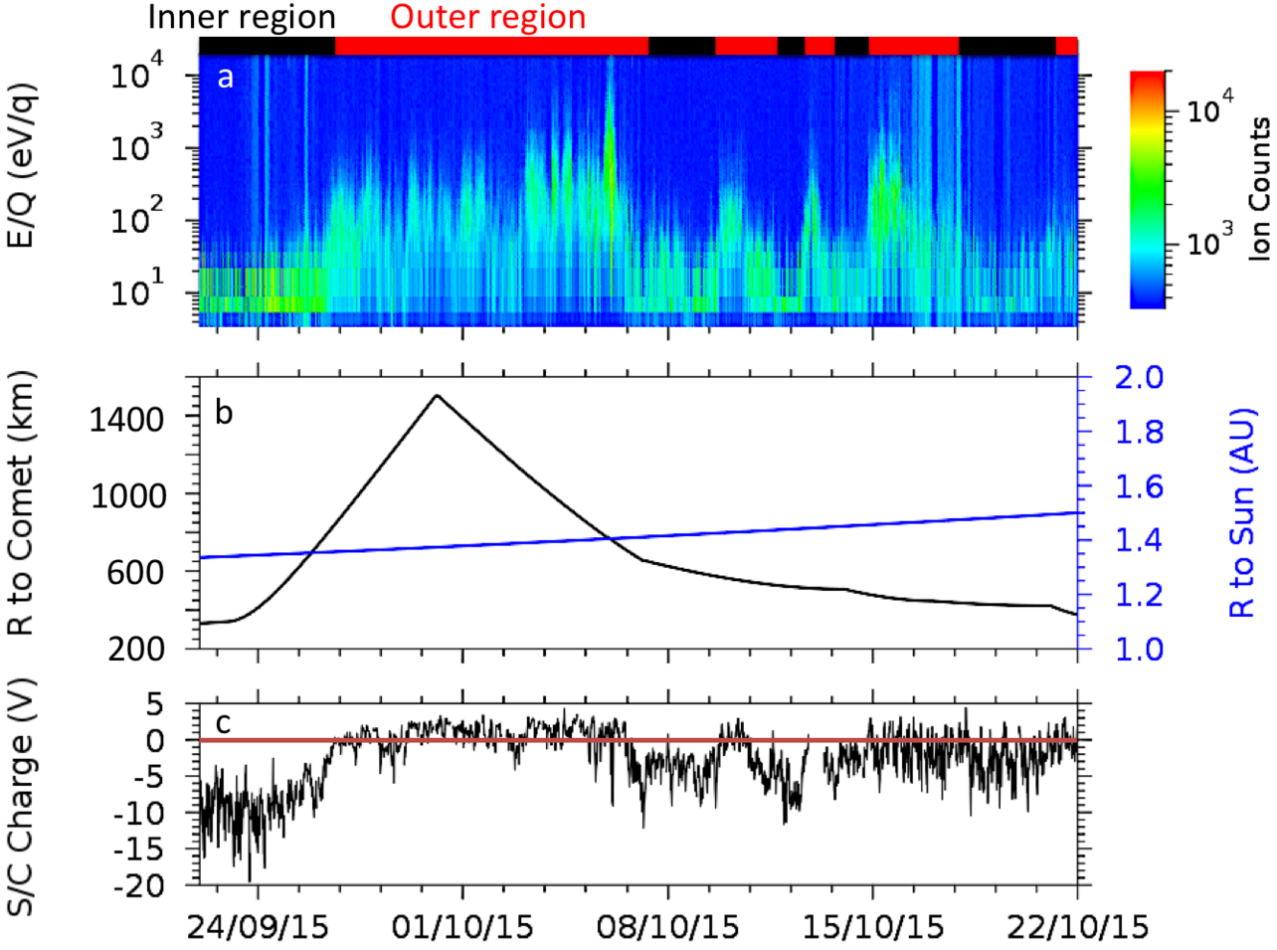
Observations of crossings of the ion-neutral collisionopause during the excursion to 1500 km in September and October 2015. The bar at the top of the figure indicates whether the spacecraft was in the inner (black) or the outer (red) region. In the outer region ions with energies greater than 50 eV are observed (panel **a**). Ion energies in the inner region are observed at the spacecraft potential (panel **c**), which varied between 0 and $-20~\text{V}$. The boundary between the two regions appears in some crossings to be sharp, such as on 15 October, while at other points to be broad. The sharp boundary crossings likely appear to be sharp because the solar wind dynamic pressure increased due to an event such as a CIR or CME, causing the boundary to move across the spacecraft (see Mandt et al. 2016, for details). From Mandt et al. (2019), reproduced with permission ©ESO

**The cometary ion collisionopause** was first observed by Giotto at 1P/Halley (Schwenn et al. 1988; Altwegg et al. 1993) and later by Deep Space 1 at 19P/Borrelly (Young et al. 2004), but was not identified as a boundary until after Rosetta observations detected and characterised this boundary at 67P (Mandt et al. 2016, 2019). Observations made by RPC-IES between January 2015 and February 2016 showed the formation of two regions based on ion energies: an inner and an outer region. These regions were separated by a boundary that appeared at times to be sharp, and at other times to be broad as illustrated in Fig. 40. The inner region is characterised by low energy ions that appear at an energy near the value of the spacecraft potential, which can be seen in panels (a) and (c) of Fig. 40, while the outer region is characterised by relatively high energy ions that RPC-ICA shows to be water-group ions (Nilsson et al. 2015a,b; Behar et al. 2016b). Their high energy suggests that they are cometary ions that have been picked up in the solar wind flow. The boundary was first detected in April 2015, when the solar wind disappeared from the RPC-IES ions and the energy of the water group ions began to vary between the value of the spacecraft potential and values above 50 eV. This variability was at times related to the spacecraft distance from the comet, but at other times may have been due to changes in solar wind dynamic pressure based on models extrapolating measurements made at Earth to the Rosetta location (Mandt et al. 2016). Over time, the outer region was observed less often than the inner region and by the end of July 2015 Rosetta remained mostly in the inner region. The next time that Rosetta moved into the outer region was during the dayside excursion that took place in September and October 2015, at which time the spacecraft travelled from within 300 km of the nucleus out to 1500 km from the nucleus and then back. During the excursion, Rosetta crossed the boundary and remained outside of the boundary for a couple of days as shown Fig. 40, thus confirming that the boundary is a permanent feature rather than a transient one. The electron densities were enhanced in the inner region while the magnetic field magnitude was reduced. This boundary was located well outside of the diamagnetic cavity (Goetz et al. 2016b).

**Fig. 41:**
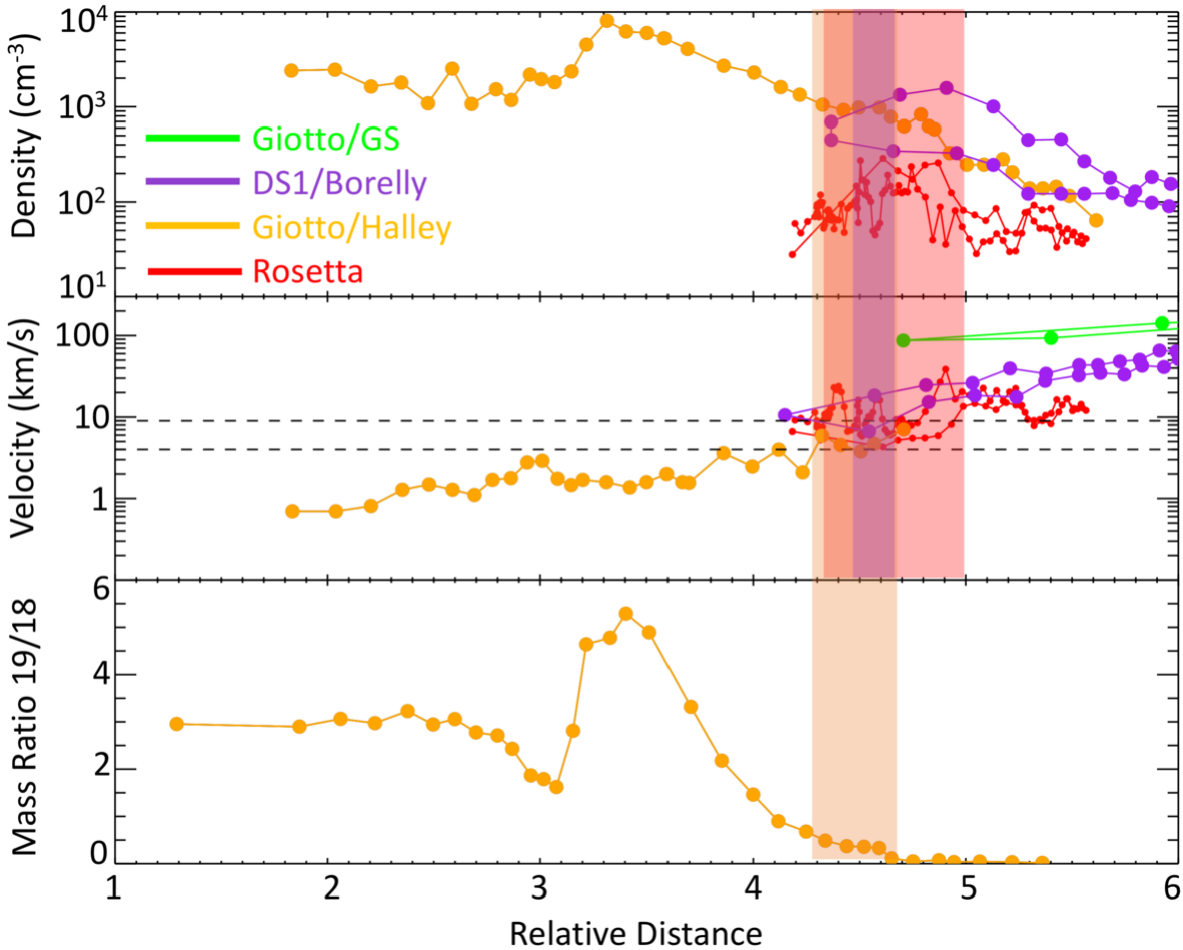
Comparison of plasma observations as a function of the distance from the nucleus scaled by the production rate $\times10^{-27}$ as observed by Rosetta during the dayside excursion, Giotto during flybys of 1P/Halley (Altwegg et al. 1993) and 26P/Grigg-Skjellerup (Goldstein et al. 1994) and Deep Space 1 during the flyby of 19P/Borrelly (Young et al. 2004). The top panel shows the total ion number density for the flybys and the Rosetta electron density. The centre panel illustrates the ion bulk velocity, and the bottom panel shows the ratio of ions with mass 19 ($\text{H}_{3}\text{O}^{+}$) to ions with mass 18 (mostly $\text{H}_{2}\text{O}^{+}$ with some uncertain contribution from $\text{NH}_{4}^{+}$). The horizontal dashed lines in the centre panel represent the estimated bulk velocities for RPC-IES observations of collisionopause crossings after correcting for spacecraft charging effects. The shaded regions indicate where Rosetta, DS1, and Giotto at 1P/Halley crossed into a similar range of velocities indicating crossing of the cometary ion collisionopause. The ion velocities at 26P/GS did not show an indication of crossing of this boundary. From Mandt et al. (2019), reproduced with permission ©ESO

It is valuable at this point to compare the Rosetta observations during the dayside excursion with observations made at 1P/Halley, 26P/Grigg-Skjellerup, and 19P/Borelly. We illustrate in Fig. 41 total ion or electron density, the ion velocity, and the ratio of mass 19 to 18 ions for these four mission observations. The cometary ion collisionopause observed by Rosetta strongly resembles a drop in ion velocity observed by Giotto at 1P/Halley (Schwenn et al. 1988) in a region described as an ion pileup region (Balsiger et al. 1986). The Giotto observations of the mass 19/18 ratio shown in the bottom panel of Fig. 41 demonstrate that $\text{H}_{3}\text{O}^{+}$ became the dominant ion species in the ion pileup region (Balsiger et al. 1986; Schwenn et al. 1988). This is indicative of a reaction taking place in this region forming $\text{H}_{3}\text{O}^{+}$ from $\text{H}_{2}\text{O}^{+}$. Based on the observations shown in panels (a) and (b) of Fig. 40 it appears that the ions begin to pile up inside of the point where we identify the cometary ion collisionopause with the shaded region. Inside of this, panel (a) shows that the ion densities decrease well inside of this boundary, possibly indicating the electron exobase, or the electron cooling boundary, where ion loss rates due to electron recombination will increase as a result of electron cooling.

**The electron exobase** is the electron-neutral collisionopause (Mandt et al. 2016). It is also referred to as the electron cooling boundary (Eriksson et al. 2017; Henri et al. 2017), because it is the boundary outside of which electrons are no longer collisional. As with the cometary ion collisionopause, this boundary is not sharp but is instead a region of gradual transition. This is complicated by the fact that electrons may be cooled within the electron exobase, but cold electrons are found everywhere in the plasma environment. This is because of transport of electrons (Gilet et al. 2020). Evaluation of Rosetta observations show that this boundary is located near the diamagnetic cavity boundary (see Fig. 34) (Henri et al. 2017). For a more detailed discussion see Sect. 4.6 and 2.3.5.

We described here three possible collisionopause boundaries. The boundary closest to the nucleus would be the electron exobase, which is located near the diamagnetic cavity that is shown in Fig. 34. This Figure shows that observations have placed the cometary ion collisionopause farther from the nucleus than the diamagnetic cavity and that the cometopause is expected to be even farther away.

### Diamagnetic Cavity

When Biermann et al. (1967) suggested the mechanism of mass-loading of the solar wind at a comet (see Eq. (21)), one of the outcomes of the model was the deceleration of the solar wind until the velocity eventually reached zero somewhere upstream of the comet. Although the magnetic field was not included in the early models, it became clear that the magnetic field was tied to the solar wind (electrons) and thus would also come to a stop. With the assumption that a comet nucleus is not magnetised, the consequence of this would be the formation of a field-free diamagnetic cavity around the densest part of the cometary ion cloud around the nucleus.

The Active Magnetospheric Particle Tracer Experiment (AMPTE) in 1984–1986 enabled the first observations of such a region. The experiment consisted of three spacecraft, one of which was equipped with Barium and Lithium canisters that could be released in the magnetosheath of Earth’s magnetosphere or in the solar wind. The expanding gas and its impact on the plasma was then monitored by the spacecraft and ground-based observations (Valenzuela et al. 1986; Luehr et al. 1986; Haerendel et al. 1986; Rodgers et al. 1986). Just after the release of the Barium canister a region of zero-field could be observed for about a minute. This was the first evidence that addition of a gas cloud to a plasma can expel the magnetic field entirely from a certain region.

The advent of comet 1P/Halley then brought a number of flybys of the comet, of which ESA’s Giotto spacecraft achieved the lowest altitudes, with a closest approach of $\sim 600~\text{km}$. As predicted, this was well within the bounds of the diamagnetic cavity and Neubauer et al. (1986) reported on magnetic field measurements while crossing into a field-free region at $\sim5000~\text{km}$. Just outside the diamagnetic cavity Neubauer (1987) showed that a magnetic pile-up region similar to that at Mars or Venus exists, with maximum field strengths around $65~\text{nT}$. This measurement presented the only detection of the diamagnetic cavity at a comet up until the new measurements by Rosetta.

In preparation for the Rosetta mission a number of simulations were used to constrain the diamagnetic cavity size and plasma parameters. Single fluid MHD simulations predict the diamagnetic cavity where ion-neutral friction equals magnetic pressure, which is at about $r=50~\text{km}$, where the plasma velocity is also very close to zero. Multi-fluid MHD simulations show that an asymmetric outgassing profile (higher density on sunward side) extends the cavity further out on the sunward side (Huang et al. 2016). Furthermore, Kelvin-Helmholtz instabilities of the boundary can develop perpendicular to $\vec{B}$ (Rubin et al. 2012, 2015). The simulations also show that the solar wind only vanishes close to the diamagnetic cavity boundary (DCB). Hybrid simulations by Koenders et al. (2015) can confirm many of the MHD findings, but add more information in terms of ion dynamics. For example, they show a clear separation of solar wind ion cavity and diamagnetic cavity. Some accelerated cometary ions are visible even inside the diamagnetic cavity. Similar to Kelvin-Helmholtz instabilities, there are density filaments propagating along the boundary, however, these are not visible in the magnetic field, but that is attributable to numerical effects (high numerical diffusion for simulation stability) (Koenders et al. 2015). In summary, the diamagnetic cavity at comet 67P was expected to be observable only close to perihelion ($\pm2$ months) and was rather small with a radius of around $50~\text{km}$. Due to navigational constraints of the Rosetta spacecraft, the altitude of the spacecraft during those months had to be raised to several hundred km (see Sect. 3.1) and thus an in-situ detection of the diamagnetic cavity was thought to be unlikely.

However, as reported in Goetz et al. (2016a,b) clear signatures of a magnetic field free region could be observed at 67P by Rosetta. It should be noted here, that the offset of the magnetometer was in the order of a couple of nT and thus in the initial data, the field was not observed as zero, instead the signatures were recognisable by a conspicuous absence of all field fluctuations that are predominant in the magnetic field around 67P close to perihelion (see Sect. 2.3.7). Assuming the field to be zero in the cavity has served as a good way to recalibrate the instrument, but on the other hand the exact field value in the cavity is only constrained to $\pm 3~\text{nT}$ per component (Goetz et al. 2016a). Over 700 separate events have been reported since, between April 2015 and February 2016. This corresponds to heliocentric distances below $2~\text{AU}$ ($2.4~\text{AU}$) inbound (outbound) and gas production rates above $10^{27}~\text{s}^{-1}$ (Goetz et al. 2016a; Goetz 2019).

**Fig. 42:**
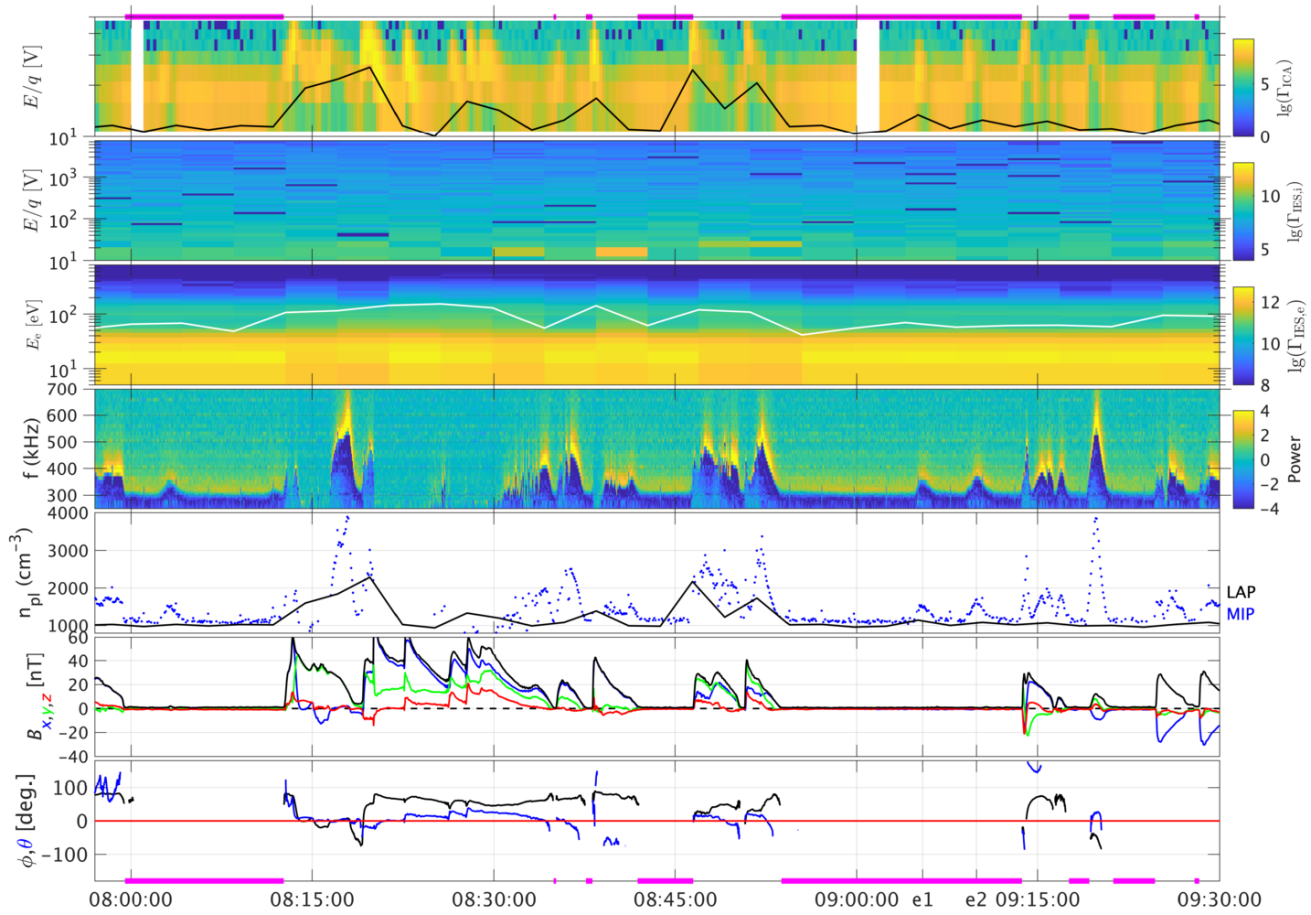
RPC observations of the plasma from 30 July 2015. From top to bottom: ICA heavy ions (high time resolution mode and negative spacecraft potential in black), IES ions, IES electrons, MIP spectra, plasma density from LAP and MIP, magnetic field, cone and clock angle of the magnetic field. The white line on the IES electrons indicates the summed flux over the $80\text{--}120~\text{eV}$ interval. The horizontal magenta bars mark the time that Rosetta was measuring inside the diamagnetic cavity. *e1* and *e2* mark the times of two density enhancements in the diamagnetic cavity

Figure 42 shows a series of cavity signatures observed by the RPC instruments on 30 July 2015, close to perihelion. The spacecraft was $180~\text{km}$ from the nucleus ($\vec{r}_{\text{CSEQ}}=(0,80,150)~\text{km}$). The quick succession of events already illustrates a peculiarity of the Rosetta measurements: Rosetta is a slow spacecraft, so whenever the spacecraft enters the diamagnetic cavity it is attributed to the boundary moving (not the spacecraft). These events illustrate typical behaviour of the plasma. The plasma outside is characterised by a series of asymmetric, steepened waves which are visible in the magnetic field, as well as in the MIP plasma density and the ICA heavy ion energy spectra (Stenberg Wieser et al. 2017; Hajra et al. 2018b). The signature of these steepened waves is mirrored in the magnetic field at the boundary of the diamagnetic cavity where the decrease is always less steep than the increase when Rosetta leaves the cavity (Goetz et al. 2016a). The magnetic field is oriented mostly along the $x$-axis (cone angle $\phi =90^{\circ}$) whereas the clock angle $\theta $ varies more frequently. The magnetic field direction can flip when traversing a cavity (Goetz et al. 2016b). This is in accordance with the nested draping shown in Sect. 4.4. The IES ion measurements in this interval do not show much variation due to inopportune pointing. The electron spectra show little variation, except for a dropout in electrons of energies between $\sim 60~\text{eV}$ to $\sim 200~\text{eV}$. Even with poor temporal resolution this decrease is visible in the summed electron flux. Nemeth et al. (2016) and Madanian et al. (2017) speculated that this signature was due to the inability of field-aligned electrons to penetrate the diamagnetic cavity boundary. Madanian et al. (2020) calculated electron pitch angle distributions and found that the electrons in this energy range become increasingly field aligned as they enter the DCB. Inside the cavity, the unmagnetised plasma density has been shown to scale very well with the neutral density (Henri et al. 2017). The typical plasma density observed along Rosetta’s trajectory close to perihelion is $\sim 1000~\text{cm}^{-3}$, about a factor $1.5\text{--}2$ higher outside the cavity (Hajra et al. 2018b).

The absence of the magnetic field also influences the wave environment, for example Gunell et al. (2017a) found ion-acoustic waves near the DCB and speculated that a current close to the boundary would be necessary to generate the waves. Other ion-acoustic waves are thought to be transmitted Lower hybrid waves (Madsen et al. 2018). For more details, see Sect. 2.3.7.

Although the unmagnetised plasma is observed to be quiet within the diamagnetic cavity (Henri et al. 2017), isolated events of dense plasma are sporadically observed. Two such events are marked *e1* and *e2* in Fig. 42. Hajra et al. (2018a) found that theses plasma enhancements are not associated to neutral gas density fluctuations, but that both their structure and density are instead very similar to the steepened wave structures outside the diamagnetic cavity. They conclude that these blobs of plasma originate outside the cavity and are probably transmitted through the DCB. The scale length is $100\text{--}800~\text{km}$, thus larger than the cavity itself, indicating it should be a global enhancement of the plasma density within the diamagnetic cavity (Hajra et al. 2018a).

These plasma enhancements are also visible in the ion spectra as higher energy and fluxes (Masunaga et al. 2019). Their properties are similar to the ion energy enhancements associated with the steepened waves, although they occur a factor of 10 less inside the cavity. They occur more frequently at lower gas production rates. The ion velocity is tailward and slightly radial. This affirms that the plasma can probably penetrate the DCB.

The shape and size of the diamagnetic cavity at 67P are hard to constrain, because only one point of measurement exists and in order to detect the diamagnetic cavity, the boundary needs to move and cannot be in a stationary state. Thus, models are needed to provide context. Historically, the problem of diamagnetic cavity formation has been viewed as a game of pressures: the magnetic pressure outside of the cavity needs to be balanced by another pressure on the inside. For the AMPTE artificial comet, it was determined that the gas pressure of the electrons in the added plasma was enough to balance the upstream magnetic pressure (Luehr et al. 1988). However, with the measurements by Giotto, it became clear that the situation there was somewhat different. Neither the electron pressure nor the ion dynamic pressure changed significantly at the diamagnetic cavity boundary (Cravens 1986). Thus a new mechanism had to be found to explain why the magnetic field was unable to diffuse into the diamagnetic cavity. Cravens (1987) presented a model in which the ion-neutral drag was proposed as that mechanism:

28 $$ \frac{\partial}{\partial r}\left (\frac{B^{2}}{2 \mu _{0}}\right )= \rho _{i} k_{in} n_{n} \left (\vec{u}_{i}-\vec{u}_{n}\right )_{r} $$

Several assumptions were made: first, photochemical equilibrium was assumed and second, the ion velocity was set to zero in the integration region. These assumptions are thought to be valid at comet 1P/Halley where large neutral densities due to the high gas production rate imply that the ions are efficiently coupled to the neutrals and do not move far between being created and annihilated by recombination. With this model the magnetic field in the boundary region could be calculated dependent on $r$ and matched reasonably well with the measured profile. The diamagnetic cavity size was predicted to be $4400~\text{km}$, which also fit well with the measurements.

The sheer number of diamagnetic cavity crossings at 67P is useful to constrain the dependence of the diamagnetic cavity size and shape, depending on a variety of parameters. Goetz et al. (2016a) found that the size increases with gas production rate and, similar to simulations as well as observations at 1P/Halley, that the boundary is highly unstable. An instability is necessary to explain the quick succession of events as shown in Fig. 42 as well as the magnetic field orientation outside of the diamagnetic cavity. An elliptical shape with overlaying boundary oscillation fits the boundary normal distribution. All in all, the size of the diamagnetic cavity was much larger than expected, up to $400~\text{km}$ instead of just $50~\text{km}$ according to simulations and the models based on ion-neutral friction.

**Fig. 43:**
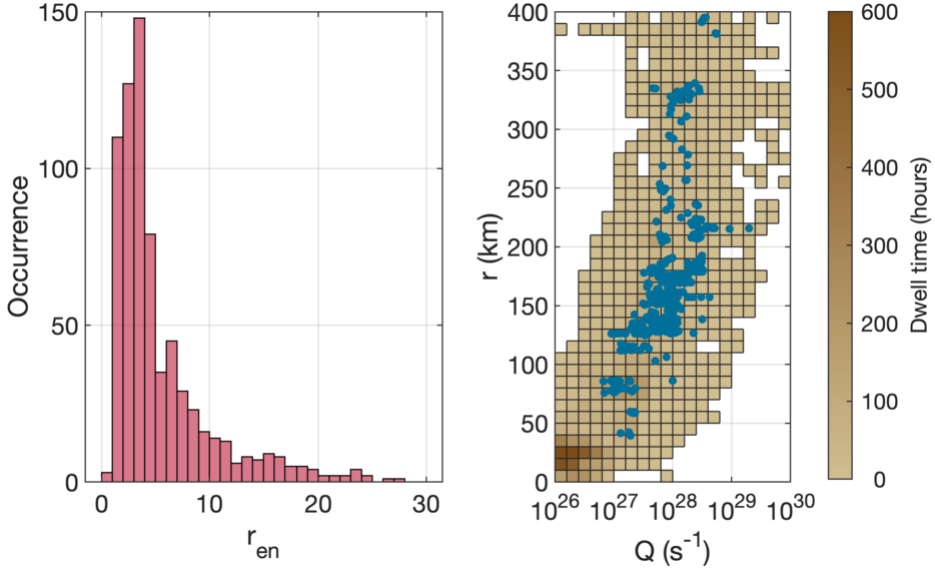
Left: Occurrence of cavity measurements over the spacecraft-comet distance in electron collision lengths. Right: spacecraft-comet distance over gas production rate (derived from in-situ measurements and a Haser model). The colour scale gives the dwell time of the spacecraft over the entire Rosetta comet phase, and the blue points mark the points at which a cavity was detected

Henri et al. (2017) examined the plasma density inside the DCB in more detail and found that the size of the diamagnetic cavity scales very well with the electron exobase (or electron collisionopause), meaning that more cavities are detected when the spacecraft is close to the region where the electrons are more efficiently coupled to the neutrals (see also Sect. 2.3.2 and Fig. 43). The distance of Rosetta can be defined in terms of electron collision length scales:

29 $$ r_{en} = r / (n_{n} r^{2} \sigma _{en}). $$

It is therefore possible that the mechanism that keeps the magnetic field at bay is related to the electron neutral collisions. As a way to explain the quiet and uniform plasma inside the diamagnetic cavity they also suggested that an instability such as a Rayleigh-Taylor instability could move the boundary quite rapidly. A four-fluid Hall MHD simulation shows that the introduction of the Hall term induces an asymmetry in the diamagnetic cavity size perpendicular to the upstream magnetic field and finds small regions of low magnetic field that are detached from the diamagnetic cavity that are possibly caused by reconnection (Huang et al. 2018). These regions could also be interpreted as protrusions from a global structure, similar to instabilities.

Modelling the diamagnetic cavity size at 67P based on the ion-neutral friction model gave satisfactory results if the maximum field in the pile-up region is chosen such that the observations fit the model (Nemeth et al. 2016; Timar et al. 2017; Madanian et al. 2017; Nemeth 2020). This is unexpected, because this model (first derived by Cravens 1987) makes assumptions that do not apply at comet 67P: According to Beth et al. (2019) photo-chemical equilibrium does not hold in the coma at 67P, because transport is also important for the ion processes close to the cavity (see also Sect. 2.2.4). This can be shown by comparing the observed ion and neutral gas velocities. If the ion velocity is larger than the neutral velocity, this means that the ions have been accelerated and transport plays a role, and the ion-neutral collisions cannot sufficiently cool the ions. Unfortunately, reliable observations of slow ions are difficult with Rosetta, because of an often negative spacecraft potential (Odelstad et al. 2015; Bergman et al. 2020). Nonetheless, Odelstad et al. (2018) and Vigren et al. (2017) used independent methods to find that the ion velocity close to the diamagnetic cavity is $2\text{--}4~\text{km/s}$ with values on the lower edge of that range inside the diamagnetic cavity. As for the density, the ion velocity has a much higher variability outside of the cavity and decreases with cometocentric distance. From wake effects it can also be deduced that the bulk plasma flow is radial and supersonic. An ion velocity higher than the neutral velocity is in direct contradiction with the assumption that ions and neutrals are collisionally coupled and thus in direct contradiction with the model suggested by Cravens (1987). Vigren and Eriksson (2017) showed that such a discrepancy can be explained by low collision rates due to low neutral gas densities and propose that an ambipolar electric field (see also Sect. 4.1) can accelerate ions to the observed velocities. Fuselier et al. (2016) also pointed out that the unexpected $\text{H}_{2}\text{O}^{+}/\text{H}_{3}\text{O}^{+}$ ratio can be explained by an ambipolar field inside the diamagnetic cavity. It should also be noted that the definition of the collisionopause as a sharp boundary that separates a region where neutrals and ions are coupled from a region where they are not coupled is inappropriate, since collisions are a process that occurs on length scales comparable with the diamagnetic cavity size (Vigren and Eriksson 2019). As of now, it is unclear which process is responsible for deterring the magnetic field from diffusing into the diamagnetic cavity.

The inner coma is often dominated by warm electrons from photoionisation, but inside the diamagnetic cavity a second, colder ($<0.1~\text{eV}$) population is found (Gilet et al. 2017; Odelstad et al. 2018; Engelhardt et al. 2018a). The cold electrons occur more frequently near the electron exobase and where the neutral gas is densest (e.g. due to asymmetric outgassing). The same ambipolar field that accelerates the ions retards the electrons and couples them more efficiently to the neutral gas via collisions. However, the appearance of cold electrons is also related to the direction of the convective electric field outside the diamagnetic cavity (Edberg et al. 2019a). The cold electrons are preferentially detected in the $-E_{conv}$ hemisphere, the same applies for the diamagnetic cavity (see also Sect. 2.3.2).

**Fig. 44:**
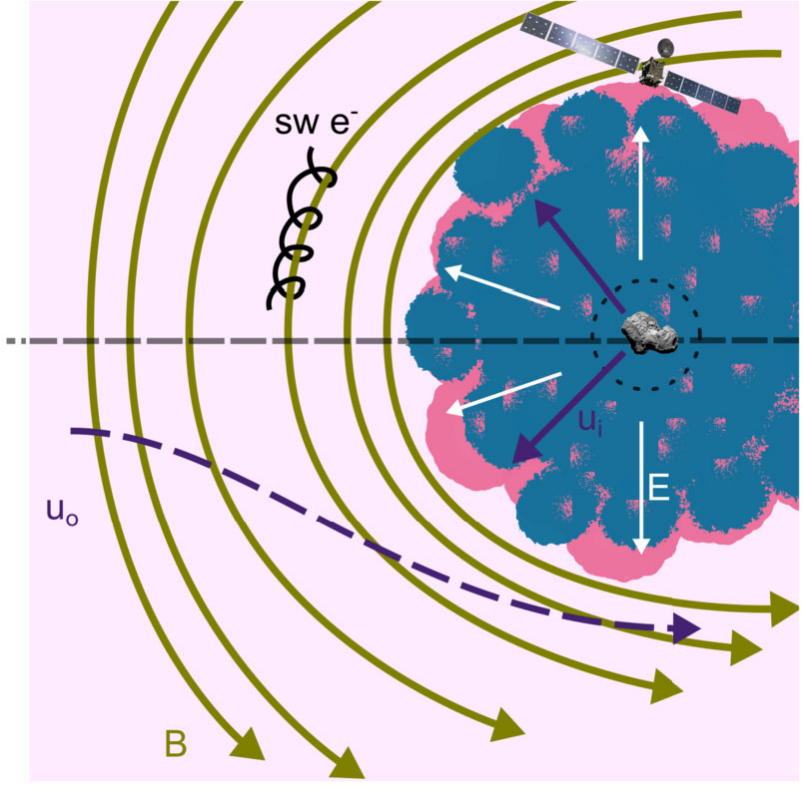
Sketch of the diamagnetic cavity at comet 67P. The magnetic field (green) is draped around the cavity and solar wind electrons (sw $\text{e}^{-}$) are moving along the field lines. Rosetta is located at the terminator, the most common configuration during the mission. Ion velocities are indicated in dark purple, with the accelerated ions outside the cavity moving anti-sunward and the newborn ions moving radially outward. The ambipolar electric field (white) is confined to the cavity. The cold (blue) and warm (red) ion populations are indicated as well. The light red background indicates the presence of warm electrons in the entire inner coma. The ion-neutral collisionopause is shown in black dashed lines

Figure 44 is an attempt at summarising the shape of and magnetic field and electron environment at the diamagnetic cavity. Although Rosetta observations of the diamagnetic cavity have added greatly to our understanding of the innermost coma, many open questions remain: What is the pressure balance at the boundary and what part does the changing plasma environment play in the movement of the DCB? How are electrons and ions cooled and/or accelerated in and near the cavity? What is the origin of the asymmetry in the in- and outbound crossing of the boundary? How are plasma enhancements transmitted trough the boundary and inside the diamagnetic cavity? Addressing these questions probably requires a multi-point measurement of the diamagnetic cavity, its boundary and upstream plasma conditions (Goetz et al. 2021).

## Conclusions and Outlook

### Conclusions

The plasma environment of comet 67P represents a new frontier in cometary plasma science. Due to the smaller outgassing rate compared to previously visited comets and the long mission duration, the Rosetta mission covered a new area in parameter space. At the comet, there are two plasma sources: the solar wind (mostly protons and electrons) and the cometary plasma, made up of heavy ions (mostly water) and electrons. Their densities mainly depend on heliocentric distance, though they are sometimes influenced by transient events, such as CIRs or cometary outbursts. The presence of these ions and electrons in the plasma stems from an equilibrium between sources (ionisation) and losses. The major net ionisation mechanisms are electron impact ionisation and photo-ionisation, with their relative importance changing over the two years covered by the Rosetta mission close to the nucleus. At larger cometocentric distances usually not explored by Rosetta, charge-exchange mechanisms are expected to play a prime role. The major plasma loss processes are transport and dissociative electron-ion recombination. Contrary to other cometary environments which are denser in their closest approach to the Sun, the outward transport of newborn ions remains an important process even near perihelion. FUV emissions can be used as a diagnostic tool for the neutral composition of the coma, electron-impact processes (including ionisation) and recombination processes. OI 1356 Å can be used to monitor the solar wind electrons beyond 2 AU from the Sun. Due to the large ion gyroradii compared to the size of the plasma environment, the kinetic behaviour of ions plays an important role for both the solar wind and cometary ions and can lead to pronounced asymmetries in large-scale structures. Several populations of electrons of different origin are present in the coma. Whereas solar wind electrons are typically warm (around $10~\text{eV}$), at large heliocentric distances (low outgassing) they are accelerated by an ambipolar electric field in the vicinity of the nucleus and reach energies of a few tens of eV responsible for ionisation and excitation of the cometary neutrals. Newborn cometary electrons are also warm (around $10~\text{eV}$), although some of them undergo cooling through, most likely, collisions with cometary neutrals. This yields a colder electron population, even at large heliocentric distances (3–4 AU from the Sun) when the outgassing is low and electrons were not anticipated to be coupled to neutrals.

In order to distribute momentum and energy throughout the plasma environment, waves are excited at all activity stages, and observed in the magnetic field, electric field, and plasma density measurements with periods ranging from milliseconds up to tens of minutes. Transients in the solar wind and outgassing rate influence the interaction on time scales of hours to days.

The Rosetta mission has enabled major advances in cometary plasmas. It was equipped with a range of plasma, field, gas, and spectral instruments and orbited the comet for over two years. This made it possible to study the evolution of the plasma processes as activity increased until perihelion and decreased again afterwards. In addition, laboratory measurements of reaction rates and emissions are integral to delivering input for models, both analytical and numerical. While it is impossible to recreate the large-scale structures of the cometary plasma, laboratory experiments on smaller scales can give valuable insight into plasma processes. Numerical models are also a powerful tool to interpret and complement the observations. However, models and their input need to be chosen carefully to ensure they are suitable to the situation. Unfortunately, no model can adequately treat both large and small scales alike.

Large-scale structures span scales above or at the gyroradius of the cometary ions and can be used to identify distinct regions, with different properties, in the plasma environment. A classical bow shock, as known from earlier cometary missions, was not observed at 67P due to trajectory constraints, but there is evidence of the existence of a bow shock in the ion spectra. An infant bow shock, an asymmetric structure that could be a precursor of a real bow shock, was detected. Deflection of solar wind ions as a result of mass-loading creates a solar wind ion cavity, a region devoid of solar wind ions that is persistent at high gas production rates. Picked up ions continue to carry the momentum into the solar wind ion cavity.

Large-scale electric fields include the convective, ambipolar and polarization electric field. They affect both the ions and electrons, and are important for instabilities and wave generation. The magnetic field is draped around the inner coma, often in a nested field pattern that was also observed at other comets. The draping follows the direction of the deflected solar wind, even in the near-nucleus tail. A diamagnetic cavity, a region devoid of any magnetic field, was observed hundreds of times and was found to be larger than expected. The boundary of this region is highly unstable. Cold electrons are always observed in the cavity, while electrons from the solar wind cannot enter. The magnetic field is close to zero in the diamagnetic cavity and magnetic field oscillations cease. Rosetta was always outside of the electron collisionopause, the region where electrons couple efficiently with neutrals assuming radial transport, but it observed the ion-neutral collisionopause on multiple occasions.

**Fig. 45:**
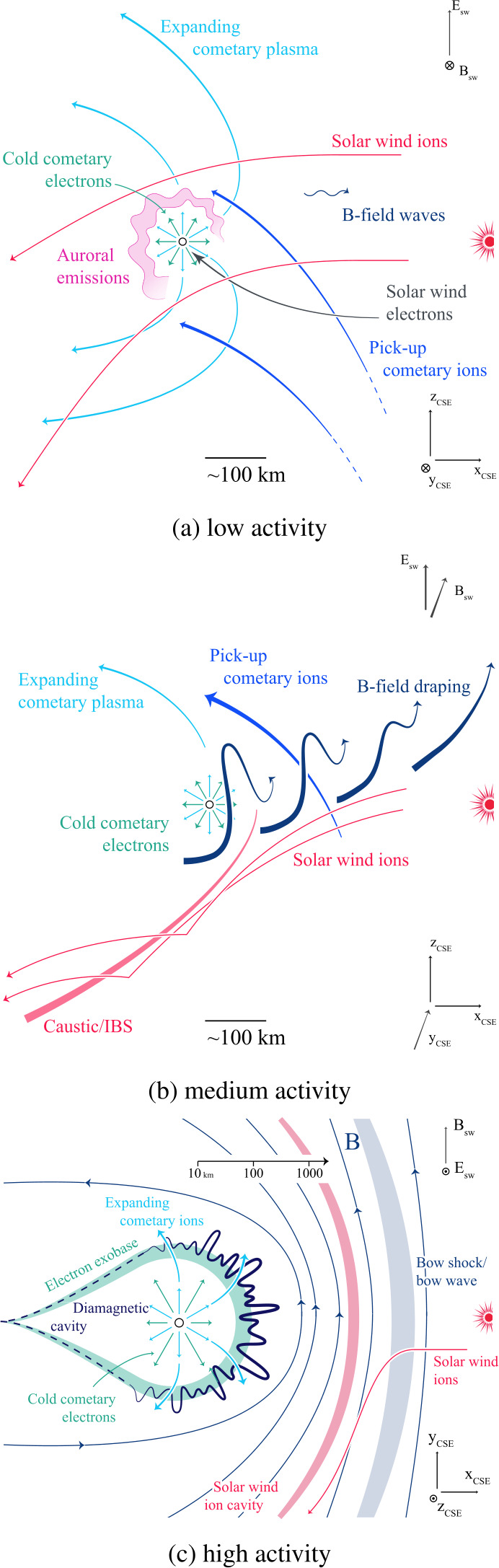
Sketch of the plasma environment at 67P at three different activity stages as defined in Sect. 1. Note that the sketch is in a CSE system, aligned with the magnetic and convective electric fields and that the cross-sections are different for the three panels. While the structures are approximately to scale with respect to each other for the low and medium activity cases. The high activity case is not to scale with regards to the other stages and the scale is approximately logarithmic with respect to the radial distance to the nucleus

Figure 45 is an attempt to summarise and overview the different key plasma processes described above. It shows the most important particle trajectories, fields and phenomena for the three activity stages as defined in the introduction (Sect. 1). Note that electric fields and most waves were not included, because they are summarised in Figs. 35 and 18. It becomes clear that comets are exposed to a wide range of scenarios. As they move around in the solar system, the plasma conditions change as well as the activity of the comet. This gives rise to a plethora of different processes, whose importance is changing with the changing conditions. As an example, far from the Sun (low activity, panel a), solar wind ions and electrons penetrate deeply into the tenuous atmosphere where they can give rise to auroral emissions from the soft X-rays to the FUV. The intermediate case (panel b) is characterised by the beginnings of large-scale structures like the infant bow shock and magnetic field draping which are asymmetric due to the gyroradius effects of the ions. Closer to the Sun (panel c), an unstable diamagnetic cavity is formed and cold electrons dominate close to the nucleus, near the electron exobase. The solar wind ions are not able to penetrate the inner coma anymore and there is evidence for a classical bow shock upstream of the solar wind ion cavity. Comets therefore cover an extensive part of parameter space and present an excellent laboratory to study large scale structures as well as small scale interactions.

### Open Questions

Many questions pertaining to cometary plasma environments still remain open, we provide here a non-exhaustive list of questions directly related to the new results from Rosetta:

- How do waves and wave-particle interactions affect the cometary plasma?
- What are the exact plasma distribution and ion composition (up to 50 amu) with respect to cometocentric distance (in 3D), and how does it vary in time? Qualitative and global quantitative measurements have been made with Rosetta, but systematic quantitative in situ surveys need to be made to understand the detailed ion-neutral physico-chemistry of the coma.
- Why is there evidence for ion acceleration at 67P within the cavity but not at 1P/Halley?
- What is the plasma balance near perihelion around the diamagnetic cavity?
- What is the impact of electric field acceleration and dust charging on the plasma balance and vice versa?
- What is the energy distribution of electrons below the spacecraft potential?
- How are dissociative electron-impact emissions (e.g., OI 6300 Å and OI 1356 Å) affected by the near nucleus coma environment near perihelion?
- How do nested draping and dynamic draping relate to each other, is a true pile-up of nested fields created or is this just a result of a snapshot flyby?
- What is the large-scale structure of the ion tail? How do the Rosetta near-tail and the ICE far-tail combine to one general structure?
- How do kinetic aspects of the ion dynamics around a low-to-medium activity comet continuously evolve towards a more fluid behaviour at high outgassing activity, close to the Sun?
- To what extent are the position and shape of the solar wind ion cavity dictated by macroscopic ion kinetic dynamics (see Fig. 37) and by loss of solar wind density through charge exchange?
- What is the combined effect of the polarisation and ambipolar electric fields?
- What is the speed of the bulk of low energy ions accelerated by the ambipolar electric field near the nucleus?
- How can we describe the shape and size of the bow shock, depending on outgassing and ionisation?
- What are the processes behind energy conversion at the bow shock and how can we make predictions based on outgassing and ionisation?
- Is there a force balance at the diamagnetic cavity boundary, and if so what forces are involved? What is the shape and stability of the boundary?
- Is the cometopause a physical boundary or a gradual transition in the composition of the cometary ions?
- Cold electrons have been observed even when the predicted electron collisionopause is below the surface. What is the process to cool newborn, warm electrons?
- What is the role of (quiet) solar wind dynamics in the inner coma dynamics?

These questions can be addressed by spacecraft missions (Goetz et al. 2021), but also simulations (see Sect. 3.3) and laboratory measurements (see Sect. 3.1.3).

### Outlook

It is clear that the philosophical strive to improve our knowledge of the physical processes in the plasma at the comet, and more generally natural plasma over a full range of collisionality levels, requires space missions that provide data directly from the source. Goetz et al. (2021) outline open questions and propose mission concepts that would be able to (partially) answer them. By far the most complete mission profile is that of a dedicated multi-spacecraft mission to study a comet’s plasma environment. Following a comet as it makes its way through the solar system, while exploring simultaneously the plasma environment at multiple points with a full suite of plasma and fields, energetic particle, and gas and remote-sensing instruments would afford the opportunity to answer most of the currently open questions.

A somewhat more limited mission concept is that of piggy-backing one or more dedicated plasma spacecraft on a mission exploring the nucleus, e.g. a sample return or lander mission. This would allow for a separation of the nucleus centred instruments that usually prefer smaller cometocentric distances and the plasma centred instruments that would require larger variations in cometocentric distances. The advantage is a reduction in mission cost, while losing some of the multi-point capabilities that come with a full plasma mission profile.

Other, smaller experiments based on similar concepts as AMPTE, i.e. creating ion clouds in or near Earth’s magnetosphere, could also help understand microscopic processes like collisionality and pick up in a large scale system. This has the advantage of close proximity to Earth, but cannot address the questions on larger dynamics in a comet’s plasmasphere.

A first step in this direction is ESA’s Comet Interceptor mission (Snodgrass and Jones 2019), which will intercept a dynamically new comet with three spacecraft sometime in the 2030s. Since this mission will be a fast flyby with multi-point measurements of the magnetic field (though not of the plasma), it will give a valuable snapshot of the environment of a dynamically new object and will provide valuable clues as to the asymmetry of structures in the plasma. However, by its nature, Comet Interceptor will not be able to explore the evolution of the plasma environment, and will only cover one part of the enormous parameter space to be explored at comets. After the Giotto and Rosetta missions, Comet Interceptor is a promising next step to pave the way of multi-point, larger-class missions for exploring the exciting, challenging, and enriching plasma environment around comets, which has still so much to reveal.

## Footnotes

1 https://www.cosmos.esa.int/web/psa/psa-introduction.

2 Or, interchangeably, production rate.

3 In the case of 67P, a very irregular shape indeed, with two lobes of different radii. This is not an untypical feature as other comets, such as 1P/Halley, 8P/Tuttle, 19P/Borrely and 103P/Hartley 2, all exhibit a similar irregular shape, likely formed from slow collisions between primordial rubber piles in the early solar system (a so-called “contact binary”, see Davidsson et al. 2016).

4 The warm and hot electron populations are sometimes grouped together as “suprathermal”, in contrast to the “thermal” cold electrons discussed in Sect. 2.3.5. As the warm population appears to be close to Maxwellian, we have here taken the view that the only clearly non-thermal electron population is the hot part and therefore avoid using “suprathermal” when referring to cometary electrons.

5 Direct link to download the RPC User Guide: https://dms.cosmos.esa.int/COSMOS/doc_fetch.php?id=1274141, direct link to download the RPC Quick User Guide: https://dms.cosmos.esa.int/COSMOS/doc_fetch.php?id=3752622.

## Acronyms

67P: Comet 67P/Churyumov-Gerasimenko
ACAP: As close as possible
AMPTE: Active Magnetospheric Particle Tracer Experiment
AU: Astronomical unit
CIR: Corotating interaction region
CME: Coronal mass ejection
COSAC: Cometary Sampling and Composition experiment
CSE: Comet-Sun electric field frame
CSEQ: Comet-centred solar equatorial coordinate system
DCB: Diamagnetic cavity boundary
DR: Dissociative recombination
DS1: Deep Space 1
EI: Electron impact
ENA: Energetic neutral atom
ESA: European Space Agency
EUV: Extreme ultraviolet
FUV: Far ultraviolet
GIADA: Grain Impact Analyser and Dust Accumulator
HR: High resolution
ICE: International Comet Explorer
ICME: Interplanetary coronal mass ejection
IMF: Interplanetary magnetic field
LAICA: Lyman Alpha Imaging Camera
LHDI: Lower hybrid drift instability
LR: Low resolution
MAVEN: Mars Atmosphere & Volatile Evolution
MHD: Magnetohydrodynamics
MIRO: Microwave Instrument for the Rosetta Orbiter
NAVCAM: Navigation camera
OSIRIS: Optical, Spectrocopic and Infrared Remote Imaging System
PA: Photo absorption
PDS: Planetary Data System
PHIDRATES: Photoionization/dissociation rates
PI: Photo ionisation
PIC: Particle in cell
PIU: Plasma Interface Unit
PROCYON: Proximate Object Close Flyby with Optical Navigation
PSA: Planetary Science Archive
QDB: Quantemol database
ROMAP: Rosetta magnetometer and plasma monitor instrument
ROSINA: Rosetta Orbiter Spectrometer for Ion and Neutral Analysis
ROSINA-COPS: ROSINA-Comet Pressure Sensor
ROSINA-DFMS: ROSINA-double focusing magnetic mass spectrometer
RPC: Rosetta Plasma Consortium
RPC-ICA: RPC-Ion Composition Analyser
RPC-IES: RPC-Ion and Electron Sensor
RPC-LAP: RPC-Langmuir probe instrument
RPC-MAG: RPC-Magnetometer
RPC-MIP: RPC-Mutual Impedance Probe
RPWI: Radio & Plasma Wave Instrument
STR: Star Tracker
SWCX: Solar wind charge exchange
SWI: Solar wind impact ionisation
TIMED: Thermosphere Ionosphere Mesosphere Energetics and Dynamics
TIMED/SEE: TIMED/Solar EUV Experiment
UT: Universal time
UV: Ultraviolet
VDF: Velocity distribution function
VIRTIS: Visible and InfraRed Thermal Imaging Spectrometer
WAC: Wide Angle Camera
